# Supplementary material for: Mesenchymal stem cell-derived extracellular vesicle therapy in breast cancer: A systematic review and meta-analysis of in vitro studies
Source: Mol Ther Oncol. 2025 Dec 9;34(1):201107. doi: 10.1016/j.omton.2025.201107 (PMC12775850; doi:10.1016/j.omton.2025.201107)
Supplement: Document S1. Figures S1–S5 and Tables S1, S2 [file mmc1.pdf]

OMTON, Volume *34*

Supplemental information

Mesenchymal stem cell-derived extracellular vesicle therapy in breast cancer: A systematic review and meta-analysis of *in vitro* studies

Nadiar M. Mussin, Kulyash R. Zhilisbayeva, Akmaral Baspakova, Lunara A. Ishimova, Madina A. Kurmanalina, and Amin Tamadon

Table S1. PRISMA 2020 checklist

Section and Topic	Item #	Checklist item	Location in Manuscript
Title	1	Identify the report as a systematic review and/or meta-analysis.	Title page
Abstract	2	Provide a structured summary including background, objectives, data sources, eligibility criteria, participants/interventions, study appraisal, synthesis methods, results, limitations, conclusions, and registration information.	Abstract
Introduction	3	Describe the rationale for the review in the context of existing knowledge.	Introduction, paras 1–2
	4	Provide an explicit statement of the objectives or questions addressed.	Introduction, final paragraph
Methods	5	Specify inclusion and exclusion criteria and how studies were grouped for syntheses.	Materials and Methods → Inclusion/Exclusion Criteria
	6	Specify all information sources (databases, registers, websites, organizations) searched and the date last searched.	Methods → Search Strategy
	7	Present full search strategies for all databases, including any filters and limits used.	Supplemental Table S1
	8	Specify the process for selecting studies (screening, eligibility, inclusion).	Methods → Study Selection / PRISMA Flow Diagram (Fig. 1)
	9	Describe the methods of data collection/extraction from included studies.	Methods → Data Extraction
	10	List and define all variables sought (e.g., outcomes, exposures, predictors).	Methods → Data Extraction Template
	11	Specify methods used to assess risk of bias in included studies.	Methods → Quality Assessment
	12	Specify effect measures used for each outcome (e.g., risk ratio, SMD).	Methods → Statistical Analysis
	13	Describe methods of synthesis and rationale for the choice(s). State how effect estimates were combined and how heterogeneity was assessed.	Methods → Statistical Analysis
	14	Describe any methods used to assess potential reporting bias (e.g., funnel plot, Egger's test).	Results → Meta-analysis / Publication Bias (Fig. S7)
	15	Describe any methods used to assess certainty (confidence) in the body of evidence.	Discussion → Limitations
Results	16	Describe results of search and selection process, ideally using a flow diagram.	Results → Study Selection / Fig. 1

	17	Present characteristics for each study (e.g., study size, MSC source, EV isolation method).	Results → Study Characteristics / Table 1
	18	Present risk-of-bias assessments for each study.	Results → Quality Assessment / Fig. 2
	19	Present results of individual studies and syntheses, including summary statistics and forest plots.	Results → Meta-analysis / Figs 3–5
	20	Describe results of sensitivity analyses, subgroup analyses, and heterogeneity.	Results → Meta-analysis Results / Subgroup and Sensitivity Analyses
	21	Present results of any assessment of publication bias.	Results → Meta-analysis / Fig. S7
Discussion	22	Provide a general interpretation of the results in the context of other evidence.	Discussion → Dualistic Effects and Applied Engineering
	23	Discuss limitations of the evidence and of the review process.	Discussion → Limitations
	24	Discuss implications of the results for practice, policy, and future research.	Discussion → Future Directions
Other information	25	Provide registration information for the review (e.g., PROSPERO ID) or state if not registered.	Not registered – stated in Methods
	26	Indicate sources of financial or non-financial support for the review.	Acknowledgments
	27	Declare any competing interests of the review authors.	Declaration of Interest Statement
	28	Describe availability of data, code, and other materials used in the review.	Data Availability / Supplemental Information

Table S2. Detailed Boolean search strategy for three conceptual domains

Code	Query
#1 (Extracellular Vesicles)	(“Extracellular Vesicles” OR “Extracellular Vesicle” OR “Vesicle, Extracellular” OR “Vesicles, Extracellular” OR “Exovesicles” OR “Exovesicle” OR “Apoptotic Bodies” OR “Apoptotic Body” OR “Bodies, Apoptotic” OR “Body, Apoptotic” OR “Exosomes”)
#2 (Mesenchymal Stem/Stromal Cells)	(“Mesenchymal Stem Cells” OR “Mesenchymal Stem Cell” OR “Stem Cells, Mesenchymal” OR “Stem Cell, Mesenchymal” OR “Mesenchymal Stromal Cells” OR “Mesenchymal Stromal Cell” OR “Stromal Cells, Mesenchymal” OR “Stromal Cell, Mesenchymal” OR “Wharton’s Jelly Cells” OR “Wharton Jelly Cells” OR “Bone Marrow Stromal Cells” OR “Bone Marrow Stromal Cell” OR “Multipotent Bone Marrow Stromal Cells” OR “Mesenchymal Progenitor Cells” OR “Bone Marrow Mesenchymal Stem Cells” OR “Adipose-Derived Mesenchymal Stem Cells” OR “Adipose-Derived Mesenchymal Stromal Cells” OR “Adipose Tissue-Derived Mesenchymal Stem Cells” OR “Adipose Tissue-Derived Mesenchymal Stromal Cells”)
#3 (Breast Cancer/Neoplasms)	(“Breast Neoplasms” OR “Breast Neoplasm” OR “Breast Tumor” OR “Breast Tumors” OR “Breast Cancer” OR “Cancer of the Breast” OR “Breast Carcinoma” OR “Breast Carcinomas” OR “Mammary Cancer” OR “Mammary Cancers” OR “Human Mammary Neoplasms” OR “Human Mammary Carcinoma” OR “Carcinoma, Breast” OR “Carcinomas, Human Mammary”)
#4 (Combined Search)	#1 AND #2 AND #3

Study or Subgroup	Experimental Mean	SD	Total	Control Mean	SD	Total	Weight	Std. Mean Difference IV, Random, 95% CI
**subgroup = MCF7**								
Ababneh et al. 2025	45.80	4.2000	3	100.00	5.8000	3	1.8%	-8.54 [-16.46; -0.62]
Aldiqs et al. 2025	150.00	1.0000	3	100.00	6.0000	3	1.6%	9.28 [0.70; 17.85]
Attar et al. 2025	30.00	5.0000	3	100.00	5.0000	3	1.2%	-11.17 [-21.44; -0.90]
Ebrahimian et al. 2022	2.00	1.0000	3	20.00	5.0000	3	3.9%	-3.98 [-7.94; -0.03]
Felthaus et al. 2024	106.70	11.7000	3	100.00	10.0000	3	5.8%	0.49 [-1.17; 2.15]
Liu et al. 2022	51.70	0.7000	3	100.00	0.7000	3	0.1%	-55.05 [-105.07; -5.04]
Mohd Ali et al. 2020	80.00	4.0000	3	100.00	5.0000	3	4.2%	-3.52 [-7.10; 0.05]
Naseri et al. 2020	70.00	7.0000	3	100.00	6.0000	3	4.1%	-3.67 [-7.37; 0.03]
Ramirez et al. 2024	45.00	8.0000	3	100.00	10.0000	3	3.4%	-4.85 [-9.53; -0.16]
Rezaie et al. 2018	50.00	4.2000	3	100.00	5.0000	3	1.8%	-8.64 [-16.65; -0.63]
**Total (95% CI)**			**30**			**30**	**27.9%**	**-3.51 [-6.30; -0.72]**
Heterogeneity: Tau^2 = 10.6920; Chi^2 = 31.47, df = 9 (P = 0.0002); I^2 = 71.4%								
**subgroup = MDA-MB-231**								
Altanerova et al. 2019	10.00	5.0000	3	100.00	5.0000	3	0.8%	-14.36 [-27.50; -1.22]
Attar et al. 2025	20.00	5.0000	3	100.00	5.0000	3	1.0%	-12.77 [-24.47; -1.07]
Chulpanova et al. (a) 2021	60.00	5.0000	3	90.00	5.0000	3	3.4%	-4.79 [-9.42; -0.16]
Chulpanova et al. (b) 2021	40.00	5.0000	3	90.00	5.0000	3	2.0%	-7.98 [-15.40; -0.56]
Eiro et al. 2024	15.00	10.0000	3	76.00	5.0000	3	2.7%	-6.16 [-11.97; -0.34]
Farhadi et al. 2023	40.90	9.1000	3	100.00	18.2000	3	4.4%	-3.28 [-6.66; 0.10]
Hass et al. 2024	80.00	4.0000	3	100.00	6.0000	3	4.5%	-3.13 [-6.39; 0.13]
Jafarpour et al. 2024	25.00	4.0000	3	100.00	2.0000	3	0.5%	-18.92 [-36.18; -1.67]
Kaan et al. 2023	45.00	1.0000	3	100.00	1.0000	3	0.1%	-43.88 [-83.76; -4.01]
Khazaei-Poul et al. 2021	52.20	3.5000	3	100.00	4.4000	3	1.5%	-9.59 [-18.45; -0.74]
Mohd Ali et al. 2021	85.00	6.0000	3	100.00	6.0000	3	5.2%	-1.99 [-4.41; 0.42]
Pakravan et al. 2017	45.00	1.0000	3	95.00	1.0000	3	0.1%	-39.89 [-76.15; -3.64]
Patel et al. 2021	5.00	1.0000	3	95.00	3.0000	3	0.2%	-32.11 [-61.32; -2.91]
Shojaei et al. 2021	57.10	8.6000	3	100.00	11.4000	3	4.3%	-3.39 [-6.86; 0.08]
Shojaei et al. 2023	66.70	1.4000	3	100.00	1.4000	3	0.5%	-18.98 [-36.28; -1.67]
Vakhshiteh et al. 2021	90.00	5.0000	3	100.00	2.4000	3	5.2%	-2.03 [-4.48; 0.41]
Xu et al. 2024	16.70	1.7000	3	100.00	1.7000	3	0.1%	-39.10 [-74.63; -3.56]
Zhang et al. 2024	20.00	4.0000	3	35.00	1.0000	3	3.8%	-4.11 [-8.16; -0.05]
**Total (95% CI)**			**54**			**54**	**40.2%**	**-3.83 [-5.05; -2.61]**
Heterogeneity: Tau^2 = 0.4447; Chi^2 = 33.81, df = 17 (P = 0.0089); I^2 = 49.7%								
**subgroup = 4T1**								
Egea et al. 2022	68.50	1.0000	3	100.00	1.0000	3	0.3%	-25.13 [-48.01; -2.26]
Hosseini et al. 2024	50.00	6.0000	3	97.00	2.0000	3	1.8%	-8.39 [-16.17; -0.61]
Lee et al. 2013	78.10	6.3000	3	100.00	6.3000	3	4.7%	-2.77 [-5.76; 0.21]
Mirabdollahi et al. 2020	10.00	3.0000	3	100.00	2.0000	3	0.2%	-28.17 [-53.79; -2.54]
Naseri et al. 2018	76.00	4.0000	3	100.00	4.0000	3	3.4%	-4.79 [-9.42; -0.16]
Xu et al. 2024	21.40	1.4000	3	100.00	1.4000	3	0.1%	-44.80 [-85.50; -4.09]
**Total (95% CI)**			**18**			**18**	**10.6%**	**-7.39 [-12.71; -2.06]**
Heterogeneity: Tau^2 = 17.9472; Chi^2 = 12.42, df = 5 (P = 0.0295); I^2 = 59.7%								
**subgroup = T47D**								
Farouk et al. 2024	5.00	1.0000	3	90.00	2.0000	3	0.1%	-42.89 [-81.87; -3.92]
**subgroup = BT-474**								
Gomari et al. 2018	80.00	7.0000	3	100.00	1.0000	3	4.4%	-3.19 [-6.50; 0.12]
Jafarpour et al. 2024	30.00	6.0000	3	100.00	2.0000	3	1.0%	-12.49 [-23.94; -1.04]
**Total (95% CI)**			**6**			**6**	**5.4%**	**-6.16 [-14.65; 2.34]**
Heterogeneity: Tau^2 = 24.7246; Chi^2 = 2.34, df = 1 (P = 0.1263); I^2 = 57.2%								
**subgroup = TUBO**								
Gomari et al. 2019	5.00	1.0000	3	30.00	1.0000	3	0.4%	-19.95 [-38.13; -1.77]
Naseri et al. 2018	81.80	4.6000	3	100.00	4.5000	3	4.4%	-3.19 [-6.50; 0.12]
**Total (95% CI)**			**6**			**6**	**4.9%**	**-9.09 [-24.77; 6.60]**
Heterogeneity: Tau^2 = 95.9247; Chi^2 = 3.16, df = 1 (P = 0.0756); I^2 = 68.3%								
**subgroup = MDA-hyb1**								
Melzer et al. 2019	10.00	2.0000	3	100.00	5.0000	3	0.5%	-18.86 [-36.06; -1.66]
Melzer et al. 2020	15.00	2.0000	3	100.00	5.0000	3	0.5%	-17.81 [-34.06; -1.56]
**Total (95% CI)**			**6**			**6**	**1.0%**	**-18.30 [-30.12; -6.49]**
Heterogeneity: Tau^2 = 0; Chi^2 = 0.01, df = 1 (P = 0.9308); I^2 = 0%								
**subgroup = MCF6**								
Mirabdollahi et al. 2020	20.00	3.0000	3	100.00	2.0000	3	0.3%	-25.04 [-47.82; -2.25]
**subgroup = BCSC**								
Naseri et al. 2020	45.00	6.0000	3	98.00	5.0000	3	2.1%	-7.66 [-14.79; -0.52]
**subgroup = MCF10**								
Naseri et al. 2020	94.00	7.0000	3	100.00	5.0000	3	5.7%	-0.79 [-2.54; 0.97]
**subgroup = Not stated**								
Sandiford et al. 2021	64.70	2.9000	4	100.00	2.3000	4	1.8%	-11.72 [-19.67; -3.76]
**Total (95% CI)**			**136**			**136**	**100.0%**	**-4.79 [-6.03; -3.54]**
**Prediction interval**								**[-9.97; 0.40]**
Heterogeneity: Tau^2 = 6.2154; Chi^2 = 117.42, df = 44 (P < 0.0001); I^2 = 62.5%								
Test for overall effect: Z = -7.54 (P < 0.0001)								
Test for subgroup differences: Chi^2 = 31.16, df = 10 (P = 0.0006)								

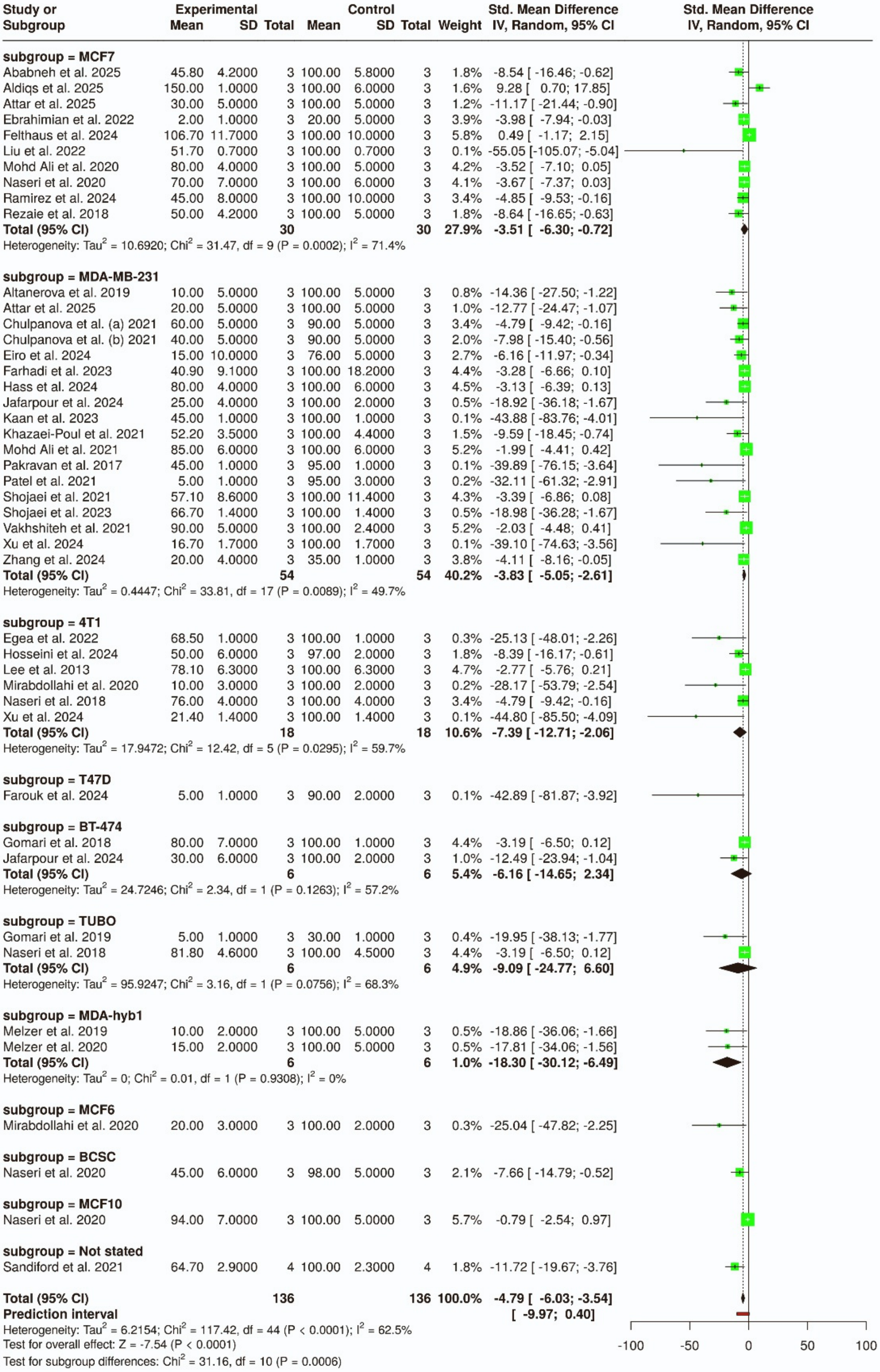

Figure S1. Forest plot of the effect of MSC-derived EVs on breast cancer cell viability, stratified by breast cancer cell line.

Study or Subgroup	Experimental Mean	SD	Total	Control Mean	SD	Total	Weight	Std. Mean Difference IV, Random, 95% CI
**subgroup = MCF6**								
Ababneh et al. 2025	10.00	1.0000	3	10.00	1.0000	3	5.7%	0.00 [-1.60; 1.60]
**subgroup = MCF7**								
Aldiqs et al. 2025	40.00	2.5000	3	30.00	2.5000	3	4.6%	3.19 [-0.12; 6.50]
Attar et al. 2025	90.00	5.0000	3	10.00	2.0000	3	0.7%	16.76 [1.46; 32.07]
Jia et al. 2020	38.00	3.0000	3	22.00	4.0000	3	4.4%	3.61 [-0.04; 7.26]
Liu et al. 2022	25.00	3.0000	3	5.00	1.2000	3	2.6%	6.98 [0.44; 13.52]
Naseri et al. 2020	3.00	1.5000	3	2.00	1.5000	3	5.7%	0.53 [-1.14; 2.20]
Wang et al. 2021	18.00	1.0000	3	6.00	1.0000	3	1.8%	9.57 [0.74; 18.41]
Yang et al. 2022	20.00	1.5000	3	3.00	0.5000	3	1.3%	12.13 [1.00; 23.26]
**Total (95% CI)**			**21**			**21**	**21.0%**	**4.61 [1.53; 7.69]**
Heterogeneity: $Tau^2 = 8.4794$; $Chi^2 = 15.99$, df = 6 (P = 0.0138); $I^2 = 62.5\%$								
**subgroup = MDA-MB-231**								
Altanerova et al. 2019	50.00	8.0000	3	10.00	3.0000	3	3.4%	5.28 [0.23; 10.34]
Attar et al. 2025	85.00	5.0000	3	5.00	1.0000	3	0.7%	17.70 [1.55; 33.86]
Chulpanova et al. 2023	60.00	5.0000	3	20.00	5.0000	3	2.9%	6.38 [0.37; 12.40]
Chulpanova et al. NA	50.00	5.0000	4	20.00	5.0000	4	4.3%	5.21 [1.46; 8.96]
Chulpanova et al. NA	20.00	5.0000	4	10.00	5.0000	4	5.6%	1.74 [-0.07; 3.55]
Eiro et al. 2024	33.00	3.0000	3	2.00	1.0000	3	1.5%	11.06 [0.89; 21.23]
Farhadi et al. 2023	18.00	7.0000	3	5.00	2.0000	3	5.2%	2.01 [-0.42; 4.45]
Jafarpour et al. 2024	78.00	4.0000	3	10.00	2.0000	3	0.7%	17.16 [1.50; 32.82]
Kaan et al. 2023	4.50	0.0000	3	1.00	0.0000	3	0.0%	
Khazaei-Poul et al. 2021	18.00	0.8000	3	13.00	0.8000	3	3.6%	4.99 [0.18; 9.79]
Sandiford et al. 2021	4.00	1.0000	4	13.00	2.0000	4	4.4%	-4.94 [-8.53; -1.36]
Shojaei et al. 2021	16.00	0.8000	3	13.00	0.8000	3	4.7%	2.99 [-0.16; 6.15]
Shojaei et al. 2023	15.00	1.5000	3	9.00	1.0000	3	4.3%	3.76 [-0.01; 7.52]
Vakhshiteh et al. 2021	24.00	3.0000	3	1.00	0.5000	3	2.1%	8.53 [0.62; 16.44]
Wang et al. 2021	18.00	1.0000	3	6.00	1.0000	3	1.8%	9.57 [0.74; 18.41]
Yang et al. 2022	25.00	2.0000	3	3.00	0.5000	3	1.3%	12.04 [0.99; 23.09]
Zhang et al. 2022	8.00	0.6000	3	3.80	0.4000	3	2.8%	6.57 [0.39; 12.75]
**Total (95% CI)**			**54**			**54**	**49.1%**	**4.57 [2.38; 6.76]**
Heterogeneity: $Tau^2 = 10.9704$; $Chi^2 = 42.01$, df = 15 (P = 0.0002); $I^2 = 64.3\%$								
**subgroup = TNBC**								
Ding et al. 2023	40.00	5.0000	3	5.00	2.0000	3	2.5%	7.33 [0.49; 14.18]
**subgroup = 4T1**								
Hosseini et al. 2024	67.00	3.0000	3	4.00	1.0000	3	0.4%	22.48 [2.01; 42.95]
Naseri et al. 2018	6.00	0.1000	3	0.00	0.0000	3	0.0%	
**subgroup = BT-474**								
Jafarpour et al. 2024	82.00	4.0000	3	20.00	3.0000	3	1.0%	13.99 [1.19; 26.80]
**subgroup = MDA-MB-232**								
Jia et al. 2020	36.00	4.0000	3	18.00	4.0000	3	4.4%	3.59 [-0.04; 7.22]
**subgroup = MDA-MB-468**								
Meng et al. 2023	40.00	4.0000	3	1.00	0.5000	3	1.5%	10.92 [0.88; 20.96]
**subgroup = TUBO**								
Naseri et al. 2018	6.00	0.1000	3	0.00	0.0000	3	0.0%	
**subgroup = BCSC**								
Naseri et al. 2020	7.00	1.0000	3	1.00	1.0000	3	3.7%	4.79 [0.16; 9.42]
**subgroup = MCF10**								
Naseri et al. 2020	1.00	1.0000	3	1.00	1.0000	3	5.7%	0.00 [-1.60; 1.60]
**subgroup = SK-BR-3**								
Zhang et al. 2022	6.30	0.4000	3	5.00	0.4000	3	4.9%	2.59 [-0.25; 5.44]
**Total (95% CI)**			**108**			**108**	**100.0%**	**4.16 [2.75; 5.56]**
**Prediction interval**								**[-1.91; 10.23]**

Heterogeneity: $Tau^2 = 8.3396$; $Chi^2 = 87.55$, df = 31 (P < 0.0001); $I^2 = 64.6\%$
Test for overall effect: Z = 5.80 (P < 0.0001)
Test for subgroup differences: $Chi^2 = 34.90$, df = 10 (P = 0.0001)

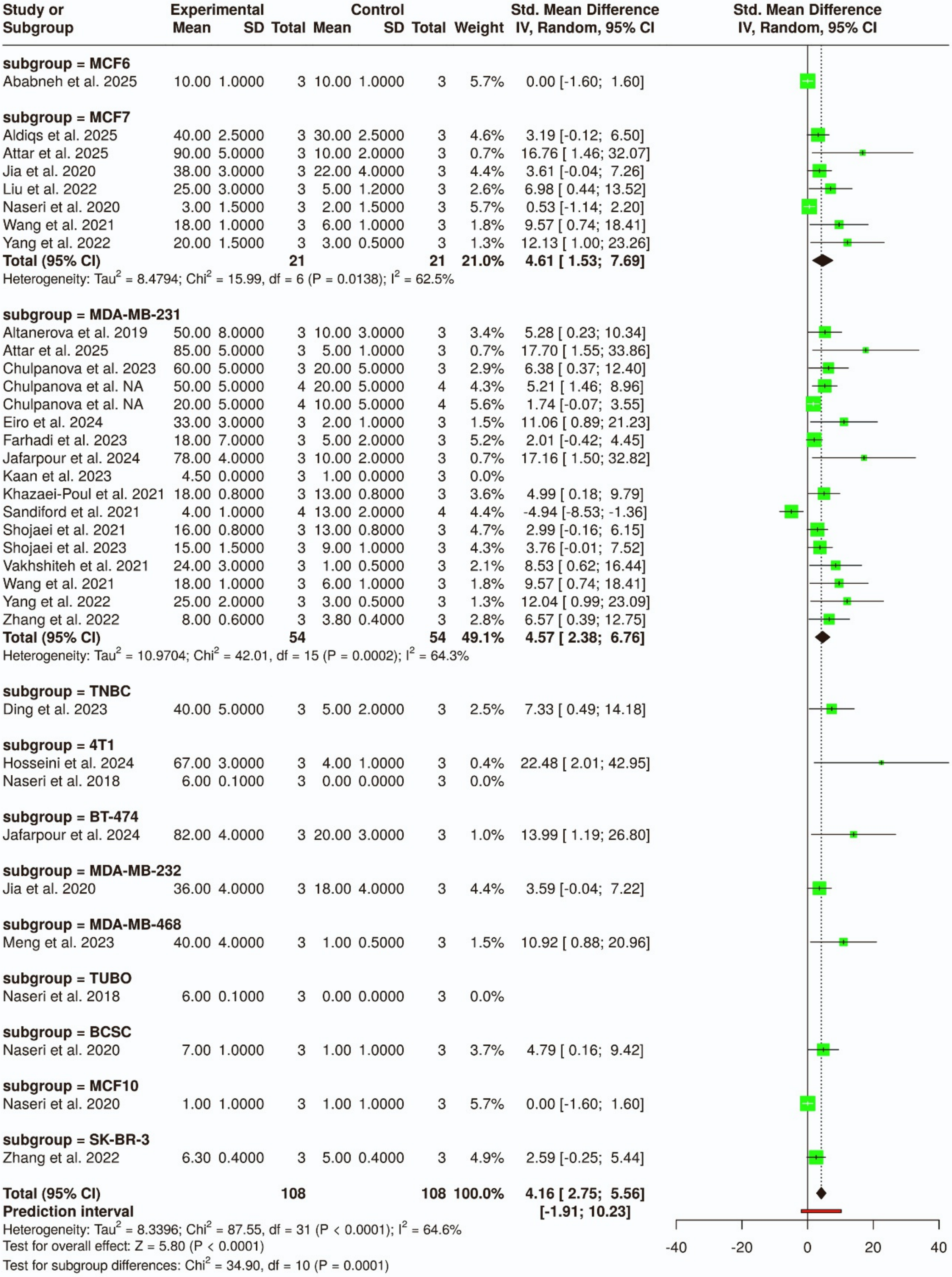

Figure S2. Forest plot of standardized mean differences in breast cancer cell apoptosis following MSC-EV treatment, stratified by breast cancer cell line.

Study or Subgroup	Experimental Mean	Experimental SD	Experimental Total	Control Mean	Control SD	Control Total	Weight	Std. Mean Difference IV, Random, 95% CI
**subgroup = MCF7**								
Ababneh et al. 2025	80.00	10.0000	3	100.00	10.0000	3	7.5%	-1.60 [-3.75; 0.56]
Aldiqs et al. 2025	125.00	13.0000	3	100.00	13.0000	3	7.5%	1.53 [-0.59; 3.66]
Du et al. 2021	40.00	5.0000	3	80.00	5.0000	3	4.4%	-6.38 [-12.40; -0.37]
Liu et al. 2022	20.00	2.0000	3	60.00	5.0000	3	3.4%	-8.38 [-16.16; -0.61]
Mohd Ali et al. 2020	40.00	4.0000	3	80.00	5.0000	3	4.0%	-7.05 [-13.65; -0.45]
Ramirez et al. 2024	5.00	1.0000	3	30.00	1.0000	3	0.9%	-19.95 [-38.13; -1.77]
Yang et al. 2022	48.00	3.0000	3	75.00	4.0000	3	4.6%	-6.09 [-11.85; -0.33]
**Total (95% CI)**			**21**			**21**	**32.4%**	**-4.32 [-7.88; -0.76]**
Heterogeneity: $Tau^2 = 13.8955$; $Chi^2 = 22.47$, df = 6 (P = 0.0010); $I^2 = 73.3\%$								
**subgroup = MDA-MB-231**								
Chang et al. 2022	80.00	5.0000	3	70.00	5.0000	3	7.5%	1.60 [-0.56; 3.75]
Du et al. 2021	50.00	5.0000	3	200.00	10.0000	3	1.5%	-15.14 [-28.98; -1.30]
Eiro et al. 2024	28.13	15.6300	3	100.00	18.7500	3	6.5%	-3.32 [-6.74; 0.09]
Farhadi et al. 2023	34.60	5.8000	3	100.00	5.8000	3	3.1%	-9.00 [-17.32; -0.67]
Khazaei-Poul et al. 2021	49.30	0.0000	3	100.00	0.0000	3	0.0%	
Mohd Ali et al. 2021	45.00	4.0000	3	70.00	5.0000	3	5.8%	-4.41 [-8.71; -0.10]
Pakravan et al. 2017	30.40	5.2000	3	100.00	8.7000	3	3.7%	-7.75 [-14.96; -0.53]
Shojaei et al. 2021	60.00	1.0000	3	85.00	1.0000	3	0.9%	-19.95 [-38.13; -1.77]
Shojaei et al. 2023	70.00	3.0000	3	88.00	2.0000	3	4.9%	-5.63 [-10.99; -0.27]
Vakhshiteh et al. 2021	70.00	3.1000	3	98.00	2.4000	3	3.5%	-8.06 [-15.55; -0.57]
Yang et al. 2022	50.00	5.0000	3	70.00	5.0000	3	6.6%	-3.19 [-6.50; 0.12]
Zhang et al. 2022	27.30	4.5000	3	100.00	4.5000	3	1.9%	-12.89 [-24.70; -1.08]
**Total (95% CI)**			**36**			**36**	**45.9%**	**-5.17 [-7.97; -2.37]**
Heterogeneity: $Tau^2 = 11.5457$; $Chi^2 = 32.47$, df = 10 (P = 0.0003); $I^2 = 69.2\%$								
**subgroup = 4T1**								
Egea et al. 2021	25.00	5.0000	3	100.00	10.0000	3	3.8%	-7.57 [-14.63; -0.51]
Hosseini et al. 2024	37.50	4.0000	3	100.00	1.0000	3	1.2%	-17.10 [-32.72; -1.49]
Lee et al. 2013	35.00	5.0000	3	100.00	5.0000	3	2.6%	-10.37 [-19.93; -0.82]
Zhou et al. 2024	2.00	1.0000	3	9.00	2.0000	3	6.4%	-3.53 [-7.12; 0.05]
**Total (95% CI)**			**12**			**12**	**13.9%**	**-6.82 [-11.41; -2.23]**
Heterogeneity: $Tau^2 = 7.9198$; $Chi^2 = 4.61$, df = 3 (P = 0.2030); $I^2 = 34.9\%$								
**subgroup = SK-BR-3**								
Zhang et al. 2022	95.00	5.0000	3	100.00	5.0000	3	7.8%	-0.80 [-2.55; 0.96]
**Total (95% CI)**			**72**			**72**	**100.0%**	**-4.70 [-6.54; -2.85]**
**Prediction interval**								**[-11.71; 2.32]**
Heterogeneity: $Tau^2 = 10.5687$; $Chi^2 = 67.59$, df = 22 (P < 0.0001); $I^2 = 67.4\%$								
Test for overall effect: Z = -4.99 (P < 0.0001)								
Test for subgroup differences: $Chi^2 = 11.38$, df = 3 (P = 0.0098)								

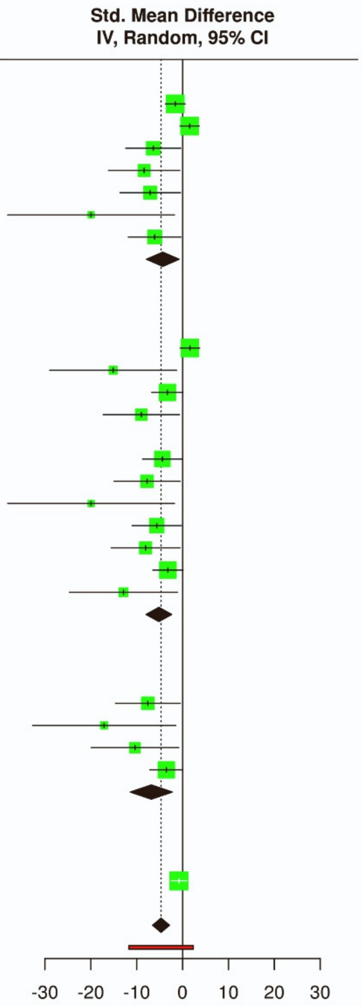

Figure S3. Forest plot of standardized mean differences in breast cancer cell migration following MSC-EV treatment, stratified by breast cancer cell line.

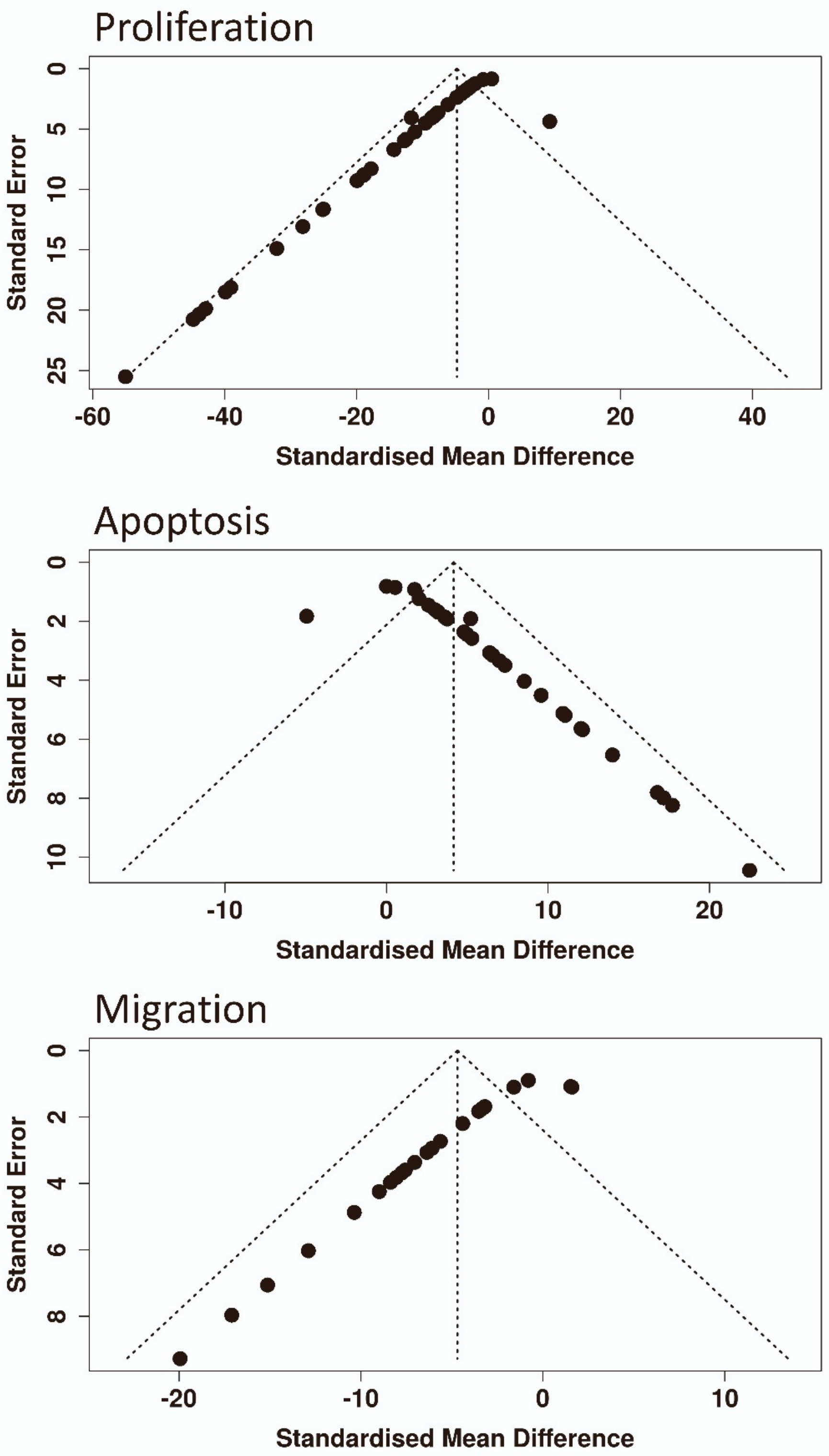

Figure S4. Funnel plots for assessment of publication bias in MSC-EV meta-analyses. Funnel plots and Egger's tests revealed no significant asymmetry for any outcome ($p > 0.05$), suggesting limited publication bias across the analyzed studies. Plots are shown for (A) proliferation/viability, (B) apoptosis, and (C) migration outcomes.

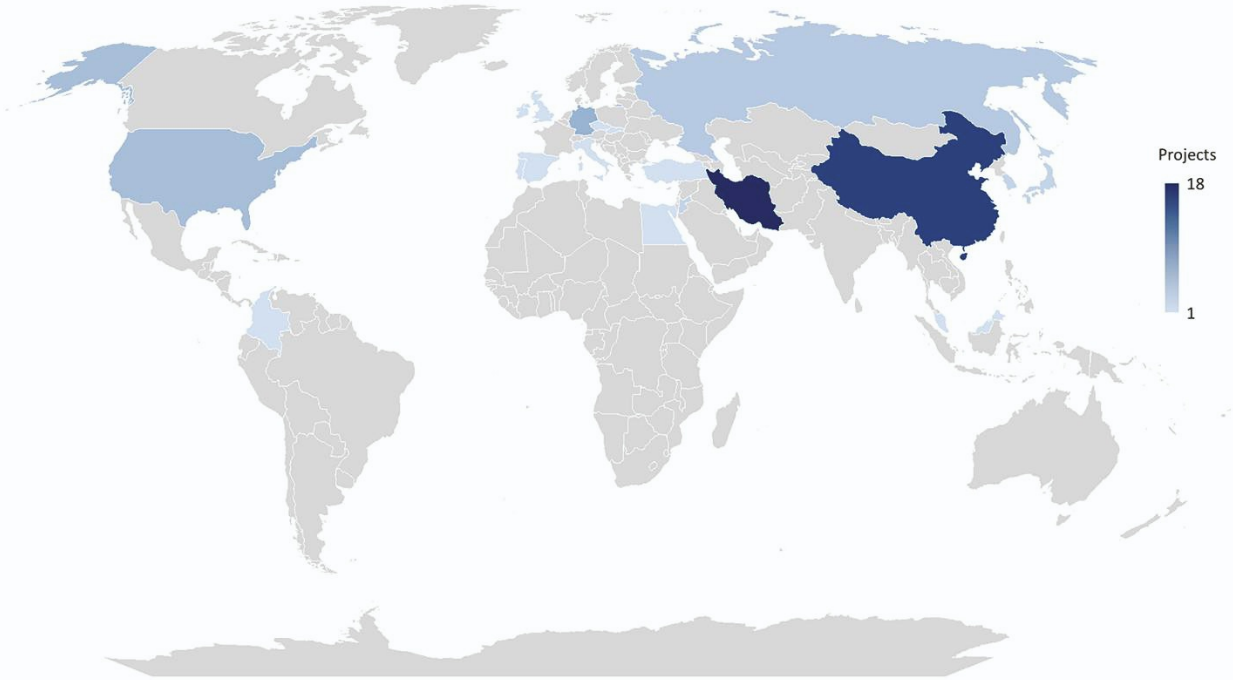

Figure S5. Geographic distribution of MSC-EV studies in breast cancer by country (number of studies per country).
