# Supplementary material for: Mesenchymal stem cell-derived extracellular vesicle therapy in breast cancer: A systematic review and meta-analysis of in vitro studies
Source: Mol Ther Oncol. 2025 Dec 9;34(1):201107. doi: 10.1016/j.omton.2025.201107 (PMC12775850; doi:10.1016/j.omton.2025.201107)
Supplement: Document S2. Article plus supplemental information [file mmc2.pdf]

Molecular Therapy
Oncology
Review

Mesenchymal stem cell-derived extracellular vesicle therapy in breast cancer: A systematic review and meta-analysis of *in vitro* studies

Nadiar M. Mussin,[1] Kulyash R. Zhilisbayeva,[2] Akmaral Baspakova,[3] Lunara A. Ishimova,[4] Madina A. Kurmanalina,[5] and Amin Tamadon[6]

[1]Department of General Surgery No. 2, West Kazakhstan Marat Ospanov Medical University, Aktobe, Kazakhstan; [2]Department of Languages, West Kazakhstan Marat Ospanov Medical University, Aktobe, Kazakhstan; [3]Department of Epidemiology, West Kazakhstan Marat Ospanov Medical University, Aktobe, Kazakhstan; [4]Department of Public Health and Health Care, West Kazakhstan Marat Ospanov Medical University, Aktobe, Kazakhstan; [5]Department of Dental Disciplines and Maxillofacial Surgery, West Kazakhstan Marat Ospanov Medical University, Aktobe, Kazakhstan; [6]Department of Natural Sciences, West Kazakhstan Marat Ospanov Medical University, Aktobe, Kazakhstan

Mesenchymal stem/stromal cell-derived extracellular vesicles (MSC-EVs) have emerged as promising cell-free therapeutics for breast cancer due to their innate tumor tropism and molecular delivery capacity. This systematic review and meta-analysis evaluated the *in vitro* therapeutic potential and safety of MSC-EVs. A comprehensive search up to July 2025 identified 58 eligible studies. Quantitative data were extracted on cell viability, apoptosis, and migration, along with EV source, cargo, and engineering strategy. Random-effects meta-analyses showed that MSC-EV treatment significantly reduced cancer cell viability (standardized mean difference [SMD] = −4.79), inhibited migration (SMD = −4.70), and increased apoptosis (SMD = +4.16). Effects were consistent across major cell lines (MCF-7, MDA-MB-231, and 4T1) and MSC sources (bone marrow, adipose, and umbilical cord), despite moderate heterogeneity (I^2 = 50%–70%). Notably, unmodified bone marrow MSC-EVs carrying miR-23b were associated with dormancy induction *in vivo*, whereas engineered EVs loaded with therapeutic miRNAs or drugs and modified with targeting ligands demonstrated improved specificity and efficacy. Precision engineering of MSC-EVs can enhance antitumor activity but requires stringent cargo control to avoid dormancy risks. Only *in vitro* data were quantitatively analyzed, while *in vivo* findings were discussed for mechanistic context, providing a methodological foundation for future translational research.

INTRODUCTION

Breast cancer remains the most frequently diagnosed malignancy in women and a leading cause of cancer deaths worldwide, with nearly 2.3 million new cases and 685,000 fatalities projected in 2025.[1] Despite advances in surgery, chemotherapy, targeted therapy, and immunotherapy, therapy resistance and tumor recurrence—especially in cases involving dormant micrometastases—continue to challenge long-term survival.[2] Emerging as a promising cell-free alternative—extracellular vesicles (EVs), specifically small EVs (60–200 nm) and phospholipid-bound nanovesicles carrying proteins, lipids, mRNAs, and miRNAs—have gained attention for their roles in cancer biology and therapeutic potential.[3]

Mesenchymal stem/stromal cell-derived EVs (MSC-EVs) possess unique properties: low immunogenicity, innate tumor tropism, and capacity for molecular engineering. These traits suggest they could serve as effective therapeutic delivery vehicles.[4] Indeed, preclinical studies have reported that MSC-EVs loaded with miR-16, paclitaxel, and miRNA inhibitors could inhibit breast cancer cell proliferation, angiogenesis, migration, and chemoresistance.[5] Recent reviews have outlined the dual role of MSC-EVs in tumor progression and therapy, underscoring the need for systematic synthesis of *in vitro* data.[4] Moreover, surface modifications—such as cRGD and LAMP2b-DRARPin—enhance tumor targeting while minimizing off-target effects.[6]

However, MSC-EVs exhibit a dualistic behavior.[7] Certain unmodified bone marrow MSC-EVs have been shown to promote dormancy in metastatic breast cancer cells through the transfer of miR-23b.[4] Additionally, stromal EVs carrying miR-21-5p, and adipocyte-EVs activating Hippo signaling, have been implicated in enhanced proliferation, chemoresistance, and tumor progression, highlighting safety concerns for clinical translation.[8]

Given these divergent outcomes, a nuanced evaluation of MSC-EV efficacy and safety is critical. Here, we conducted a systematic review and meta-analysis, guided by PRISMA standards and encompassing *in vitro* studies and *in vivo* data, to assess the therapeutic impact, heterogeneity, and mechanistic underpinnings of MSC-EV interventions in breast cancer. We aimed to clarify how the EV source, cargo,

https://doi.org/10.1016/j.omton.2025.201107.

Correspondence: Amin Tamadon, Department of Natural Sciences, West Kazakhstan Marat Ospanov Medical University, Aktobe, Kazakhstan.
E-mail: amintamaddon@yahoo.com

© 2025 The Author(s).
Published by Elsevier Inc. on behalf of The American Society of Gene and Cell Therapy.
This is an open access article under the CC BY license (http://creativecommons.org/licenses/by/4.0/).

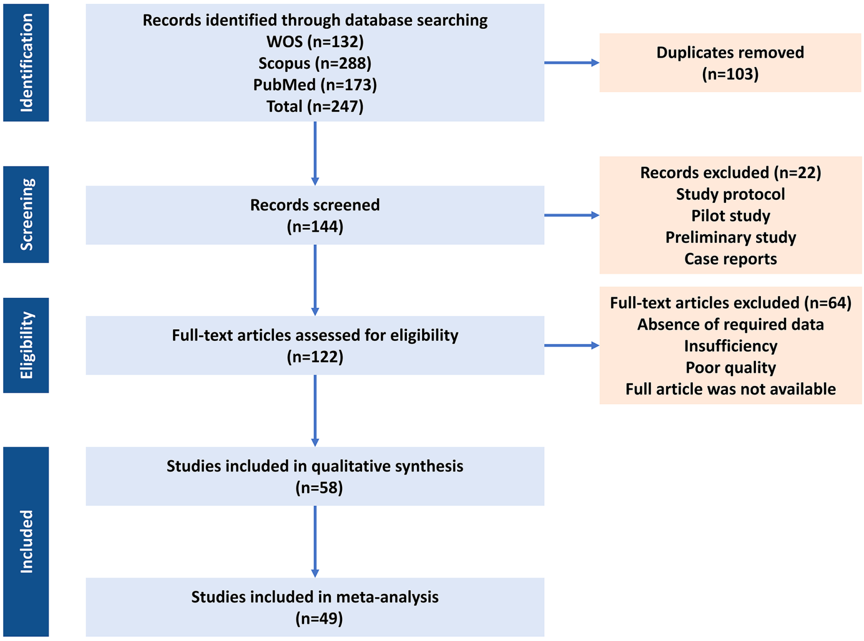

Figure 1. Study selection flow diagram
PRISMA 2020 flow diagram summarizing the identification, screening, and inclusion of studies in the systematic review of *in vitro* investigations on mesenchymal stromal/stem cell-derived extracellular vesicle (MSC-EV) therapy in breast cancer.

and engineering strategies influence efficacy versus dormancy risk, while outlining translational considerations to optimize MSC-EV platforms for safe and effective clinical applications. While *in vivo* data remain limited, mapping *in vitro* evidence offers essential mechanistic insights that underpin the rational design of translational MSC-EV therapeutics.

RESULTS

Study selection

The study selection process followed the PRISMA (Preferred Reporting Items for Systematic Reviews and Meta-Analyses) 2020 guidelines, based on which 58 of the 247 retrieved records were considered to meet all inclusion criteria. The PRISMA flow diagram (Figure 1) summarizes the screening and exclusion details. Studies included were original *in vitro* experiments using MSC-EVs in breast cancer models and reporting outcomes on proliferation, apoptosis, migration, invasion, or molecular mechanisms. Of the 58 studies meeting the qualitative inclusion criteria, nine lacked extractable quantitative data or had used non-comparable assay formats and were, therefore, excluded from the meta-analysis.

Study characteristics

The 58 studies evaluated MSC-EVs from various sources (bone marrow, adipose, umbilical cord, Wharton's jelly, placenta, uterine, dental pulp, and menstrual). Isolation techniques (ultracentrifugation, kits, or chromatography) and characterization by TEM, DLS, and CD63/CD81/CD9 western blotting were common. Primary endpoints included proliferation, apoptosis, migration, and invasion.

Many studies explored both natural (endogenous) and loaded EV cargo, including miRNAs (e.g., miR-21-5p, miR-125b, and miR-34a), chemotherapeutic agents (e.g., paclitaxel and doxorubicin), lncRNAs, and gene therapy constructs (e.g., suicide genes). EV loading was achieved via methods such as electroporation, chemical incubation, or genetic modification of donor MSCs. Primary outcomes included assessments of cell proliferation, apoptosis, migration, and invasion, with many studies using assays such as MTT, Annexin V/PI, transwell migration, and wound healing. Several studies also evaluated molecular pathways and *in vivo* tumor suppression in xenograft models.

Table 1 presents a comprehensive overview of the study characteristics, including MSC source, EV isolation and characterization methods, cargo content, breast cancer cell line(s), intervention protocols, and primary/secondary outcomes. Table 2 lists the geographic and funding distribution of the included *in vitro* studies.

Quality assessment

A total of 58 *in vitro* studies investigating the effects of MSC-EVs on breast cancer were assessed for methodological quality by using a modified version of the SYRCLE's Risk of Bias tool. Although originally designed for animal studies, the tool was adapted to suit the context of *in vitro* research, evaluating six domains: selection bias, performance bias, detection bias, attrition bias, reporting bias, and other potential sources of bias, such as EV characterization and funding transparency. The findings of this assessment are summarized in Figure 2.

Most studies (52 out of 58) were rated as low risk across all domains. These studies demonstrated clear and appropriate selection of MSC sources and breast cancer cell lines, with adequate justification and detailed methodological descriptions. They maintained standardized experimental conditions, including consistent culture environments, EV dosing, and proper use of control groups. Validated and reproducible assays were frequently employed for outcome measurements, and most studies reported their results comprehensively, including nonsignificant findings, thereby minimizing the risk of selective outcome reporting.

However, six studies were found to have a high risk of bias in at least one domain. Studies by Altanerova et al.[11] and Meng et al.[43] exhibited high risk in both selection bias and other bias due to insufficient description or justification of cell and MSC sources and poor

Table 1. Characteristics of *in vitro* studies on mesenchymal stem cell-derived extracellular vesicle therapy in breast cancer

Author(s), year (reference)	MSC source	EV isolation method	EV characterization	Loaded EV	Breast cancer cell line(s)	Intervention details	Primary outcomes	Secondary outcomes	Key findings	Limitations
Ababneh et al., 2025[9]	BMSCs	ultracentrifugation	flow cytometry (CD9, CD81, CD63); TEM; DLS (size range 32–220 nm)	none	MCF7	50 μg/mL EVs; time points: 24 h, 48 h; MTT assay, apoptosis assay, senescence staining, and wound healing assay	significant proliferation inhibition (MTT) at 24 h	migration (MCF7 increased at 20 h, reversed at 47 h); no significant apoptosis	iMSC-EVs induced longer-lasting proliferation suppression in A549; BMSC-EVs had transient effects in MCF7; senescence increased only in A549	no *in vivo* studies; transient effect in MCF7; MTT limitations; no mechanistic cargo profiling
Aldiqs et al., 2025[10]	ATMSCs	ultracentrifugation	flow cytometry (CD9, CD81, and CD63); Dil-O uptake assay	none	MCF7	50 μg/mL EVs; 24 h, 48 h, 72 h; MTT, Annexin V/PI, SA-β-Gal staining, wound healing assay, and qRT-PCR (BAX and BCL-2)	↓ viability (MTT), ↑ apoptosis (Annexin V/PI)	↑ migration in MCF7 (time-dependent); differential gene expression (↓BAX in MCF7 with ADMSC-Exos; ↓BCL-2 in MCF7 with both Exos)	antitumor effects on proliferation and apoptosis; ADMSC-Exos promoted migration in MCF7	no *in vivo* validation; migration increased despite apoptosis; unclear mechanistic pathway
Altanerova et al., 2019[11]	DPSCs<br>MenSCs<br>BMSCs<br>ATMSCs<br>UCMSCs	ultracentrifugation; size-exclusion chromatography	Nanosight, TEM, PCR/qRT-PCR for suicide gene mRNA, and BCA assay	yCD::UPRT mRNA	MDA-MB-231	suicide gene-transduced MSCs releasing EVs; treated with 5-FC	tumor cell death by intracellular 5-FC conversion	migration effects, miRNA profiling	EVs act via suicide gene mRNA; cell-specific uptake and tumor selectivity	no direct *in vivo* EV-only trials
Attar et al., 2025[12]	ATMSCs	EV Isolation Kit (Anacell)	SEM, DLS, ELISA, western blot (Calnexin), and flow cytometry	doxorubicin (Exo-Dox)	MDA-MB-231<br>MCF-7	Exo-Dox versus free Dox, MTT, apoptosis, migration, and *in vivo* tumor assays	increased apoptosis, reduced migration, tumor suppression	downregulation of H19, UCA1; upregulation of TP53	Exo-Dox more effective than Dox *in vitro* and *in vivo*, especially with CAFs	no long-term toxicity/survival study; small *in vivo* sample size
Bliss et al., 2016[13]	BMSCs	differential centrifugation; EV Isolation Kit	TEM, western blot (CD63, CD81), nanoparticle tracking, and flow cytometry	none	MDA-MB-231<br>T47D	primed/naive MSC-EVs, *in vitro* cycling analysis; *in vivo* antagomiR-222/223 therapy in mice	induction of dormancy/quiescence, miRNA profiling (miR-222/223), and tumor suppression	drug sensitivity, P-gp reduction, Ki67, CD45 IHC, human PPIB PCR	primed MSC-Exos induced dormancy in BCCs; antagomiRs sensitized tumors to carboplatin	focus on miR-222/223; no exosomal loading validation
Casson et al., 2018[14]	BMSCs	ultracentrifugation	TEM, DLS, AChE assay, and ImageJ analysis	none	MCF7	2D and 3D culture, spheroid models, adhesion, proliferation, migration, Ki67, and ALDH1A1 assays	↓ proliferation; ↓ ALDH1A1; ↓ Ki67; ↑adhesion	migration directionality, spheroid compactness, EMT markers	MSC-EVs promoted dormancy via reduced proliferation and enhanced adhesion in MCF7 cells	no *in vivo* data, limited mechanistic cargo analysis
Chang et al., 2022[15]	WJMSCs	ultracentrifugation	TEM, western blot (CD63, TSG101), particle size analyzer, and PKH26 labeling	miR-125b (endogenous)	MDA-MB-231	WJ-EV internalized into BCCs under hypoxia; *in vitro* and *in vivo* assays; miR-125b overexpression/inhibition	↓ proliferation; ↓ migration; ↓ EMT markers; ↓ HIF1α expression; ↓ angiogenesis	↓ tumor growth and metastasis *in vivo*; ↓ CAF induction; transformed EV effects (wBCC-EV)	WJ-EV-transformed BCC (wBCC) reduced tumorigenesis, altered miRNA profile (↑ miR-125b), and produced EVs with anti-tumor properties	comparison limited to AT-EV versus WJ-EV; long-term stability of transformation unclear
Chulpanova et al., 2021a[16]	ATMSCs	CIMVs	flow cytometry, SEM, western blot, qPCR, and immunofluorescence	TRAIL, PTEN, IFN-OI1	MDA-MB-231	50 μg/mL CIMVs; assays: viability, apoptosis, immune co-culture, and RTCA xCelligence	↑ apoptosis in MDA-MB-231 (late apoptosis & necrosis)	↑ activated T-killers ($CD8^{+}$); ↓ NK cells; unchanged Th1/Th2/Th17/Tregs	CIMVs induced apoptosis in TRAIL-sensitive tumors (not HCT-116) and stimulated $CD8^{+}$ T-cells	no *in vivo* study, moderate protein expression, no additive PBMC cytotoxicity

(*Continued on next page*)

Table 1. *Continued*

Author(s), year (reference)	MSC source	EV isolation method	EV characterization	Loaded EV	Breast cancer cell line(s)	Intervention details	Primary outcomes	Secondary outcomes	Key findings	Limitations
Chulpanova et al., 2021b[17]	ATMSCs	CIMVs	flow cytometry, SEM, qPCR, western blot, ELISA, and immunofluorescence	IL2	MDA-MB-231 MDA-MB-436	50 μg/mL CIMVs-IL2 incubated with PBMCs; cytotoxicity assessed on TNBC lines	↑ $CD8^{+}$ T-killer activation and ↑ cytotoxicity against MDA-MB-231 and MDA-MB-436	no ↑ in Tregs; ↓ proliferation suppression vs. hADSCs; ↓ cytokine storm in mice	CIMVs-IL2 were more immunoactivating and cytotoxic to TNBC cells than hADSCs-IL2	no significant effect in murine *in vivo* model; lower proliferation stimulation vs. hADSCs-IL2
Chulpanova et al., 2023[18]	ATMSCs	CIMVs	flow cytometry (CD63, CD81, TRAIL), TEM, SEM, western blot, qPCR, and BCA	TRAIL	MCF-7	50 μg/mL CIMVs-TRAIL; apoptosis assays at 24h/72h; *in vivo* injections into MCF-7 xenografts in nude mice	↑ apoptosis (Annexin V, Casp8); ↑ BAX, CASP8 expression; ↓ viability	↑ tumor necrosis (*in vivo*); unchanged BCL2; BAX/BCL2 > 1	CIMVs-TRAIL activated extrinsic apoptosis pathway *in vitro* and *in vivo*; effect was moderate due to low surface TRAIL presence	low surface TRAIL (7% of vesicles); limited tumor growth inhibition despite apoptosis; single cancer model
Ding et al., 2023[19]	BMSCs	ultracentrifugation	TEM, NTA, western blot (CD9, CD63), zeta potential, and UV-vis	Ce6 + GW4869 (electroporation)	4T1	Ce6-GW4869/sEVs (Ce6: 15 mg/L, GW4869: 5 BμM), PDT (660 nm light), *in vitro* and *in vivo*: ROS assay, CCK-8, immunofluorescence, flow cytometry, and tumor models	↓ tumor volume; ↑ survival; ↑ ICD markers (CRT, ATP, HMGB1)	↓ Tregs/MDSCs; ↑ $CD8^{+}$ T cells; ↑ IFN-Oi/TNF-O±, PDT enhanced by sEV targeting	photosensitive sEVs suppressed TNBC via ICD & immune modulation; dual targeting (Ce6/PDT + GW4869) reversed immunosuppression and enhanced antitumor response	model limited to 4T1 and mice; large-scale production not addressed
Du et al., 2021[20]	UCMSCs	ultracentrifugation	TEM, flow cytometry (CD markers), and western blot (CD63, ALIX)	endogenous miR-21-5p	MCF-7 MDA-MB-231	hucMSC-EVs (20 μg/mL), miR-21-5p mimic/inhibitor modulation, ZNF367 overexpression; assays: transwell, qRT-PCR, western blot, and luciferase binding	↓ migration; ↓ invasion	↓ ZNF367; ↑ miR-21-5p; mimic enhanced effects; inhibitor reversed them	hucMSC-EVs inhibited metastasis via miR-21-5p/ZNF367 pathway; mimic strengthened suppression of ZNF367 overexpression, inhibiting invasiveness	no *in vivo* validation; mechanism limited to miR-21-5p
Ebrahimian et al., 2022[21]	ATMSCs	ultracentrifugation	TEM, SEM, western blot (CD9, CD63, CD81), BCA, DLS, and zeta potential	thymoquinone (Tq) via incubation, surfactant, and freeze-thaw	MCF-7	Tq@EXOs vs. free Tq, MTT, flow cytometry, and FITC-labeling uptake	↓ viability in MCF-7 (dose-dependent); no cytotoxicity in L929	↑ cellular uptake (FITC); 60% loading efficiency	Tq-loaded EVs inhibited MCF-7 with lower toxicity than free drug	no *in vivo* validation; limited to MCF-7
Egea et al., 2021[22]	BMSCs	ultracentrifugation	western blot, qRT-PCR (let-7f), microscopy, DLS, and zymography	endogenous let-7f (via cytokine or hypoxia stimulation)	4T1	let-7f mimic/inhibitor transfection; *in vitro* & *in vivo* 4T1 spheroids	↓ invasion and proliferation in 4T1 (via MMP-9, autophagy)	↑ $CD8^{+}$ cells *in vivo*; ↑ let-7f in EVs post hypoxia	let-7f in MSC-EVs impaired TNBC via autophagy and paracrine signaling	mouse 4T1 only; autophagy link needs deeper mechanistic validation
Eiro et al., 2024[23]	UCESCs	ultracentrifugation	TEM, NTA, western blot (CD9, CD63, CD81), flow cytometry, and BCA assay	paclitaxel (endogenous loading via preconditioning)	MDA-MB-231 TNBC	CM-hUCESC or CM-hUCESCchemo (with paclitaxel) B± paclitaxel; *in vitro*: WST-1, invasion, flow cytometry; *in vivo*: TNBC xenograft model	↓ proliferation; ↑ apoptosis; ↓ invasion (*in vitro*); ↓ tumor growth (*in vivo*)	↑ G2-M phase arrest; TIMP-1/2 identified as functional anti-invasive factors; EVs showed successful paclitaxel loading	CM-hUCESC synergized with paclitaxel; preconditioned secretome enhanced antitumor effects and reduced the needed paclitaxel dose	no direct comparison with other MSC sources; patient-specific effects not evaluated

(*Continued on next page*)

Table 1. *Continued*

Author(s), year (reference)	MSC source	EV isolation method	EV characterization	Loaded EV	Breast cancer cell line(s)	Intervention details	Primary outcomes	Secondary outcomes	Key findings	Limitations
Farhadi et al., 2023[24]	UCMSCs	EXOCIB Kit	TEM, DLS, western blot (CD63, CD81, Calnexin), BCA, qPCR, SEM	7SK long non-coding RNA (lncRNA)	MDA-MB-231	100 μg/mL Exo-7SK, treated 24–72 h; MTT, flow cytometry, qPCR, migration/invasion assays, and *in vivo* xenograft	↓ viability; ↑ apoptosis; ↓ proliferation; ↓ migration; ↓ invasion; ↓ tumor size	↓ BCL-2; ↑ BAX & p53; ↓ HMGA1 and 6 HMGA1 target genes (e.g., CHEK1 and, CENPF)	Exo-7SK suppressed TNBC growth *in vitro* and *in vivo* via HMGA1 pathway	stability of 7SK over time; apoptosis may bias qPCR quantification
Farouk et al., 2024[25]	BMSCs	ultracentrifugation	TEM, flow cytometry (CD63, CD81), and BCA assay	vincristine sulfate (VCR), via probe sonication	T47D	EXO-VCR vs. free VCR vs. unloaded EXO; SRB assay and $CD44^+$/CD24в€' CSC analysis (flow cytometry)	↓ viability (IC50: 0.01 μg/mL); ↓ CSCs to 2.8% from 10.5%	EXO alone had no toxicity; VCR-Exo maintained activity with better targeting	VCR-loaded EVs reduced CSC population without added toxicity; targeting enhanced	no *in vivo* confirmation; only one cell line tested
Felthaus et al., 2024[26]	ATMSCs	Pan Human EV Kit	western blot (CD63)	none	MCF-7	EVs (10–35 BμL/cmBI), compared with conditioned medium (CM) and control; viability (resazurin), cytotoxicity (LDH), and RT-PCR	↓ viability (dose-dependent); ↑ LDH release at 35 BμL/cmBI; ↑ TP53, Bax, Bad, Casp3, RB1	↓ BCL-2, VEGF-A, PDGF-A, PDGF-B, Ki67 vs. CM; effect not always significant vs. control	ADSC-EVs promoted apoptosis and downregulated angiogenesis/proliferation markers in MCF-7; opposite effect from CM	no ultracentrifugation; CD63 only for EV ID; *in vitro* only
Gomari et al., 2018[27]	MSCs	Cell Guidance Systems Kit	TEM, Zetasizer, western blot (His-tag), flow cytometry, and fluorescence imaging	doxorubicin (electroporation), LAMP2b-DARPin targeting HER2	BT-474<br>SKBR3<br>MDA-MB-231	Exo-Dox vs. free Dox; PKH67 labeling; cytotoxicity and uptake analysis; IC50 determination	targeted EVs increased uptake in $HER2^+$ cells (56.3% SKBR3 vs. 1.5% MDA-MB-231); IC50 comparable to free DOX	fluorescence microscopy showed nuclear accumulation of Dox; no added cytotoxicity over free Dox	engineered EVs selectively bound to $HER2^+$ cells and efficiently delivered DOX	no *in vivo* validation; limited comparison between targeted vs. free DOX cytotoxicity
Gomari et al., 2019[28]	BMSCs	Cell Guidance Systems kit; ultrafiltration	TEM, DLS, western blot (CD9, CD63, CD81, calnexin), Zeta-sizer	doxorubicin (electroporation); targeted with LAMP2b-DARPin	TUBO<br>SKBR3<br>4T1	Exo-Dox vs. free Dox versus PBS; *in vitro* MTT at 24–72 h; *in vivo* tumor inhibition in BALB/c mice (6 i.v. injections)	↓tumor volume *in vivo* with targeted Exo-Dox versus free/untargeted Dox; ↑ binding to HER2+ cells	↑ Dox accumulation in tumor; ↓fluorescence in heart; no weight loss at 1.5 mg/kg	targeted Exo-Dox reduced tumor volume and enhanced DOX targeting while reducing off-target effects	TUBO model only; low-dose Dox (1.5 mg/kg); possible underestimation of free DOX effects
Hass et al., 2024[29]	UCMSCs	ultracentrifugation; size exclusion chromatography	TEM, western blot (CD9, CD63, CD81, TSG101), NTA, and ELISA (SDF-1)	paclitaxel (Taxol) via MSC preconditioning	MDA-MB-231	taxol-EVs vs. free taxol (0.1–1000 nM); viability assay in 5 GFP-labeled tumor cell lines; miR profiling; SDF-1 blocking	↓viability (dose-dependent); ↑ apoptosis in MDA-MB-231; 11 antitumor miRs upregulated; ↓tumor viability *in vivo*	↑ SDF-1 (2.2–5.4×); effects reversed partially by anti-SDF-1 Ab; increased EV size/yield with taxol	taxol-EVs show enhanced tumor tropism and anticancer miR cargo; SDF-1-CXCR4/7 axis improved targeting	storage effects on SEC-EVs; *in vitro* model focus; miR function context-specific
Hosseini et al., 2024[30]	WJMSCs	EXOCIB Kit	TEM, SEM, DLS, flow cytometry (CD9, CD63, CD81), and western blot	S3I-201 (STAT3 inhibitor) via electroporation	4T1	WJ-Exo (S3I-201) vs. free S3I-201 (10–500 BμM); MTT, apoptosis, migration, STAT3 expression, qPCR, *in vivo* tumor model (3×10^6 cells); EV dose: 5–10 μg	↓viability; ↑ apoptosis; ↓migration; ↓p-STAT3; ↓tumor volume and weight; ↑ survival	↓Bcl-2; ↑ Bax; ↑ Caspase-3; ↑ IFN-Oi; ↑ TNF-O±; ↓IL-4; ↓IL-1OI; ↑ splenocyte proliferation	WJ-Exo (S3I-201) enhanced antitumor efficacy *in vitro* and *in vivo* by suppressing STAT3 and modulating Th1/Th2 balance	no comparison with other EV sources; mouse-specific model

(*Continued on next page*)

Table 1. *Continued*

Author(s), year (reference)	MSC source	EV isolation method	EV characterization	Loaded EV	Breast cancer cell line(s)	Intervention details	Primary outcomes	Secondary outcomes	Key findings	Limitations
Hu et al., 2022[31]	BMSCs	ultracentrifugation	TEM, NTA, and western blot (CD63, TSG101, Calnexin)	ALKBH5 shRNA	MDA-MB-231	BMSC-Exos with/without ALKBH5 shRNA; *in vitro* and *in vivo* assays for tumor volume, apoptosis, stemness markers, and metastasis	↓ tumor volume/weight; ↓ stemness (NANOG, SOX2, OCT4); ↓ metastasis	↓ UBE2C; ↑ p53; ↓ Ki67, lung metastases	ALKBH5 shRNA-loaded Exos suppressed TNBC stemness and metastasis via UBE2C/p53 axis	mechanism confirmed only in MDA-MB-231; long-term effects unknown
Jafarpour et al., 2024[32]	ATMSCs	Exo-Spin Kit	TEM, DLS, zeta potential, Bradford assay, and ExoGlow labeling	paclitaxel and cisplatin (via sonication)	MDA-MB-231<br>BT-474	EXO-PTX and EXO-CIS vs. free drugs; viability (MTT), apoptosis (Annexin V/PI), uptake (flow cytometry)	↑ apoptosis; ↓ viability; Exo-PTX superior to PTX; Exo-CIS ∼ CIS	better spheroid penetration, enhanced cytotoxicity with lower drug dose	drug-loaded EVs improved therapeutic efficacy in 3D spheroids	no *in vivo* model; limited mechanistic exploration
Jia et al., 2020[33]	ATMSCs	sequential centrifugation	TEM, western blot (CD9, CD63, CD81, HSP70), and flow cytometry	miR-1236 (natural content)	MCF-7<br>MDA-MB-231	20 Ojg/mL adMSC-Exos *in vitro*; measured resistance to cisplatin (DDP)	↓ IC50 of DDP; ↑ apoptosis; ↑ caspase-3; ↓ proliferation	↓ SLC9A1 expression; ↓ Wnt/OI-catenin pathway activity	adMSC-Exos carrying miR-1236 sensitized breast cancer cells to cisplatin by downregulating SLC9A1 and suppressing Wnt/OI-catenin signaling	*in vitro* only; *in vivo* validation lacking
Kaan, 2023[34]	ATMSCs	EXOTC50A-1 Kit	SEM, NTA, and zeta potential	melatonin	MDA-MB-231<br>MCF10A	Mel (2.5 mM) + Exo (25–100 μg/mL) for 24, 48, and 72 h	↓ cell viability (XTT assay); ↑ apoptosis (Annexin V/PI)	IC50 values: 30.38 μg/mL (48 h); max apoptosis at 24h (6.3%)	melatonin + EV co-administration synergistically induced cytotoxicity and apoptosis in TNBC cells	*in vitro* only; no animal model validation
Kalimuthu et al., 2018[35]	BMSCs	ultracentrifugation	TEM, NTA, and western blot (CD63, ALIX, calnexin-, and GM130-)	paclitaxel (PTX)	MDA-MB-231<br>MCF-7<br>MCF-10A	PTX-loaded EMs (10–100 μg), *in vitro* (viability, Fluc activity), *in vivo* (xenograft tumor model)	↓ tumor growth and viability *in vitro* and *in vivo*	EMs had high PTX loading efficiency, better than native EVs	PTX-loaded EMs effectively delivered drug and inhibited breast cancer growth	nontargeted delivery affected normal cells
Khazaei-Poul et al., 2021[36]	UCMSCs	EXOCIB Kit	TEM, SEM, DLS, and western blot (CD63, CD81)	miR-3182 (via electroporation)	MDA-MB-231	100 Ojg/mL HUCMSC-Exos loaded with miR-3182; *in vitro* assays (MTT, apoptosis, migration, scratch, and colony formation); *in vivo* not conducted	↓ viability, migration, colony formation; ↑ apoptosis; ↓ mTOR, S6KB1 expression	cell-cycle arrest (↓ S and G2/M); dual-luciferase confirmed miR-3182 targets mTOR/S6KB1	miR-3182-loaded Exos suppressed TNBC growth and metastasis via mTOR/S6KB1 inhibition	no *in vivo* validation; only one cell line used
Khazaei-Poul et al., 2023[37]	UCMSCs	ultracentrifugation	TEM, NTA, DLS, and WB (CD63, CD81, TSG101)	miR-3143 and miR-3182 via electroporation	MDA-MB-231	*in vitro*: qRT-PCR of critical genes after exosomal miR-3143/3182 delivery; *in vivo*: none	↓ expression of cell cycle-related oncogenes (FOSL1, MELK, GINS2, CCNA2, DSN1, TGFOI2, E2F7, and GATA6)	network analysis (GRN/PPI); confirmed miR-gene targeting by RT-qPCR	exosomal delivery of miR-3143/3182 downregulated key cell cycle and survival genes in TNBC	no *in vivo* validation; only gene expression measured

(*Continued on next page*)

Table 1. *Continued*

Author(s), year (reference)	MSC source	EV isolation method	EV characterization	Loaded EV	Breast cancer cell line(s)	Intervention details	Primary outcomes	Secondary outcomes	Key findings	Limitations
Lee et al., 2013[38]	BMSCs	ExoQuick	TEM, western blot (CD63, calnexin-), and bioanalyzer	none	4T1	MSC-EVs (25–100 μg/mL); *in vitro* assays (qRT-PCR, ELISA, migration, tube formation); *in vivo* (xenograft)	↓ VEGF expression; ↓ angiogenesis; ↓ tumor volume	miR-16 was transferred to tumor cells; inhibited VEGF/VEGFR1 axis	MSC-EVs delivered miR-16, suppressing angiogenesis and tumor growth	mouse model only, no human data
Liu et al., 2020[39]	BMSCs	Invitrogen EV Isolation reagent	TEM, western blot (CD63, CD81, and ALIX), and fluorescence	CXCR4 and TRAIL (via lentiviral vector)	MDA-MB-231	4 mg/kg ExoCXCR4 + TRAIL B ± 5 mg/kg carboplatin; brain metastasis mouse model	↓ bioluminescence signal, tumor growth in brain; ↑ apoptosis	enhanced delivery via CXCR4; TRAIL-induced tumor cell death	ExoCXCR4 + TRAIL synergized with carboplatin against brain metastasis	lacks *in vitro* mechanistic assays; limited dosage range
Liu et al., 2022[40]	BMSCs	ExoQuick	TEM, NTA, and western blot (CD63, TSG101, ALIX, and calnexin-)	miR-342-3p	MCF-7<br>T47D, MDA-MB-231<br>SKBR3	EVs B ± miR-342-3p mimic/inhibitor; migration, invasion, apoptosis, xenograft, and metastasis assays	↓ tumor volume; ↓ migration/invasion; ↑ apoptosis	↓ INHBA; ↓ IL13RO±2; ↑ E-cadherin; ↓ vimentin	EV-miR-342-3p suppressed breast cancer via INHBA/IL13RO±2 axis	rat MSCs; human validation needed
Melzer et al., 2019[41]	UCMSCs	ultracentrifugation	TEM, NTA, WB (CD9, CD63, CD81), and zeta potential	taxol (via passive exposure, 10 BμM for 24 h)	MDA-hyb1	*in vitro*: 1:150 EV dilution; *in vivo*: systemic i.v. injection (4×) into NODscid mice	↓ viability (∼80–90%), ↓ tumor weight by 64.2%; ↓ metastases (lung, liver, spleen, and kidney)	↑ apoptosis; ↓ Ki-67, ↓ VEGF/angiogenesis markers, 34-fold targeting efficiency	taxol-loaded MSC-EVs achieved potent *in vivo* anti-tumor and anti-metastatic effects with 1000× less taxol than free drug	no clinical translation yet; EVs not purified beyond ultracentrifugation
Melzer et al., 2020[42]	MSC544	ultracentrifugation	TEM, western blot (CD9, CD63, CD81), NTA, and zeta potential	taxol or epirubicin	MDA-hyb1	*in vitro* (100 Ojg/mL EVs for 72h); *in vivo* (4× i.v. injections in TNBC xenograft mice)	↓ viability (86.9% for taxol-EVs), ↓ tumor volume (74%) with i.v. taxol-EVs	epirubicin-EVs also cytotoxic; taxol-EVs had longer retention in tumors	taxol-/epirubicin-loaded EVs from MSC544 suppressed TNBC growth and metastasis *in vitro* and *in vivo*	lack of detailed toxicity profiling and long-term safety assessment
Meng et al., 2023[43]	BMSCs	ultracentrifugation	TEM, NTA, zeta potential, western blot (CD63, CD81, and HSP70), and mass spectrometry	paclitaxel (PTX), anti-EGFR, anti-HER2, anti-IL12P40, and biotin-GALA (via sonication + biotin-streptavidin system)	MDA-MB-468 (EGFR+), MDA-MB-453 (HER2+)	*in vitro*: PTX@SA-EVs and PTX@anti-EGFR/anti-HER2/GALA-SA-EVs; *in vivo*: xenograft mice injected with SA-EVs or modified EVs (4–4.5 mg/kg PTX)	↓ viability; ↑ apoptosis; ↓ tumor volume (max in GALA- or EGFR-modified PTX-EVs)	↓ Ki-67; ↑ TUNEL; ↓ IL-6/IL-1OI/TNF-O± in RA model; and improved bone structure in CIA mice	streptavidin-overexpressing BMSC-EVs serve as a universal delivery platform with surface modification for tumor targeting; GALA/EGFR/HER2/IL12P40 improves targeting and drug delivery	no clinical data; long-term safety not assessed
Mirabdollahi et al., 2020[44]	WJMSCs	natural secretome	flow cytometry, MTT, and histopathology	none	MCF-7<br>4T1	*in vitro* (2–20 mg/mL); *in vivo* intratumoral (20 mg/inj)	↓ tumor size and weight, ↑ latency; ↑ survival rate	improved hematological parameters	hWJMSC-secretome inhibited breast cancer growth *in vitro* and *in vivo*	secretome not characterized molecularly; non-EV content confounding
Mohd Ali et al., 2020[45]	ATMSCs	ultracentrifugation; membrane spin columns	TEM, AChE activity, CD63/CD81/CD9/TSG101, and RNA profiling	natural miRNA cargo (from co-culture)	MCF7<br>MDA-MB-231	co-culture with MSCs; EV collection; *in vitro* migration, invasion, cell cycle, apoptosis, and RNA seq	↓ proliferation, migration, invasion, and EMT; ↑ dormancy markers	upregulation of miR-200a-5p, miR-629-5p; downregulation of miR-10b-5p, miR-486-5p	MSC-EVs induce dormancy and inhibit EMT/metastasis via exosomal miRNAs	no *in vivo* validation

(*Continued on next page*)

Table 1. *Continued*

Author(s), year (reference)	MSC source	EV isolation method	EV characterization	Loaded EV	Breast cancer cell line(s)	Intervention details	Primary outcomes	Secondary outcomes	Key findings	Limitations
Naseri et al., 2018[46]	BMSCs	ExoQuick	TEM, western blot (CD63/CD81), DLS, and Zetasizer	LNA-anti-miR-142-3p	4T1<br>TUBO	5 μg EVs *in vitro* (48 h); 30 μg EVs i.v. q48h in mice	↓ miR-142-3p/miR-150; ↑ APC & P2X7R expression; ↑ apoptosis; ↓ tumor volume	increased survival, tumor tropism validated by PKH67 EV imaging	MSC-EVs are effective nanocarriers for LNA-miRNA inhibitors and suppress tumor growth	mouse-derived MSCs; human MSC validation needed
Naseri et al., 2020[47]	BMSCs	ExoQuick	TEM, western blot (CD63/CD81), DLS, and Zetasizer	LNA-anti-miR-142-3p	MCF-7<br>BCSC<br>MCF-10	5 Ojg EVs *in vitro* for 48 h; 30 Ojg EVs i.v. every 48 h in mice	↓ miR-142-3p/miR-150; ↓ clonogenicity; ↓ tumorigenicity	↑ APC & P2X7R gene expression; ↑ apoptosis; ↑ survival	MSC-derived EVs loaded with LNA-anti-miR-142-3p suppressed tumor growth and stemness in breast cancer stem cells	mouse MSCs; human validation not performed
O'Brien et al., 2018[48]	BMSCs	ultracentrifugation	NTA, TEM, CD63 WB, and miRNA profiling	miR-379 (engineered)	T47D<br>HCC1954	systemic EV injection (4×, 2.6 × 10^7 EVs in 50 Bμl PBS)	↓ tumor growth; ↓ COX-2 expression; ↑ necrosis	↓ TIMP-1, serpin E1, uPA *in vitro*; HCC-1954: high necrosis	systemic delivery of EV-encapsulated miR-379 suppressed tumor growth and COX-2 pathway *in vivo*	cell therapy with MSCs alone ineffective; miR-379 effective only in EV form
Pakravan et al., 2017[49]	BMSCs	ultracentrifugation	SEM, DLS, and WB (CD9 and calnexin)	natural miR-100	MDA-MB-231	EV dose 20–80 Ojg/mL; anti-miR-100 rescue; HUVEC co-culture	↓ VEGF (mRNA/protein); ↓ mTOR/HIF-1O±	↓ HUVEC proliferation, migration, and tube formation	MSC-derived exosomal miR-100 suppressed angiogenesis via VEGF inhibition in breast cancer cells	no *in vivo* study; only *in vitro* data
Patel et al., 2021[50]	UCMSCs	ultracentrifugation; PEG precipitation	NTA, TEM, and western blot (CD63, CD9, and syntenin)	cannabidiol (CBD) via sonication	MDA-MB-231	CBD EVs (1 BμM) B± DOX (500 nM); *in vitro* cytotoxicity, cell cycle, western blot, migration; *in vivo*: xenograft model in nude mice	↓ viability; ↑ G1 arrest; ↑ apoptosis; ↓ tumor burden (CBD EVs + Dox most effective)	↓ NF-κB, IL-17, STAT3, Bcl-2; ↑ BAX, caspase-3, caspase-9; ↓ integrin O±5, Twist, GPC1, GPC6, Smad2	CBD EVs synergized with Dox in TNBC by suppressing metastasis, inflammation, and enhancing apoptosis	no long-term toxicity data; complex bioreactor system scalability untested
Ramirez et al., 2024[51]	ATMSCs	natural secretome	not fully characterized as EVs; focus on secretome via qPCR, multiplex	natural secretome	MCF7	hAMSC-CM with/without natural plant extracts (Anamu-SC or P2Et); MTT, Alamar Blue, colony, and wound assays	↓ cell viability; ↓ clonogenicity; ↓ migration	modulation of secretome gene expression (↓ IL-6, RANTES)	hAMSC-CM enhanced cytotoxicity of natural plant extracts and inhibited tumor cell migration	used secretome not purified EVs; *in vitro* only
Rezaie et al., 2018[52]	ATMSCs	ultracentrifugation	SEM, gene expression, and apoptosis markers	none	MCF-7	microvesicles delivered via PCL nanofibers; 20 Ojg/mL; RT-PCR, MTT, and SEM	↓ viability (MTT); ↑ apoptosis (↑ p53 and Bax; ↓ Bcl2)	↑ E2F5 and SMAD5; morphology changes observed	MSC-derived microvesicles induced apoptosis in MCF-7, especially with prolonged release from nanofibers	no *in vivo* study; no comparison to drug treatments
Sandiford et al., 2021[53]	BMSCs	ultracentrifugation EV Isolation Kit	western blot, TEM, NTA, and flow cytometry	none	MDA-MB-231<br>MDA-MB-468<br>T47D	EVs (10^8 particles) added every other day for 7 days	↑ stemness genes (Oct4a, Nanog, and KLF4); ↑ G0-quiescence; ↑ tumor sphere formation; ↑ chemoresistance	↓ ROS; ↓ mitochondrial activity; ↑ autophagy; ↑ NF-κB activity	MSC-EVs induced dedifferentiation of BCCs into dormancy-associated CSCs	complex model, limited clinical translation; mixed EV populations

(*Continued on next page*)

Table 1. *Continued*

Author(s), year (reference)	MSC source	EV isolation method	EV characterization	Loaded EV	Breast cancer cell line(s)	Intervention details	Primary outcomes	Secondary outcomes	Key findings	Limitations
Sheykhhasan et al., 2021[54]	ATMSCs	EXOCIB Kit	TEM, SEM, DLS, and FACS (CD63, CD81)	miR-145 via lentiviral transfection	T-47D	EVs from MSCs transfected with miR-145; 100 µg/mL; real-time PCR, migration and gene expression studies	↓ ERBB2; ↓ ROCK1; ↓ MMP9; ↑ p53	highest miR-145 expression in the miR-MSC-Exo group; more effective than direct transfection	exosomal delivery of miR-145 effectively downregulated metastasis and apoptosis-related genes	no protein-level confirmation; *in vitro* only
Shojaei et al., 2021[55]	ATMSCs	EXOCIB Kit	SEM, TEM, DLS, and western blot (CD63, CD81)	miR-381 mimic via electroporation	MDA-MB-231	100 Ojg/mL of EVs loaded with miR-381 for 72 h	↓ viability, ↑ apoptosis (Annexin V/PI), and ↓ migration/invasion (scratch and transwell)	↓ Twist, Snail, LRP6, CTNNB1; ↑ E-cadherin, and ↓ N-cadherin (qPCR and western blot)	miR-381 delivered by MSC-EVs inhibited EMT and aggressiveness in TNBC cells	no *in vivo* validation; short-term treatment
Shojaei et al., 2023[56]	ATMSCs	EXOCIB Kit	DLS, TEM, and SEM	miR-218 mimic via electroporation	MDA-MB-231	100 µg/ml EVs loaded with miR-218; assays: qRT-PCR, MTT, Annexin V/PI, migration, invasion, and tube formation	↓ viability; ↑ apoptosis; ↓ invasion; ↓ migration	↓ Runx2; ↓ Rictor; ↓ CDH2; ↑ CDH1; ↓ VEGF	miR-218-enriched EVs reversed EMT and angiogenesis in TNBC cells	*in vitro* study only
Sun et al., 2022[57]	BMSCs	ultracentrifugation	TEM and western blot (CD9, CD63, and HSP70)	miR-139-5p (natural content)	MDA-MB-231	BMSC-EVs (200 BµL) co-cultured with MDA-MB-231 for 48 h	↓ cell viability; ↓ expression of FBN2, MEX3A; TPD52	↑ miR-139-5p, comparative with normal cells (MCF-10A)	exosomal miR-139-5p from BMSCs inhibited MDA-MB-231 growth by targeting cancer genes	*in vitro* only; no *in vivo* validation
Ulpiano et al., 2025[58]	WJMSCs	tangential flow filtration; anion exchange chromatography	NTA, TEM, western blot (CD9, CD63, and syntenin), zeta potential, and FACS	none	MDA-MB-231 MCF-7	EVs (2×10^{10}/mL) incubated with cells for 6 h; internalization via FACS	high uptake by MDA-MB-231 and MCF-7 (no viability data)	EVs retained identity, quality, size, charge, and purity after production	developed GMP-compliant large-scale EV production platform; confirmed breast cancer cell uptake	no functional assay or therapeutic outcome tested
Vakhshiteh et al., 2021[59]	BMSCs	ultracentrifugation	TEM, DLS, zeta potential, WB (CD63, TSG101), AO/EB, and BCA	miR-34a via lentiviral transfection	MDA-MB-231	EVs at 10 and 50 µg/mL; 24 h and 48 h assays (CCK-8, apoptosis, and qRT-PCR)	↓ viability; ↑ apoptosis (AO/EB staining, and annexin-V/PI)	↓ Bcl-2, Notch1, Nanog, Survivin, CD44, Ki-67; ↑ Caspase-3, Bax	exosomal delivery of miR-34a induced apoptosis and inhibited proliferation via Notch1 and stemness gene suppression in TNBC cells	*in vitro* only; no *in vivo* or functional delivery kinetics tested
Wang et al., 2021[60]	UCMSCs	ultracentrifugation	TEM, NTA, and western blot (CD9, CD63, and HSP70)	miR-224-5p (via mimic or inhibitor transfection)	MCF-7 MDA-MB-231	EVs from hUCMSCs transfected with miR-224-5p mimic or inhibitor co-cultured with cells; *in vivo*: subcutaneous xenografts with serial injections	↑ proliferation; ↓ apoptosis; ↑ autophagy (↑ LC3-II, Beclin-1; ↓ p62); *in vivo*: ↑ tumor volume, ↓ Ki-67 with inhibitor	miR-224-5p targets HOXA5; downregulates its expression; HOXA5 negatively regulates autophagy	miR-224-5p carried by hUCMSC-EVs promoted breast cancer cell proliferation and autophagy via HOXA5 suppression	only two cell lines tested; no systemic delivery *in vivo*
Xu et al., 2024[61]	PMSCs	ultracentrifugation; ExoQuick	TEM, NTA, western blot (CD9, CD63, CD81, TSG101), and UV absorbance	doxorubicin + Fe_3O_4 nanoparticles (magnetic targeting)	MDA-MB-231 4T1	*in vitro* uptake, migration, invasion, apoptosis; *in vivo* tumor xenografts with or without magnetic field	↓ viability; ↑ apoptosis; ↓ migration and invasion; ↓ tumor volume	↓ cardiomyocyte damage; ↑ Dox delivery efficiency; ↑ targeting specificity	magnetized EVs loaded with Dox enhanced tumor-specific delivery, suppressed tumor progression, and reduced cardiotoxicity	no long-term safety data; limited models tested

(*Continued on next page*)

Table 1. ***Continued***

Author(s), year (reference)	MSC source	EV isolation method	EV characterization	Loaded EV	Breast cancer cell line(s)	Intervention details	Primary outcomes	Secondary outcomes	Key findings	Limitations
Yang et al., 2022[62]	BMSCs	ultracentrifugation	TEM, NTA, and western blot (CD63, CD81, CD9, TSG101, and GRP94)	miR-551b-3p (via agomir transfection)	MDA-MB-231<br>MCF-7<br>SK-BR-3	*in vitro* proliferation, migration, invasion, and apoptosis assays; xenograft mouse model	↓ proliferation; ↓ migration; ↑ apoptosis; ↓ tumor growth	↓ TRIM31; ↓ Akt phosphorylation; ↑ Bax & cleaved caspase-3; ↓ Bcl-2; ↓ GSH/SOD	exosomal miR-551b-3p inhibited breast cancer progression by targeting TRIM31/Akt pathway	mechanism validated in limited cell lines; clinical translation not explored
Zhang et al., 2022[63]	BMSCs	ultracentrifugation	TEM, NTA, and western blot (CD9, CD81, GRP94)	miR-16-5p (via mimic or inhibitor transfection)	MDA-MB-231<br>SK-BR-3	EVs from BMSCs transfected with miR-16-5p mimic or inhibitor co-cultured with cells; *in vivo*: xenograft in nude mice (5 mg EVs per mouse, tail vein)	↑ apoptosis; ↓ proliferation; ↓ migration; ↓ EMT (↓ N-cadherin, ↑ E-cadherin); ↓ tumor volume	miR-16-5p targets EPHA1 and suppresses NF-κB signaling; knockdown of EPHA1 mimics miR-16-5p effects	BMSC-EVs with miR-16-5p inhibited EMT and tumor progression via EPHA1/NF-κB axis	no direct human data; limited EV dosage/ time point evaluation
Zhang et al., 2024[64]	ATMSCs	ultracentrifugation	TEM, NTA, WB (CD9, TSG101), qPCR for miRNA, and flow cytometry	miR-588 mimic via electroporation; surface cRGD modification (DSPE-PEG2000-cRGD)	MDA-MB-231	*in vitro* (qPCR, ELISA, CCK-8, and cytokine profiling); *in vivo*: cRGD-Exos/miR-588 (5 nmol) i.v. q3d x5	↓ viability; ↑ apoptosis; ↓ CCL5/ TGF-OI; ↓ tumor volume	↓ M2 macrophages; ↑ M1 macrophages; ↑ IFN-Oi/TNF-O±/IL-6; ↓ IL-10; ↑ caspase-3/ 7; LDH release	engineered cRGD-EVs carrying miR-588 targeted TNBC and suppressed tumor via immune remodeling	single tumor model; long-term toxicity not evaluated
Zhou et al., 2021[65]	ATMSCs	ultracentrifugation	TEM, WB (CD9, CD63, CD81), NTA, and RNA bioanalyzer	miR-424-5p mimic (via transfection of AT-MSCs)	MDA-MB-231<br>HCC1954	EV-424 applied to cells B± PBMCs; *in vivo*: intratumoral injection of 30 μg EVs q72h × 4	↓ PD-L1 expression; ↑ apoptosis (caspase-3/ 7, LDH); ↓ tumor volume (*in vivo*)	↑ IFN-Oi, TNF-O±, IL-6; ↓ IL-10; altered M1/M2 macrophage ratio	AT-MSC-EVs carrying miR-424-5p suppressed PD-L1, remodeled immune microenvironment, and inhibited TNBC	intratumoral not systemic delivery; immunocompromised mouse model
Zhou et al., 2024[66]	PMSCs	ultracentrifugation	TEM, NTA, and western blot (TSG101, ALIX, and CD63)	none	4T1<br>MCF-7	*in vitro*: 50–200 μg/mL hPMSC-EVs; *in vivo*: 100 μg EVs peritumoral injection (days 0, 2, and 4)	↓ proliferation; ↓ migration; ↓ colony formation; ↓ angiogenesis; ↓ tumor growth	↓ Ki-67; ↓ VEGFA/ VEGFR2, Ang-1/2, bFGF, HIF-1O±, and PDGF (HUVECs and tumor)	hPMSC-EVs suppressed breast cancer progression by indirectly inhibiting angiogenesis via tumor-HUVEC crosstalk	no long-term toxicity data; no mechanistic cargo profiling

ATMSCs, adipose-derived mesenchymal stem cells; BMSCs, bone marrow-derived mesenchymal stem cells; CIMVs, cytochalasin B-induced membrane vesicle formation; DPSCs, dental pulp mesenchymal stem cells; MenSCs, menstrual mesenchymal stem cells; MSCs, mesenchymal stem cells; PMSCs, placental mesenchymal stem cells; UCESCs, uterine cervical mesenchymal stem cells; UCMSCs, umbilical cord mesenchymal stem cells; WJMSCs, Wharton's jelly-derived mesenchymal stem cells.

Table 2. Country and source of fundings of the *in vitro* studies on mesenchymal stem cell-derived extracellular vesicle therapy in breast cancer

Author(s), year (reference)	Country	Funding source
Ababneh et al., 2025[9]	Jordan	not stated
Aldiqs et al., 2025[10]	Jordan	not stated
Altanerova et al., 2019[11]	Slovakia and Czech	Slovak League against Cancer; Ministry of Education (Czech Republic)
Attar et al., 2025[12]	Iran	not stated
Bliss et al., 2016[13]	USA	Department of Defense (W81XWH-11-1-0276)
Casson et al., 2018[14]	UK	BBSRC (BB/L008661/1)
Chang et al., 2022[15]	Japan	Japanese Ministry of Education, Culture, Sports, Science & Technology (MEXT)
Chulpanova et al., 2021a[16]	Russia	RFBR (18-44-160024), Kazan Federal University Strategic Program
Chulpanova et al., 2021b[17]	Russia	Russian Science Foundation (18-74-10044), Kazan Federal University
Chulpanova et al., 2023[18]	Russia	Russian Science Foundation (18-74-10044); Kazan Federal University
Ding et al., 2023[19]	China	Jiangsu Province (Six Talent Peaks, 333 Project, M2022105)
Du et al., 2021[20]	China	not stated
Ebrahimian et al., 2022[21]	Iran	INSF and Mashhad University of Medical Sciences (Grant 961956)
Egea et al., 2021[22]	Germany and USA	DFG, German Federal Ministry of Defense, DZHK, UCSF
Eiro et al., 2024[23]	Spain	Instituto de Salud Carlos III, ERDF/ESF, Principality of Asturias, Spanish Ministry of Science and Innovation
Farhadi et al., 2023[24]	Iran	Eterna Biosciences Inc., Canada and Behbalin Inc., Iran
Farouk et al., 2024[25]	Egypt	Science, Technology & Innovation Funding Authority (STDF), Egypt
Felthaus et al., 2024[26]	Germany	not stated
Gomari et al., 2018[27]	Iran	not stated
Gomari et al., 2019[28]	Iran	Tarbiat Modares University (PhD thesis)
Hass et al., 2024[29]	Germany	NiedersΓ¤chsische Krebsgesellschaft e.V. ('Hand in Hand fΓjr Norddeutschland 2019′)
Hosseini et al., 2024[30]	Iran	Iran National Science Foundation (Grant 4001939)
Hu et al., 2022[31]	China	Jiangsu Cancer Hospital (202013); Jiangsu TCM Science Fund (2020)
Jafarpour et al., 2024[32]	Iran	Vice-Chancellor for Research, Isfahan Univ. of Medical Sciences (Grant 397811)
Jia et al., 2020[33]	China	not stated
Kaan, 2023[34]	Turkey	not stated
Kalimuthu et al., 2018[35]	South Korea	NRF Korea (2014R1A5A2009242), Korea Health Ministry (HI16C1501)
Khazaei-Poul et al., 2021[36]	Iran	Shahid Beheshti University of Medical Sciences
Khazaei-Poul et al., 2023[37]	Iran	Shahid Beheshti University of Medical Sciences (Grant #20575)
Lee et al., 2013[38]	South Korea	Global Core Research Center (GCRC), NRF Korea
Liu et al., 2020[39]	China	NSFC (81472818); Zhejiang Provincial Research Grants
Liu et al., 2022[40]	China	not stated
Melzer et al., 2019[41]	Germany	Erich and Gertrud Roggenbuck-Stiftung; DFG and Open Access Publication Fund of MHH
Melzer et al., 2020[42]	Germany	not stated
Meng et al., 2023[43]	China	National Natural Science Foundation of China (81872196, 81972541, 81772900, and 81672690)
Mirabdollahi et al., 2020[44]	Iran	Isfahan University of Medical Sciences
Mohd Ali et al., 2020[45]	Malaysia	not stated
Naseri et al., 2018[46]	Iran	Mashhad University of Medical Sciences
Naseri et al., 2020[47]	Iran	Mashhad University of Medical Sciences
O'Brien et al., 2018[48]	Ireland	not stated
Pakravan et al., 2017[49]	Iran	Tarbiat Modares University, Iran
Patel et al., 2021[50]	USA	Consortium for Medical Marijuana Clinical Outcomes Research, NIH (U54 MD007582), NSF-CREST (1735968)
Ramirez et al., 2024[51]	Colombia	not stated
Rezaie et al., 2018[52]	Iran	not stated

(*Continued on next page*)

Table 2. *Continued*

Author(s), year (reference)	Country	Funding source
Sandiford et al., 2021[53]	USA	not stated
Sheykhhasan et al., 2021[54]	Iran	not stated
Shojaei et al., 2021[55]	Iran	not stated
Shojaei et al., 2023[56]	Iran	Shahid Beheshti University of Medical Sciences
Sun et al., 2022[57]	China	not stated
Ulpiano et al., 2025[58]	Portugal	not stated
Vakhshiteh et al., 2021[59]	Iran	not stated
Wang et al., 2021[60]	China	not stated
Xu et al., 2024[61]	China	not stated
Yang et al., 2022[62]	China	not stated
Zhang et al., 2022[63]	China	NSFC, China Postdoc Foundation, Shanxi Provincial Science Fund
Zhang et al., 2024[64]	China	not explicitly stated
Zhou et al., 2021[65]	China and Japan	not stated
Zhou et al., 2024[66]	China	Tianjin Natural Science Foundation, Nankai University Eye Institute, Tianjin Key Medical Discipline

EV characterization, with no reference to MISEV (Minimal Information for Studies of Extracellular Vesicles) guidelines. Bliss et al.'s[13] and Mirabdollahi et al.'s[44] studies were rated high for detection bias, primarily due to the lack of clarity in outcome measurement procedures and absence of technical replicates. Studies by Mohd Ali et al.[45] and Pakravan et al.[49] demonstrated high attrition bias, as they failed to report all relevant outcome data or omitted results without explanation. Additionally, studies by Casson et al.[14] and Du et al.[20] were classified as having unclear performance bias, given their limited information regarding the consistency of experimental procedures.

Blinding of investigators and outcome assessors, a common limitation of *in vitro* experiments, was not reported in any of the included studies. Furthermore, while EV characterization was commonly mentioned, only a few studies adhered to MISEV guidelines, raising concerns about the reproducibility and standardization across the field.

While the overall quality of the included *in vitro* studies was acceptable, with most studies exhibiting a low risk of bias, notable deficiencies were observed in a subset of studies. These included inadequate EV characterization, incomplete outcome reporting, and insufficient methodological transparency. Addressing these issues in future research will be critical to improving the rigor and reproducibility of *in vitro* studies on MSC-EVs in breast cancer.

In future research, authors should adopt the MISEV 2023 guidelines to ensure comprehensive reporting of EV isolation, characterization, and quantification parameters. Consistent application of these standards will markedly improve reproducibility, data transparency, and cross-study comparability in MSC-EV research. Of the 58 studies, 19 explicitly referenced MISEV 2018/2023 guidelines, 31 partially fulfilled core characterization criteria (TEM + CD63/CD81/CD9 + functional assay), and 8 did not report sufficient details.

Meta-analysis results

Viability

A total of 38 studies were included in this meta-analysis, comprising 136 subjects in the experimental cohort and 136 subjects in the control cohort. The analysis was conducted using a random-effects model with the inverse variance method to compare the standardized mean differences (SMDs) in cancer cell viability between the MSC-EV-treated groups and the control groups. The pooled analysis demonstrated a statistically significant reduction in cancer cell viability following MSC-EV treatment, with a summarized SMD of −4.79 and a 95% confidence interval (CI) ranging from −6.03 to −3.54. The test for overall effect confirmed statistical significance ($p < 0.05$), indicating a meaningful therapeutic impact of MSC-EVs *in vitro*.

Heterogeneity among the studies was statistically significant ($p < 0.01$), with an I^2 value of 62.5%, suggesting that over half of the variability in effect estimates was due to true heterogeneity, rather than random error. To investigate the sources of heterogeneity, subgroup analyses were performed based on both the type of breast cancer cell line and the source of MSCs.

When stratified by the breast cancer cell line, the subgroup analysis revealed varying magnitudes of the effect (Figure 3). In studies using MCF7 cells ($n = 30$), the pooled SMD was −3.51 (95% CI: −6.30 to −0.72) with substantial heterogeneity ($I^2 = 71.4\%$). Studies involving MDA-MB-231 cells ($n = 54$) showed a significant pooled SMD of −3.83 (95% CI: −5.05 to −2.61), with moderate heterogeneity ($I^2 = 49.7\%$). Notably, the 4T1 subgroup ($n = 18$) showed the most pronounced effect, with an SMD of −7.39 (95% CI: −12.71 to −2.06) and an I^2 value of 59.7%. Other subgroups including T47D,

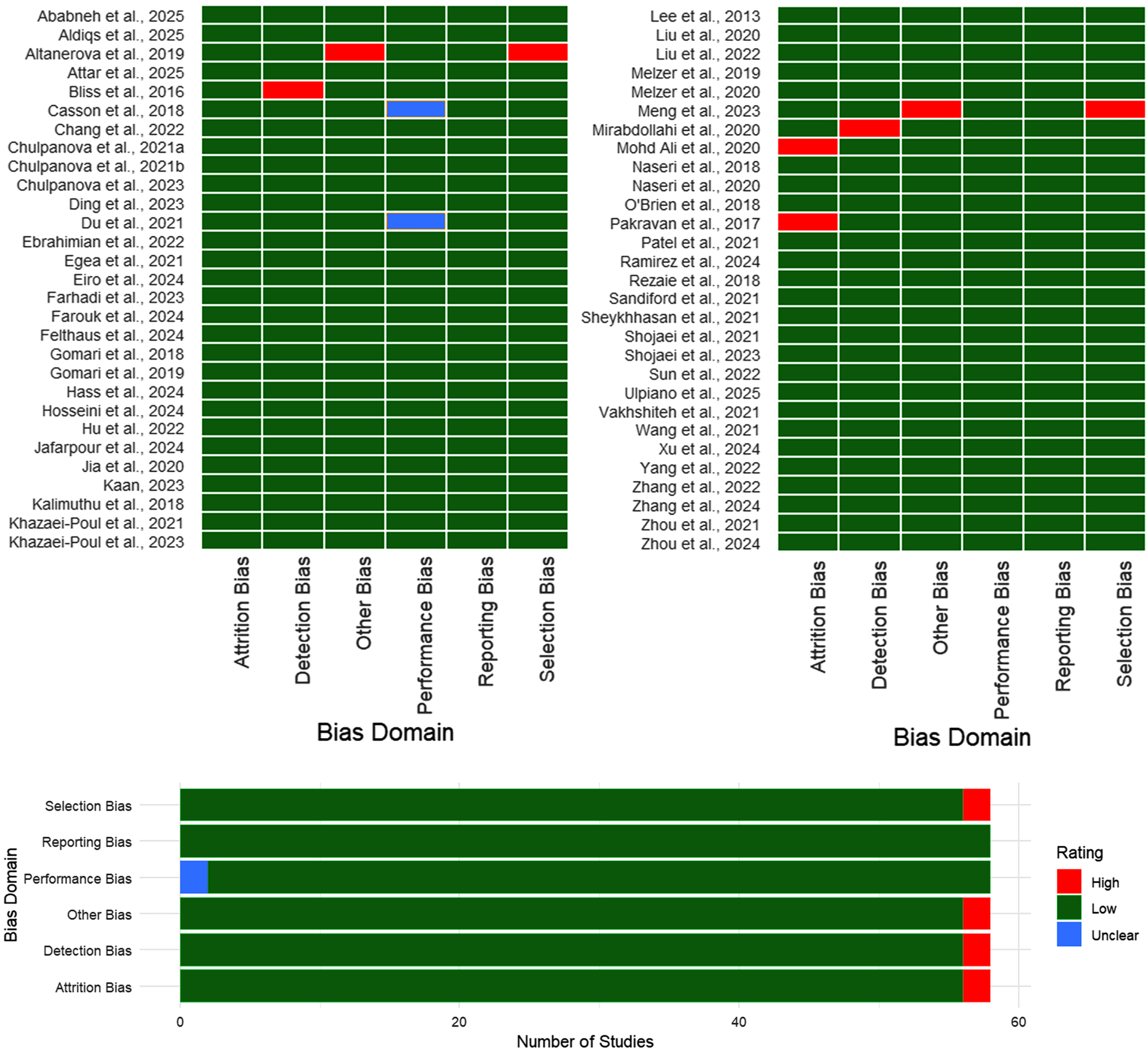

Figure 2. Risk-of-bias assessment

Summary and individual study-level evaluation of risk of bias across six domains for 58 *in vitro* studies of mesenchymal stromal/stem cell-derived extracellular vesicle (MSC-EV) therapy in breast cancer.

BT-474, TUBO, MDA-hyb1, MCF6, MCF10, and BCSC also demonstrated significant reductions in cancer cell viability following MSC-EV treatment. The test for subgroup differences across cell lines was statistically significant (χ^2 = 31.16; df = 10; p = 0.0006), indicating that the therapeutic effect of MSC-EVs may vary depending on the specific breast cancer cell type.

Subgroup analysis based on MSC source also showed consistent treatment effects across different MSC types (Figure S1). Bone marrow-derived MSCs (n = 43) yielded a pooled SMD of −4.20 (95% CI: −6.12 to −2.28), with an I^2 value of 62.2%, whereas adipose-derived MSCs (n = 51) showed a similarly strong effect with an SMD of −4.25 (95% CI: −6.28 to −2.22) and an I^2 value of 66.2%. Umbilical cord, Wharton's jelly, uterine, placental, and MSC544-EVs also demonstrated substantial inhibitory effects on cancer cell viability. However, the test for subgroup differences among MSC sources was not statistically significant (χ^2 = 13.95; df = 7;, p = 0.0590), suggesting that while all MSC sources were effective, no single MSC source was clearly superior to the others in this context.

Study or Subgroup	Experimental Mean	SD	Total	Control Mean	SD	Total	Weight	Std. Mean Difference IV, Random, 95% CI
**subgroup = Bone marrow MSC**								
Ababneh et al. 2025	45.80	4.2000	3	100.00	5.8000	3	1.8%	-8.54 [-16.46; -0.62]
Egea et al. 2022	68.50	1.0000	3	100.00	1.0000	3	0.3%	-25.13 [-48.01; -2.26]
Farouk et al. 2024	5.00	1.0000	3	90.00	2.0000	3	0.1%	-42.89 [-81.87; -3.92]
Gomari et al. 2019	5.00	1.0000	3	30.00	1.0000	3	0.4%	-19.95 [-38.13; -1.77]
Lee et al. 2013	78.10	6.3000	3	100.00	6.3000	3	4.7%	-2.77 [-5.76; 0.21]
Liu et al. 2022	51.70	0.7000	3	100.00	0.7000	3	0.1%	-55.05 [-105.07; -5.04]
Naseri et al. 2018	76.00	4.0000	3	100.00	4.0000	3	3.4%	-4.79 [-9.42; -0.16]
Naseri et al. 2018	81.80	4.6000	3	100.00	4.5000	3	4.4%	-3.19 [-6.50; 0.12]
Naseri et al. 2020	45.00	6.0000	3	98.00	5.0000	3	2.1%	-7.66 [-14.79; -0.52]
Naseri et al. 2020	94.00	7.0000	3	100.00	5.0000	3	5.7%	-0.79 [-2.54; 0.97]
Naseri et al. 2020	70.00	7.0000	3	100.00	6.0000	3	4.1%	-3.67 [-7.37; 0.03]
Pakravan et al. 2017	45.00	1.0000	3	95.00	1.0000	3	0.1%	-39.89 [-76.15; -3.64]
Sandiford et al. 2021	64.70	2.9000	4	100.00	2.3000	4	1.8%	-11.72 [-19.67; -3.76]
Vakhshiteh et al. 2021	90.00	5.0000	3	100.00	2.4000	3	5.2%	-2.03 [-4.48; 0.41]
**Total (95% CI)**			**43**			**43**	**34.3%**	**-4.20 [-6.12; -2.28]**
Heterogeneity: Tau^2 = 4.4573; Chi^2 = 34.36, df = 13 (P = 0.0011); I^2 = 62.2%								
**subgroup = Adipose MSC**								
Aldiqs et al. 2025	150.00	1.0000	3	100.00	6.0000	3	1.6%	9.28 [0.70; 17.85]
Attar et al. 2025	30.00	5.0000	3	100.00	5.0000	3	1.2%	-11.17 [-21.44; -0.90]
Attar et al. 2025	20.00	5.0000	3	100.00	5.0000	3	1.0%	-12.77 [-24.47; -1.07]
Chulpanova et al. NA	60.00	5.0000	3	90.00	5.0000	3	3.4%	-4.79 [-9.42; -0.16]
Chulpanova et al. NA	40.00	5.0000	3	90.00	5.0000	3	2.0%	-7.98 [-15.40; -0.56]
Ebrahimian et al. 2022	2.00	1.0000	3	20.00	5.0000	3	3.9%	-3.98 [-7.94; -0.03]
Felthaus et al. 2024	106.70	11.7000	3	100.00	10.0000	3	5.8%	0.49 [-1.17; 2.15]
Jafarpour et al. 2024	30.00	6.0000	3	100.00	2.0000	3	1.0%	-12.49 [-23.94; -1.04]
Jafarpour et al. 2024	25.00	4.0000	3	100.00	2.0000	3	0.5%	-18.92 [-36.18; -1.67]
Kaan et al. 2023	45.00	1.0000	3	100.00	1.0000	3	0.1%	-43.88 [-83.76; -4.01]
Mohd Ali et al. 2020	80.00	4.0000	3	100.00	5.0000	3	4.2%	-3.52 [-7.10; 0.05]
Mohd Ali et al. 2021	85.00	6.0000	3	100.00	6.0000	3	5.2%	-1.99 [-4.41; 0.42]
Ramirez et al. 2024	45.00	8.0000	3	100.00	10.0000	3	3.4%	-4.85 [-9.53; -0.16]
Rezaie et al. 2018	50.00	4.2000	3	100.00	5.0000	3	1.8%	-8.64 [-16.65; -0.63]
Shojaei et al. 2021	57.10	8.6000	3	100.00	11.4000	3	4.3%	-3.39 [-6.86; 0.08]
Shojaei et al. 2023	66.70	1.4000	3	100.00	1.4000	3	0.5%	-18.98 [-36.28; -1.67]
Zhang et al. 2024	20.00	4.0000	3	35.00	1.0000	3	3.8%	-4.11 [-8.16; -0.05]
**Total (95% CI)**			**51**			**51**	**43.6%**	**-4.25 [-6.28; -2.22]**
Heterogeneity: Tau^2 = 8.0244; Chi^2 = 47.39, df = 16 (P < 0.0001); I^2 = 66.2%								
**subgroup = MSCs**								
Altanerova et al. 2019	10.00	5.0000	3	100.00	5.0000	3	0.8%	-14.36 [-27.50; -1.22]
Gomari et al. 2018	80.00	7.0000	3	100.00	1.0000	3	4.4%	-3.19 [-6.50; 0.12]
**Total (95% CI)**			**6**			**6**	**5.2%**	**-6.89 [-17.20; 3.41]**
Heterogeneity: Tau^2 = 38.4967; Chi^2 = 2.61, df = 1 (P = 0.1061); I^2 = 61.7%								
**subgroup = Uterine MSC**								
Eiro et al. 2024	15.00	10.0000	3	76.00	5.0000	3	2.7%	-6.16 [-11.97; -0.34]
**subgroup = Umbilical cord MSC**								
Farhadi et al. 2023	40.90	9.1000	3	100.00	18.2000	3	4.4%	-3.28 [-6.66; 0.10]
Hass et al. 2024	80.00	4.0000	3	100.00	6.0000	3	4.5%	-3.13 [-6.39; 0.13]
Khazaei-Poul et al. 2021	52.20	3.5000	3	100.00	4.4000	3	1.5%	-9.59 [-18.45; -0.74]
Melzer et al. 2019	10.00	2.0000	3	100.00	5.0000	3	0.5%	-18.86 [-36.06; -1.66]
Patel et al. 2021	5.00	1.0000	3	95.00	3.0000	3	0.2%	-32.11 [-61.32; -2.91]
**Total (95% CI)**			**15**			**15**	**11.1%**	**-4.05 [-6.29; -1.81]**
Heterogeneity: Tau^2 = < 0.0001; Chi^2 = 8.41, df = 4 (P = 0.0777); I^2 = 52.4%								
**subgroup = Wharton's jelly MSCs**								
Hosseini et al. 2024	50.00	6.0000	3	97.00	2.0000	3	1.8%	-8.39 [-16.17; -0.61]
Mirabdollahi et al. 2020	10.00	3.0000	3	100.00	2.0000	3	0.2%	-28.17 [-53.79; -2.54]
Mirabdollahi et al. 2020	20.00	3.0000	3	100.00	2.0000	3	0.3%	-25.04 [-47.82; -2.25]
**Total (95% CI)**			**9**			**9**	**2.3%**	**-16.45 [-30.40; -2.50]**
Heterogeneity: Tau^2 = 75.6415; Chi^2 = 3.6, df = 2 (P = 0.1656); I^2 = 44.4%								
**subgroup = MSC544**								
Melzer et al. 2020	15.00	2.0000	3	100.00	5.0000	3	0.5%	-17.81 [-34.06; -1.56]
**subgroup = Placental MSC**								
Xu et al. 2024	16.70	1.7000	3	100.00	1.7000	3	0.1%	-39.10 [-74.63; -3.56]
Xu et al. 2024	21.40	1.4000	3	100.00	1.4000	3	0.1%	-44.80 [-85.50; -4.09]
**Total (95% CI)**			**6**			**6**	**0.2%**	**-41.56 [-68.33; -14.79]**
Heterogeneity: Tau^2 = 0; Chi^2 = 0.04, df = 1 (P = 0.8362); I^2 = 0%								
**Total (95% CI)**			**136**			**136**	**100.0%**	**-4.79 [-6.03; -3.54]**
**Prediction interval**								**[-9.97; 0.40]**

Heterogeneity: Tau^2 = 6.2154; Chi^2 = 117.42, df = 44 (P < 0.0001); I^2 = 62.5%
Test for overall effect: Z = -7.54 (P < 0.0001)
Test for subgroup differences: Chi^2 = 13.59, df = 7 (P = 0.0590)

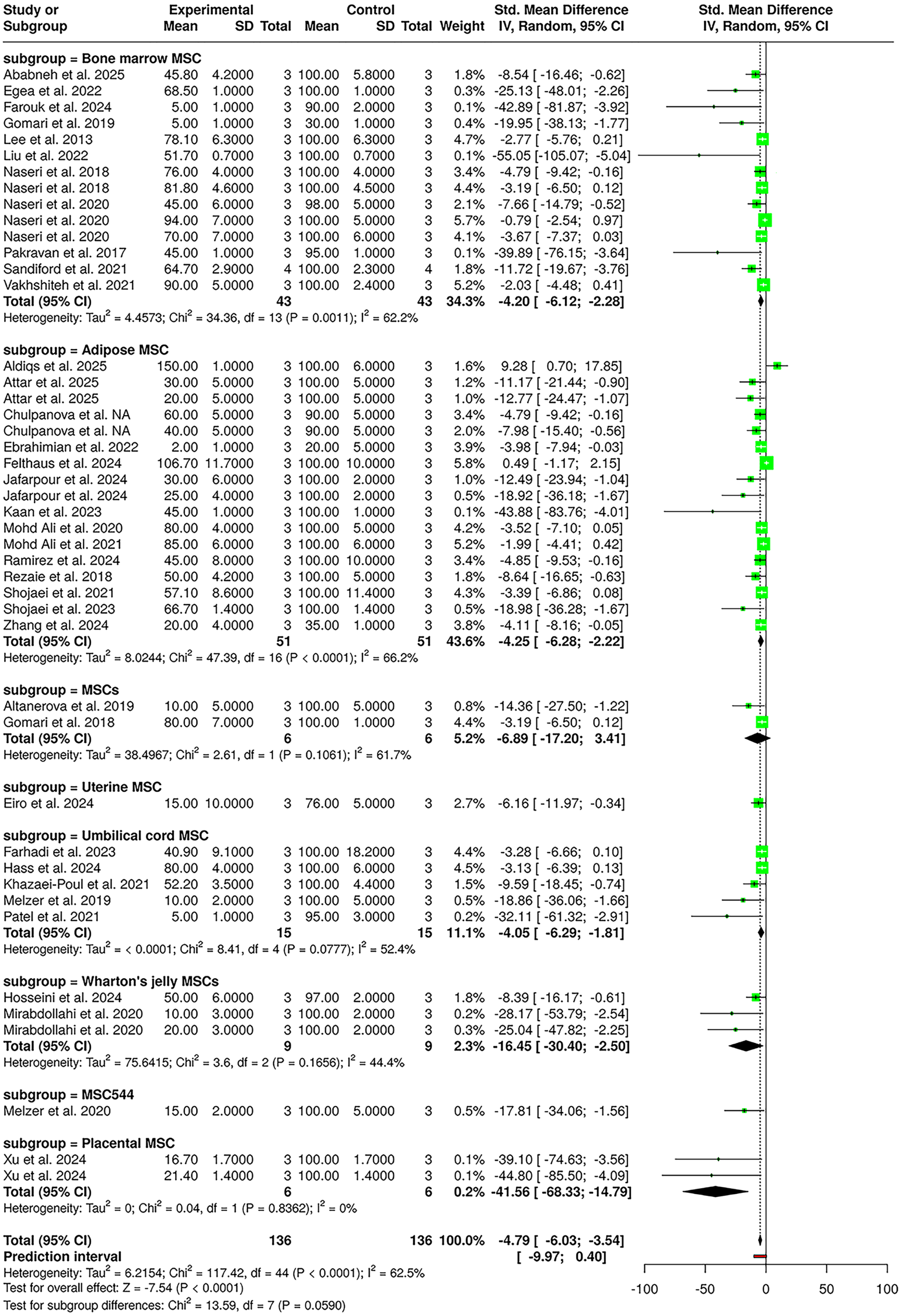

Figure 3. Effect of MSC-EV therapy on cell viability

Forest plot showing standardized mean differences (SMDs) in breast cancer cell viability after MSC-EV treatment, stratified by MSC source.

In summary, this meta-analysis supports a strong and consistent anticancer effect of MSC-EVs across a wide range of breast cancer cell types and MSC sources. Although significant heterogeneity was observed, subgroup analyses helped elucidate patterns of variation and confirmed the robustness of the overall findings.

Moderate heterogeneity observed across the analyses (I^2 = 50%–70%) likely arises from methodological variations among the studies. Factors contributing to this variability include differences in assay sensitivity, EV isolation and characterization techniques (ultracentrifugation, precipitation kits, or size-exclusion chromatography), and the use of native versus engineered or drug-loaded MSC-EVs. Additionally, heterogeneity in the MSC tissue source, culture conditions, and breast cancer cell line responsiveness may have influenced effect magnitude. These methodological discrepancies underline the need for standardized EV preparation and reporting protocols to enhance reproducibility and enable more homogeneous future meta-analyses.

A sensitivity analysis excluding studies using noncomparable assay formats (n = 8) confirmed the robustness of pooled estimates. The effect size for viability changed marginally (SMD = −4.52 [95% CI: –5.87, 3.18]; I^2 = 58%), supporting the consistency of our findings despite methodological variation. Subgroup analysis by EV dose could not be performed because fewer than 20% of studies reported standardized dosage information.

Apoptosis

In total, 32 studies were included in the meta-analysis, comprising 108 subjects in the experimental cohort and 108 in the control cohort. Using a random-effects model with the inverse variance method, the analysis demonstrated a statistically significant difference between the MSC-EV-treated and control groups in terms of enhanced cancer cell apoptosis. The summarized SMD was 4.16, with a 95% CI of 2.75–5.56, indicating a strong overall effect in favor of the experimental group. The test for the overall effect was significant ($p < 0.05$).

Significant heterogeneity was observed across the studies ($p < 0.01$), with an I^2 value of 64.6%, suggesting that the majority of variability in treatment outcomes stems from true heterogeneity in study characteristics, rather than sampling error.

Subgroup analysis by the breast cancer cell line (Figure S2) revealed the highest effects among several cell types. For MCF7 cells, the pooled SMD was 4.61 (95% CI: 1.53–7.69), showing considerable enhancement in apoptosis ability upon MSC-EV exposure, with an I^2 value of 62.5%. MDA-MB-231 cell studies showed a similarly high pooled effect size of 4.57 (95% CI: 2.38–6.76), with moderate heterogeneity (I^2 = 64.3%). Umbilical cord MSC-EVs had particularly strong effects in studies involving MDA-MB-231 and MCF7 lines. Other cell lines including BT-474, MDA-MB-468, SK-BR-3, and BCSC also showed positive effects, though the magnitude varied.

A second subgroup analysis based on the source of MSCs (Figure 4) demonstrated that bone marrow MSC-EVs (n = 37) had a pooled SMD of 2.34 (95% CI: 0.21–4.89], showing a moderate but significant effect. Adipose-derived MSCs, contributing the largest number of studies (n = 44), had a pooled SMD of 3.78 (95% CI: 2.49–5.06), supporting a strong effect with relatively low heterogeneity (I^2 = 53.4%). Wharton's jelly MSCs showed the highest individual effect size, with an SMD of 22.48 (95% CI: 2.01–42.95) in one study. Other sources including uterine and umbilical cord MSCs also contributed meaningfully, with the umbilical cord MSC subgroup showing the most robust pooled effect size of 6.49 (95% CI: 2.62–10.36), though with moderate heterogeneity.

The test for subgroup differences by cancer cell line was statistically significant (χ^2 = 34.90, df = 10, p = 0.0001), indicating that the cell type plays a significant role in MSC-EV-mediated effects. In contrast, no significant subgroup difference was found across MSC sources (χ^2 = 8.54, df = 5, p = 0.1291), suggesting a broadly similar impact across different MSC origins.

Migration

A total of 21 studies were analyzed, encompassing 72 subjects in the experimental cohort and 72 subjects in the control cohort, to evaluate the effects of MSC-EVs on breast cancer cell migration. The analysis used a random-effects model with the inverse variance method to compare SMDs between the treatment and control groups.

The pooled analysis showed a statistically significant reduction in cell migration in the MSC-EV-treated groups, with a summarized SMD of −4.70 and a 95% CI of −6.54 to −2.85. The test for the overall effect confirmed significance ($p < 0.05$). A substantial heterogeneity was detected ($p < 0.01$), with an I^2 value of 67.4%, indicating that 67% of the variability across studies was due to real differences in effect sizes, rather than sampling error.

Subgroup analysis by the breast cancer cell line revealed variable responses (Figure S3). In the MCF7 subgroup (7 studies), MSC-EVs significantly suppressed migration with a pooled SMD of −4.32 (95% CI: −7.88, −0.76), although heterogeneity was high (I^2 = 73.3%). The MDA-MB-231 subgroup (12 studies) showed a similarly strong pooled effect of −5.17 (95% CI: −7.97, −2.37), with an I^2 value of 69.2%. The 4T1 subgroup (4 studies) exhibited the most pronounced inhibitory effect, with an SMD of −6.82 (95% CI: −11.41, −2.23). The SK-BR-3 subgroup (1 study) showed a nonsignificant reduction in migration (SMD = −0.80 [95% CI: −2.55, 0.96]). The test for subgroup differences by cell line was statistically significant (χ^2 = 11.83, p = 0.0098), indicating that the MSC-EV impact varied by the cancer cell type.

Further subgroup analysis based on the MSC source demonstrated notable differences (Figure 5). Bone marrow MSCs (9 studies) showed a strong pooled SMD of −5.48 (95% CI: −8.75, −2.21), with moderate heterogeneity (I^2 = 61.1%). Adipose MSCs (6 studies)

Study or Subgroup	Experimental Mean	SD	Total	Control Mean	SD	Total	Weight	Std. Mean Difference IV, Random, 95% CI
**subgroup = Bone marrow MSC**								
Ababneh et al. 2025	10.00	1.0000	3	10.00	1.0000	3	5.7%	0.00 [-1.60; 1.60]
Liu et al. 2022	25.00	3.0000	3	5.00	1.2000	3	2.6%	6.98 [0.44; 13.52]
Meng et al. 2023	40.00	4.0000	3	1.00	0.5000	3	1.5%	10.92 [0.88; 20.96]
Naseri et al. 2018	6.00	0.1000	3	0.00	0.0000	3	0.0%	
Naseri et al. 2018	6.00	0.1000	3	0.00	0.0000	3	0.0%	
Naseri et al. 2020	7.00	1.0000	3	1.00	1.0000	3	3.7%	4.79 [0.16; 9.42]
Naseri et al. 2020	1.00	1.0000	3	1.00	1.0000	3	5.7%	0.00 [-1.60; 1.60]
Naseri et al. 2020	3.00	1.5000	3	2.00	1.5000	3	5.7%	0.53 [-1.14; 2.20]
Sandiford et al. 2021	4.00	1.0000	4	13.00	2.0000	4	4.4%	-4.94 [-8.53; -1.36]
Vakhshiteh et al. 2021	24.00	3.0000	3	1.00	0.5000	3	2.1%	8.53 [0.62; 16.44]
Zhang et al. 2022	8.00	0.6000	3	3.80	0.4000	3	2.8%	6.57 [0.39; 12.75]
Zhang et al. 2022	6.30	0.4000	3	5.00	0.4000	3	4.9%	2.59 [-0.25; 5.44]
**Total (95% CI)**			**37**			**37**	**39.1%**	**2.34 [-0.21; 4.89]**
Heterogeneity: $Tau^2 = 11.7528$; $Chi^2 = 30.46$, df = 9 (P = 0.0004); $I^2 = 70.5\%$								
**subgroup = Adipose MSC**								
Aldiqs et al. 2025	40.00	2.5000	3	30.00	2.5000	3	4.6%	3.19 [-0.12; 6.50]
Attar et al. 2025	90.00	5.0000	3	10.00	2.0000	3	0.7%	16.76 [1.46; 32.07]
Attar et al. 2025	85.00	5.0000	3	5.00	1.0000	3	0.7%	17.70 [1.55; 33.86]
Chulpanova et al. 2023	60.00	5.0000	3	20.00	5.0000	3	2.9%	6.38 [0.37; 12.40]
Chulpanova et al. NA	50.00	5.0000	4	20.00	5.0000	4	4.3%	5.21 [1.46; 8.96]
Chulpanova et al. NA	20.00	5.0000	4	10.00	5.0000	4	5.6%	1.74 [-0.07; 3.55]
Ding et al. 2023	40.00	5.0000	3	5.00	2.0000	3	2.5%	7.33 [0.49; 14.18]
Jafarpour et al. 2024	82.00	4.0000	3	20.00	3.0000	3	1.0%	13.99 [1.19; 26.80]
Jafarpour et al. 2024	78.00	4.0000	3	10.00	2.0000	3	0.7%	17.16 [1.50; 32.82]
Jia et al. 2020	38.00	3.0000	3	22.00	4.0000	3	4.4%	3.61 [-0.04; 7.26]
Jia et al. 2020	36.00	4.0000	3	18.00	4.0000	3	4.4%	3.59 [-0.04; 7.22]
Kaan et al. 2023	4.50	0.0000	3	1.00	0.0000	3	0.0%	
Shojaei et al. 2021	16.00	0.8000	3	13.00	0.8000	3	4.7%	2.99 [-0.16; 6.15]
Shojaei et al. 2023	15.00	1.5000	3	9.00	1.0000	3	4.3%	3.76 [-0.01; 7.52]
**Total (95% CI)**			**44**			**44**	**40.7%**	**3.78 [2.49; 5.06]**
Heterogeneity: $Tau^2 = 0.8186$; $Chi^2 = 18.03$, df = 12 (P = 0.1149); $I^2 = 33.4\%$								
**subgroup = MSCs**								
Altanerova et al. 2019	50.00	8.0000	3	10.00	3.0000	3	3.4%	5.28 [0.23; 10.34]
**subgroup = Uterine MSC**								
Eiro et al. 2024	33.00	3.0000	3	2.00	1.0000	3	1.5%	11.06 [0.89; 21.23]
**subgroup = Umbilical cord MSC**								
Farhadi et al. 2023	18.00	7.0000	3	5.00	2.0000	3	5.2%	2.01 [-0.42; 4.45]
Khazaei-Poul et al. 2021	18.00	0.8000	3	13.00	0.8000	3	3.6%	4.99 [0.18; 9.79]
Wang et al. 2021	18.00	1.0000	3	6.00	1.0000	3	1.8%	9.57 [0.74; 18.41]
Wang et al. 2021	18.00	1.0000	3	6.00	1.0000	3	1.8%	9.57 [0.74; 18.41]
Yang et al. 2022	25.00	2.0000	3	3.00	0.5000	3	1.3%	12.04 [0.99; 23.09]
Yang et al. 2022	20.00	1.5000	3	3.00	0.5000	3	1.3%	12.13 [1.00; 23.26]
**Total (95% CI)**			**18**			**18**	**14.9%**	**6.49 [2.62; 10.36]**
Heterogeneity: $Tau^2 = 10.4699$; $Chi^2 = 9.86$, df = 5 (P = 0.0793); $I^2 = 49.3\%$								
**subgroup = Wharton's jelly MSCs**								
Hosseini et al. 2024	67.00	3.0000	3	4.00	1.0000	3	0.4%	22.48 [2.01; 42.95]
**Total (95% CI)**			**108**			**108**	**100.0%**	**4.16 [2.75; 5.56]**
**Prediction interval**								**[-1.91; 10.23]**
Heterogeneity: $Tau^2 = 8.3396$; $Chi^2 = 87.55$, df = 31 (P < 0.0001); $I^2 = 64.6\%$								
Test for overall effect: Z = 5.80 (P < 0.0001)								
Test for subgroup differences: $Chi^2 = 8.54$, df = 5 (P = 0.1291)								

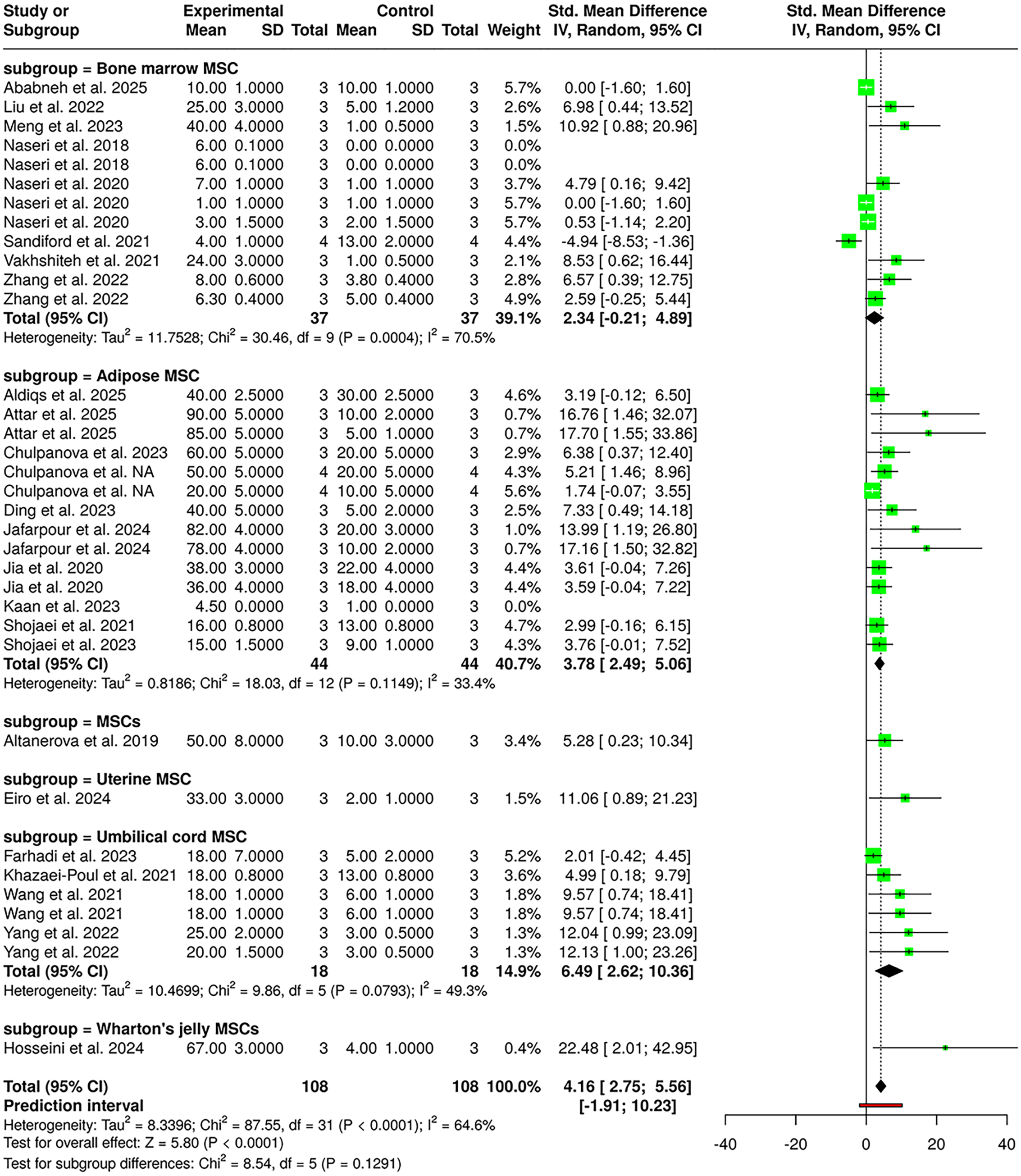

Figure 4. Effect of MSC-EV therapy on apoptosis

Forest plot displaying standardized mean differences (SMDs) for breast cancer cell apoptosis following MSC-EV treatment, stratified by MSC source.

had a pooled SMD of −5.33 (95% CI: −7.17, −0.49) but with higher heterogeneity ($I^2 = 77.6\%$). Notably, Wharton's jelly MSCs demonstrated the greatest individual effect size, with a pooled SMD of −6.09 (95% CI: −24.12, 11.94), though the wide CI reflects low study number and uncertainty. Umbilical cord MSCs (6 studies) showed a pooled SMD of −5.48 (95% CI: −8.32, −2.65), while uterine and placental MSCs contributed isolated studies with SMDs of −3.32 and −3.53, respectively. The test for subgroup differences by the MSC source was not statistically significant ($p = 0.8978$), suggesting that all sources effectively suppressed migration, without one clearly outperforming the others.

Overall, the data support a robust inhibitory effect of MSC-EVs on breast cancer cell migration, consistent across most MSC sources

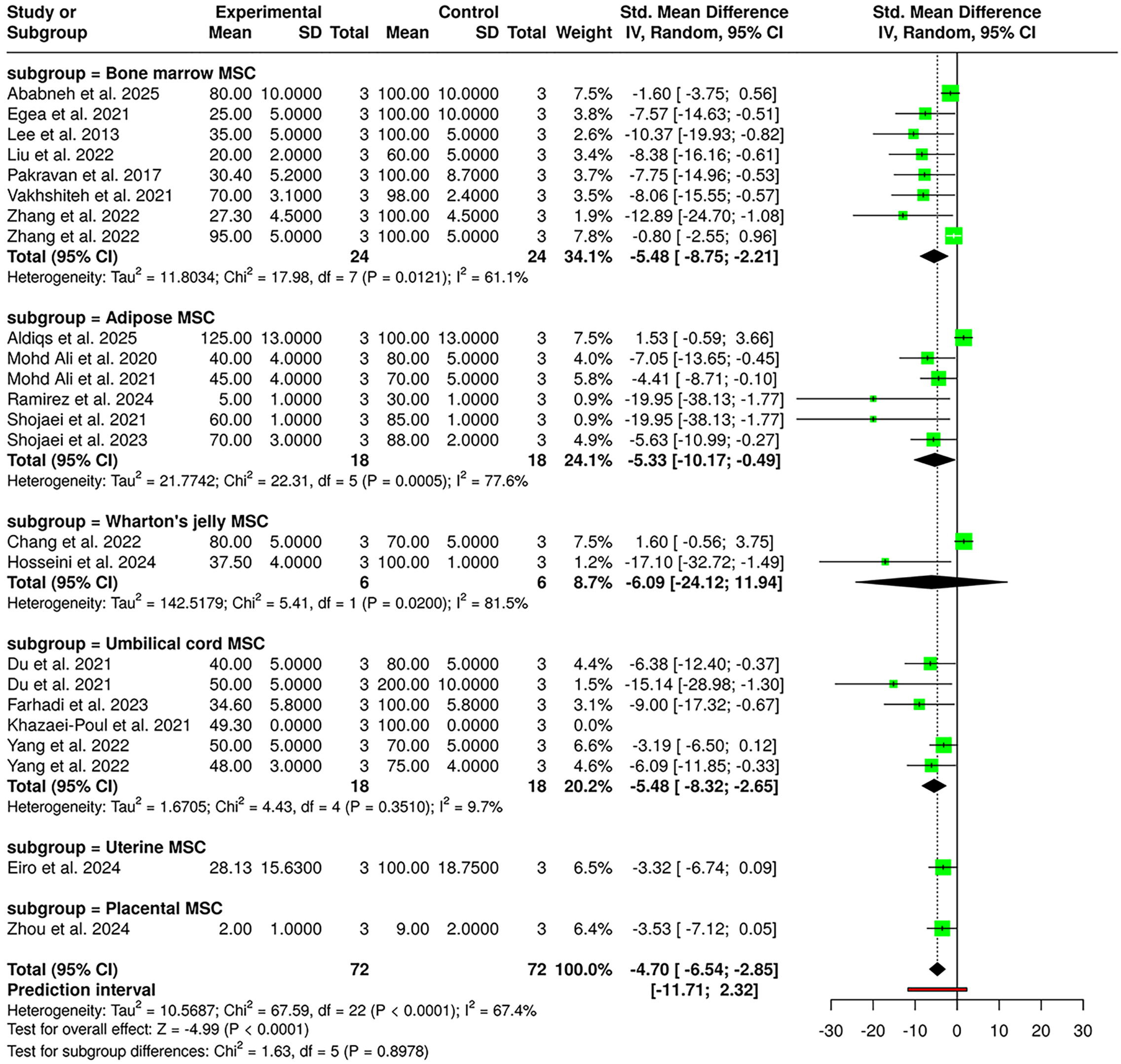

Figure 5. Effect of MSC-EV therapy on cell migration

Forest plot presenting standardized mean differences (SMDs) for breast cancer cell migration following MSC-EV treatment, stratified by MSC source.

and cancer cell lines, though the magnitude of effect and study precision varied. Funnel plots and Egger's tests revealed no significant asymmetry for any outcome ($p > 0.05$; Figure S4), suggesting limited publication bias.

Geographic distribution of studies

Most studies originated from China and Iran, followed by Germany and the United States (Table 2; Figure S5).

DISCUSSION

This systematic review provides a mechanistic map of MSC-EV-cancer interactions derived from controlled *in vitro* systems, rather than direct translational evidence. The pooled quantitative results reflect comparative mechanistic trends, rather than clinical efficacy estimates. Our meta-analysis demonstrated statistically significant reductions in viability and migration and increased apoptosis following MSC-EV treatment across multiple breast cancer models.

Dualistic effects of MSC-EVs: Therapeutic promise versus tumor dormancy

While the pooled analyses clearly demonstrate the strong anticancer efficacy of MSC-EVs—marked by reduced viability, enhanced apoptosis, and suppressed migration—emerging evidence introduces a paradoxical aspect to their biological behavior. Certain unmodified MSC-EVs, particularly those derived from bone marrow sources, may inadvertently promote tumor dormancy or therapy resistance by transferring specific miRNAs and signaling molecules.

Table 3. Characteristics of *in vitro* studies on mesenchymal stem cell-derived extracellular vesicles that had pro-tumorigenic potential

Citation	Study focus	Key findings	Mechanisms/pathways involved	Experimental models
Almouh et al. (2024)[67]	role of EVs from oxidative stress-induced MSCs in murine mammary tumor progression	EVs from H_2O_2-treated MSCs (St-MSC Exo) promote breast cancer cell progression, VEGF expression, and angiogenesis. Untreated MSC-EVs reduce tumor progression.	STAT3 activation, NF-κB activation, increased ROS production, VEGF induction, and epithelial-to-mesenchymal transition (EMT)	*in vitro*: 4T1 breast cancer cells; *in vivo*: murine mammary tumor model
Chen et al. (2023)[68]	effect of BMSC-derived EVs on breast cancer via Hedgehog signaling	BMSC-derived EVs enhance MCF-7 cell proliferation and migration by upregulating Hedgehog signaling components (Gli1, SMO).	Hedgehog signaling pathway, *p*-Akt, β-catenin expression	*in vitro*: MCF-7 breast cancer cells
Lin et al. (2013)[69]	role of human adipose-derived MSC-EVs in breast cancer cell migration	adipose MSC-derived EVs promote MCF-7 cell migration by activating Wnt signaling.	Wnt signaling pathway	*in vitro*: MCF-7 breast cancer cells
Liu et al. (2021).[70]	role of hypoxic BMSC-EVs containing miR-328-3p in lung cancer progression	hypoxic BMSC-EVs deliver miR-328-3p, promoting lung cancer cell proliferation, invasion, migration, and EMT. High miR-328-3p in patient serum.	NF2-mediated Hippo pathway inhibition	*in vitro*: lung cancer cells; *in vivo*: xenograft nude mice; clinical: serum analysis of lung cancer patients
Movahed et al. (2025)[71]	effect of BM-MSCs on breast cancer stem cell (CSC) enrichment via metabolic pathways	BM-MSC-EVs and conditioned media increase CSC markers (NANOG, OCT-4, and CD44) and alter glycolysis, pentose phosphate pathway, and amino acid metabolism, promoting tumor growth.	metabolic reprogramming (glycolysis, pentose phosphate pathway, and amino acid metabolism)	*in vitro*: MCF-7 and MDA-MB-231 cells; *in vivo*: 4T1 mouse model
Orso et al. (2023)[72]	role of stroma-derived miR-214 in tumor dissemination	stroma-derived miR-214 in EVs promotes breast cancer cell migration, invasion, and metastasis; tumor cells induce miR-214 in stroma via IL-6/STAT3.	IL-6/STAT3 signaling and miR-214-mediated metastatic program	*in vitro*: breast cancer cells; *in vivo*: miR-214 overexpressing/knockout mice
Vallabhaneni et al. (2015)[73]	characterization of EV cargo from serum-deprived BM-MSCs in breast cancer	BM-MSC-EVs contain tumor-supportive miRNAs (miR-21 and miR-34a), proteins (PDGFR-β and TIMP-1/2), lipids, and metabolites, promoting MCF-7 tumor growth.	miR-21, miR-34a, bioactive lipids, lactic acid, and glutamic acid	*in vitro*: MCF-7 cells; *in vivo*: co-injection xenograft model
Vallabhaneni et al. (2017)[74]	role of BM-MSC-EVs in breast cancer metastasis regulation	BM-MSC-EVs suppress metastasis in parental MDA-MB-231 cells but not organotropic sublines via miR-205 and miR-31, targeting UBE2N/Ubc13.	UBE2N/Ubc13 pathway, miR-205, and miR-31	*in vitro*: MDA-MB-231 parental and organotropic sublines
Wang et al. (2025)[75]	role of hypoxic BMSC-derived exosomal miR-210-3p in TNBC	hypoxic BMSC-EVs deliver miR-210-3p, promoting TNBC proliferation, migration, invasion, and EMT by targeting NFIX and activating Wnt/β-catenin signaling.	NFIX-Wnt/β-catenin signaling axis	*in vitro*: TNBC cells; *in vivo*: xenograft nude mice; clinical: serum analysis of TNBC patients
Wang et al. (2019)[76]	effect of MSC-differentiated adipocyte EVs on breast cancer	adipocyte-EVs promote MCF-7 proliferation, migration, and protect against apoptosis by activating Hippo signaling (YAP/TAZ).	Hippo signaling pathway (YAP/TAZ)	*in vitro*: MCF-7 cells; *in vivo*: mouse xenograft model
Worner et al. (2019)[77]	transformation of naive MSCs by breast cancer microenvironment	naive MSCs exposed to MDA-MB-231 conditioned media or EVs form tumor-like masses, with DNA hypermethylation driving migration and crosstalk.	DNA hypermethylation and tumor-stroma crosstalk	*in vitro*: MDA-MB-231 cells; *in vivo*: nude mice mammary gland injection
Wu et al. (2022a)[78]	effect of BM-MSC-EVs on breast cancer proliferation and migration	BM-MSC-EVs promote MDA-MB-231 proliferation and migration via upregulation of YAP/TAZ in the Hippo pathway.	Hippo signaling (YAP/TAZ)	*in vitro*: MDA-MB-231 cells
Wu et al. (2022b)[79]	role of BMSC-derived miR-206 in breast cancer development	BMSC-derived exosomal miR-206 promotes breast cancer cell invasion and proliferation by targeting Rab23 and activating Hedgehog signaling.	Hedgehog signaling; Rab23 regulation	*in vitro*: breast cancer cells
Xing et al. (2020)[80]	role of lncRNA HAND2-AS1 in suppressing TNBC via MSC-derived EVs	LncRNA HAND2-AS1 reduces exosomal miR-106a-5p secretion from MSCs, inhibiting TNBC progression.	inhibition of miR-106a-5p; tumor suppression	*in vitro*: TNBC cells; *in vivo*: TNBC model
Yang et al. (2015)[81]	acquisition of tumor cell properties via MSC-EVs	MSC-EVs transfer MMP-2 and MSC markers, enabling MCF-7 and SCCOHT-1 cells to acquire gelatinase activity and new metabolic functions.	MMP-2 activity, EMT, and tumor microenvironment remodeling	*in vitro*: MCF-7 and SCCOHT-1 cells
Zakiyah et al. (2023)[82]	effect of MSC-EVs on MCF-7 stemness and proliferation	MSC-EVs increase MCF-7 proliferation and stemness (OCT4, ALDH1) in a concentration-dependent manner.	OCT4 expression; ALDH1 activity	*in vitro*: MCF-7 cells

(*Continued on next page*)

Table 3. *Continued*

Citation	Study focus	Key findings	Mechanisms/pathways involved	Experimental models
Zhou et al. (2019)[83]	role of hUC-MSC-EVs in breast cancer progression	hUC-MSC-EVs enhance MDA-MB-231 and MCF-7 proliferation, migration, and invasion via ERK-mediated EMT.	ERK pathway; EMT	*in vitro*: MDA-MB-231 and MCF-7 cells
Zhu et al. (2024)[84]	role of adipose stem cell EVs in TNBC immune evasion	pro-inflammatory cytokine-stimulated adipose MSC-EVs promote TNBC immune evasion via UCHL1-mediated HDAC6/STAT3/PD-L1 signaling.	HDAC6/STAT3/PD-L1 pathway; UCHL1 regulation	*in vitro*: TNBC cells, macrophages, and T cells; *in vivo*: TNBC xenograft mice

ALDH1, aldehyde dehydrogenase 1; Akt, protein kinase B; ASCs, adipose-derived stem cells; β-catenin, a transcriptional co-activator in the canonical Wnt signaling pathway; BM-MSCs, bone marrow-derived mesenchymal stem cells; BMSCs, bone marrow stromal/stem cells; CD44, cluster of differentiation 44 cell surface glycoprotein; CSC, cancer stem cell; EMT, epithelial-to-mesenchymal transition; ERK, extracellular signal-regulated kinase; EVs, extracellular vesicles; Gli1, glioma-associated oncogene homolog 1; H_2O_2, hydrogen peroxide; HAND2-AS1, heart and neural crest derivatives expressed 2 antisense RNA 1; HDAC6, histone deacetylase 6; Hippo, hippo signaling pathway; hUC-MSCs, human umbilical cord-derived mesenchymal stem cells; IL-6, interleukin-6; lncRNA, long non-coding RNA; MCF-7, human estrogen receptor-positive breast cancer cell line; MDA-MB-231, human triple-negative breast cancer cell line; miR, MicroRNA; MMP-2, matrix metalloproteinase-2; MSC-EVs, mesenchymal stem cell-derived extracellular vesicles; NANOG, homeobox transcription factor associated with pluripotency; NF2, neurofibromin 2 (Merlin); NF-κB, nuclear factor kappa-light-chain-enhancer of activated B cells; NFIX, nuclear factor I X; OCT-4, octamer-binding transcription factor 4; PD-L1, programmed death-ligand 1; PDGFR-β, platelet-derived growth factor receptor beta; p-Akt, phosphorylated Akt; Rab23, Ras-related protein Rab-23; ROS, reactive oxygen species; SCCOHT-1, small cell carcinoma of the ovary hypercalcemic type-1 cell line; SMO, smoothened receptor; STAT3, signal transducer and activator of transcription 3; St-MSC Exo, stress-treated mesenchymal stem cell-derived exosomes; TAZ, transcriptional co-activator with PDZ-binding motif; TIMP-1/2, tissue inhibitors of metalloproteinases-1 and -2; TNBC, triple-negative breast cancer; UBE2N (Ubc13), ubiquitin-conjugating enzyme E2 N; UCHL1, ubiquitin carboxyl-terminal hydrolase L1; VEGF, vascular endothelial growth factor; Wnt, wingless-related integration site signaling pathway; YAP, yes-associated protein.

The following section explores this dualistic phenomenon, examining both the mechanistic basis and clinical implications of dormancy induction by MSC-EVs. Emerging data highlight a contrasting pro-tumorigenic potential, especially in unmodified bone marrow-derived MSC-EVs (Table 3).

A landmark study by Ono et al.[85] showed that exosomal miR-23b, transferred from bone marrow-derived MSCs to metastatic BM2 breast cancer cells, induced dormancy with reduced proliferation, invasion, and chemotherapy sensitivity by targeting MARCKS. Dormant tumor cells can evade therapy and linger in bone marrow niches, only to re-emerge years later—posing a significant relapse risk.[86] Similarly, MSC-EVs enriched in miR-21-5p have been shown to confer doxorubicin resistance via S100A6 upregulation.[87] These findings suggest that unmodified EVs from certain MSC sources may inadvertently shield cancer cells, underscoring the need for targeted engineering.

Applied engineering: Shifting MSC-EVs toward therapy

Engineered MSC-EVs loaded with tumor-suppressive molecules (miRNAs or chemotherapeutics) demonstrate enhanced anticancer efficacy compared with native EVs.[88,89] Surface modification further enhances tumor targeting and minimizes off-target effects. Integrating targeting ligands such as cRGD peptides or LAMP2b-DARPin fusions onto EV membranes improves delivery specificity. This modification enhances uptake by tumor cells and increases the therapeutic payload at the tumor site.[90]

Thirdly, culture source revision can influence EV cargo composition. EVs derived from placenta or Wharton's jelly MSCs appear less likely to harbor dormancy-inducing miRNAs—such as miR-23b and miR-21-5p—compared with bone marrow-derived MSCs.[91] This suggests that selecting an optimal MSC source is a strategic step toward minimizing pro-tumor risks.

Thus, practical development of MSC-EV-based therapies should integrate three key applied strategies—active loading of therapeutic cargo (such as miR-16, miR-379, and chemotherapeutics), surface engineering of the EVs with ligands such as cRGD or DARPin, and use of MSC sources less prone to dormancy signals. These tactics will convert MSC-EVs from passive biovectors into highly controlled, precision cancer therapies, maximizing antitumor efficacy while minimizing unintended dormancy or chemoresistance.

While these *in vitro* results reveal consistent mechanistic patterns, translating MSC-EVs into clinical products remains challenging. Large-scale, GMP-compliant production requires standardized isolation, potency assays, and inter-batch quality control. Variability in culture conditions, EV yield, and bioactivity continues to hinder reproducibility and regulatory approval.

Clinical relevance: Navigating dormancy risk

In vivo findings indicate that bone marrow-derived MSC-EVs can induce dormancy through miR-23b transfer, highlighting the importance of excluding dormancy-associated cargo in clinical development.[85] To mitigate dormancy risk, MSC-EV-based therapies should undergo rigorous cargo profiling, targeted delivery optimization, and longitudinal preclinical monitoring for reactivation or recurrence.

Strengths and limitations

This review's PRISMA-compliant design and quantitative synthesis provide transparent and reproducible evidence of MSC-EVs' antitumor activity. However, heterogeneity across the studies and limited *in vivo* validation restrict direct translational conclusions. Inconsistent or missing EV dose reporting across studies prevented the assessment of dose-response effects, which may also influence therapeutic efficacy.

Future directions

Advancing MSC-EVs safely into clinical use requires a multifaceted approach. First, rigorous cargo profiling using next-generation sequencing and proteomics should be standard practice to confirm the absence of dormancy-encoding miRNAs like miR-23b and miR-21-5p. Recent reviews in cell and molecular biology emphasize that variations in the source cell type and culture conditions can profoundly influence EV cargo, underscoring the importance of thorough profiling.[92] Second, *in vivo* pharmacokinetic studies and dormancy assays—particularly in metastatic mouse models—must monitor EV biodistribution, uptake by tumor versus healthy tissues, dormancy activation, and recurrence over time. Techniques like fluorescent or bioluminescent labeling already allow precise tracking of EV kinetics and tissue targeting in animal models.[93] Third, standardized GMP-compliant manufacturing is essential; scalable production protocols must incorporate quality controls for source cell consistency, cargo purity, sterility, potency assays, and batch-to-batch reproducibility.[94] Early-phase clinical trials should include close monitoring for dormancy activation, drug resistance, and immune changes, particularly in post-remission breast cancer patients. This comprehensive approach—covering cargo safety, pharmacokinetics, manufacturing quality, and clinical monitoring—will be critical to translating MSC-EVs into effective and reliable breast cancer therapies.

Future studies should integrate MISEV 2023 standards, apply comprehensive cargo profiling, and incorporate pharmacokinetic and dormancy assessments *in vivo* under GMP-compliant conditions to facilitate safe clinical translation.

Conclusion

MSC-EVs show significant therapeutic potential against breast cancer when precisely engineered. However, dormancy risks necessitate strict cargo control, targeted delivery, and long-term safety validation to ensure their safe translation into clinical therapy.

MATERIAL AND METHODS

Study design

This review was conducted following the PRISMA 2020 guidelines[95] and was structured as a systematic review and meta-analysis of *in vitro* experimental studies evaluating the effects of MSC-EVs in breast cancer models. The primary objective was to synthesize evidence on the therapeutic impact of MSC-EVs on key tumor-related processes, including cell proliferation, apoptosis, and migration. The review also included quality assessment and quantitative synthesis of effect sizes where applicable. A completed PRISMA 2020 checklist is provided in Table S1. This review is prospectively registered in OSF (internet archive link: https://archive.org/details/osf-registrations-xm4ak-v1; registration DOI:10.17605/OSF.IO/XM4AK).

Search strategy

A comprehensive literature search was performed across three major databases—PubMed, Scopus, and Web of Science. The search strategy combined medical subject headings and keyword terms relevant to EVs, MSCs, and breast cancer. Boolean operators were used to expand or narrow search results appropriately. The complete list of queries used is provided in Table S2.

The search included all publications available up to July 2025. Only articles published in English were considered for inclusion. Search terms were grouped into three main categories: (1) terms describing EVs, (2) terms identifying MSCs from various tissues, and (3) terms related to breast cancer. The final search query combined all three domains using the Boolean operator AND to ensure relevance to the scope of the review.

Inclusion and exclusion criteria

Original *in vitro* research articles that evaluated the therapeutic effects of MSC-EVs on breast cancer cells were considered eligible. Studies were considered if they reported outcomes related to tumor biology such as proliferation, apoptosis, migration, invasion, or molecular pathway modulation. Additionally, experimental designs involving engineered or drug-loaded EVs were considered eligible.

Articles were excluded if they met any of the following criteria: not written in English, review articles, conference abstracts, commentaries, case reports, or lack of sufficient methodological detail. Studies involving noncancer models or unrelated cell lines were also excluded.

Although the review focused primarily on *in vitro* studies, some articles that included complementary *in vivo* components (e.g., xenograft tumor models in mice) were retained if they also contained relevant *in vitro* data for EV intervention. No restrictions were placed on the publication year, EV loading status, or MSC tissue of origin, provided the study met the core eligibility criteria.

Data extraction

A structured approach was employed to collect and organize data from all eligible *in vitro* studies examining the effects of MSC-EVs on breast cancer cell models. Two reviewers independently extracted the relevant data by using a pre-designed Excel template, ensuring consistency and reproducibility. Any discrepancies were discussed and resolved by consensus, with the involvement of a third reviewer when necessary. Extracted variables included study origin, MSC source, EV isolation and characterization techniques, type of EV loading (e.g., miRNA, chemotherapeutics), breast cancer cell lines tested, and experimental conditions such as dosage, treatment duration, and control types. Additionally, information on primary outcomes (e.g., cell proliferation, apoptosis, and migration), secondary endpoints (e.g., gene or protein expression changes), and key findings were captured. When available, details regarding the limitations and funding sources were also recorded to assess transparency and potential conflicts of interest. When multiple experiments from a single publication used identical cell lines and MSC sources, only the most representative dataset or the one with complete variance data was included to maintain statistical independence. Quantitative data were primarily obtained from tables or text in the included

papers. When only graphical results were available, numerical values were extracted using WebPlotDigitizer (v.4.6, Ankit Rohatgi) to ensure accuracy and comparability.

Quality assessment

The SYRCLE's Risk of Bias tool was adapted for *in vitro* settings because it provides structured bias domains (selection, performance, detection, and reporting) compatible with bench-based designs.[96] Alternative tools such as OHAT and ToxRTool were reviewed but lacked granularity for EV-specific experiments. To evaluate the methodological rigor and identify potential biases, a modified version of the SYRCLE's Risk of Bias tool was applied. Although originally intended for animal studies, this framework was adapted to suit *in vitro* experimental designs. Although originally developed for animal experiments, the SYRCLE tool has been successfully adapted for *in vitro* systematic reviews. Its structured bias domains make it suitable for bench-based designs. The assessment covered six key domains: selection of MSCs and cell lines, consistency of experimental conditions, objectivity and reproducibility of outcome measures, completeness of result reporting, methodological transparency, and any additional concerns such as incomplete EV characterization or undisclosed conflicts of interest. Each domain was rated as having a low, high, or unclear risk of bias based on explicit criteria. This evaluation was conducted independently by two reviewers, with disagreements resolved through discussion.

Statistical analysis

Quantitative data from studies reporting means and standard deviations were synthesized using meta-analytic techniques. SMDs were calculated for continuous outcomes such as cell proliferation rates, apoptosis rates, and migration distances. Given the anticipated heterogeneity across cell lines, MSC sources, and experimental protocols, a random-effects model was chosen. Between-study heterogeneity was quantified using the I^2 statistic, with thresholds of 25%, 50%, and 75% interpreted as denoting low, moderate, and high heterogeneity, respectively. Where feasible, subgroup analyses were conducted to explore the influence of EV loading (e.g., drug-loaded EVs versus native EVs), MSC origin (e.g., bone marrow versus adipose tissue), and breast cancer subtype. Sensitivity analyses were also performed to assess robustness of the findings by excluding studies deemed to be at high risk of bias. All statistical analyses were carried out using RevMan version 5.4 and R software (version 4.3.2), employing the "meta" and "metafor" packages for meta-analytic computations and forest plot generation. Subject number refers to the number of independent biological replicates (n) per cell line per condition, ensuring that no repeated measures were double counted. Each effect size represents an independent cell line-MSC source experiment. Standard deviations were harmonized to the same scale (mean ± SD per replicate) and converted to SMDs (Hedges' g) to control for inter-study variance.

ACKNOWLEDGMENTS

The study was supported by the program-targeted financing on scientific programs of the Ministry of Healthcare of the Republic of Kazakhstan «Development of an EV isolation kit from umbilical cord mesenchymal stem cell culture for therapeutic and research application» (2024–2026) (IRN BR25593457). This study is a systematic review and meta-analysis that synthesizes data from previously published studies. No primary data were collected from human participants by the authors. Therefore, ethical approval was not required for this research. However, all included studies were reviewed to ensure that they had obtained ethical approval from their respective institutional review boards and complied with international ethical standards.

AUTHOR CONTRIBUTIONS

Conceptualization, A.T.; methodology, A.T., N.M.M., K.R.Z., and A.B.; formal analysis, N.M.M. and A.B.; investigation, N.M.M., M.A.K., and L.A.I.; data curation, N.M.M.; writing – original draft, N.M.M., K.R.Z., and A.B.; writing – review & editing, A.T., M.A.K., and L.A.I.; visualization, A.B. and K.R.Z.; supervision, A.T.; project administration, N.M.M.; funding acquisition, N.M.M. and A.T. All authors have read and agreed to the published version of the manuscript.

DECLARATION OF INTERESTS

The authors declare no competing interests.

SUPPLEMENTAL INFORMATION

Supplemental information can be found online at https://doi.org/10.1016/j.omton.2025.201107.

REFERENCES

1. Halim, N.S.S.A., Wisasi, M., Rungsisiwut, R., Mohamed, R., Shaharuddin, B., Suwan, N., Bakar, N.F.A.A., Ghozali, M., Atik, N., Syamsunarno, M.R.A., et al. (2025). Revolutionizing Breast Cancer Treatment: The Role of Regenerative Medicine. Biomed. Res. Ther. *12*, 7489–7498. https://doi.org/10.15419/tjcsrg93.

2. Anand, U., Dey, A., Chandel, A.K.S., Sanyal, R., Mishra, A., Pandey, D.K., De Falco, V., Upadhyay, A., Kandimalla, R., Chaudhary, A., et al. (2023). Cancer chemotherapy and beyond: Current status, drug candidates, associated risks and progress in targeted therapeutics. Genes Dis. *10*, 1367–1401. https://doi.org/10.1016/j.gendis.2022.02.007.

3. Wu, X., Meng, Y., Yao, Z., Lin, X., Hu, M., Cai, S., Gao, S., and Zhang, H. (2025). Extracellular vesicles as nature's nano carriers in cancer therapy: Insights toward preclinical studies and clinical applications. Pharmacol. Res. *217*, 107751. https://doi.org/10.1016/j.phrs.2025.107751.

4. Weng, Z., Zhang, B., Wu, C., Yu, F., Han, B., Li, B., and Li, L. (2021). Therapeutic roles of mesenchymal stem cell-derived extracellular vesicles in cancer. J. Hematol. Oncol. *14*, 136. https://doi.org/10.1186/s13045-021-01141-y.

5. Goyal, A., Afzal, M., Goyal, K., Ganesan, S., Kumari, M., Sunitha, S., Dash, A., Saini, S., Rana, M., Gupta, G., et al. (2025). MSC-derived extracellular vesicles: Precision miRNA delivery for overcoming cancer therapy resistance. Regen. Ther. *29*, 303–318. https://doi.org/10.1016/j.reth.2025.03.006.

6. Wang, B., Hu, S., Teng, Y., Chen, J., Wang, H., Xu, Y., Wang, K., Xu, J., Cheng, Y., and Gao, X. (2024). Current advance of nanotechnology in diagnosis and treatment for malignant tumors. Signal Transduct. Target. Ther. *9*, 200. https://doi.org/10.1038/s41392-024-01889-y.

7. Jahangiri, B., Khalaj-Kondori, M., Asadollahi, E., Kian Saei, A., and Sadeghizadeh, M. (2023). Dual impacts of mesenchymal stem cell-derived exosomes on cancer cells: unravelling complex interactions. J. Cell Commun. Signal. *17*, 1229–1247. https://doi.org/10.1007/s12079-023-00794-3.

8. Ramos, C.C., Pires, J., Gonzalez, E., Garcia-Vallicrosa, C., Reis, C.A., Falcon-Perez, J.M., and Freitas, D. (2024). Extracellular vesicles in tumor-adipose tissue crosstalk: key drivers and therapeutic targets in cancer cachexia. Extracell. Vesicles Circ. Nucl. Acids *5*, 371–396. https://doi.org/10.20517/evcna.2024.36.

9. Ababneh, N.A., Aldiqs, R., Nashwan, S., Ismail, M.A., Barham, R., Al Hadidi, S., Alrefae, A., Alhallaq, F.K., Abu-Humaidan, A.H., Saleh, T., and Awidi, A. (2025). Distinct anticancer properties of exosomes from induced mesenchymal stem cells vs. bone marrow-derived stem cells in MCF7 and A549 models. Biomed. Rep. *23*, 116. https://doi.org/10.3892/br.2025.1994.

10. Aldiqs, R., Nashwan, S., Ismail, M.A., Saleh, T., Barham, R., Zihlif, M., and Ababneh, N.A. (2025). Effect of exosomes derived from induced and human adipose

tissue-derived mesenchymal stem cells on human cancer cells. J. Biosci. *50*, 43. https://doi.org/10.1007/s12038-025-00510-w.

11. Altanerova, U., Jakubechova, J., Benejova, K., Priscakova, P., Pesta, M., Pitule, P., Topolcan, O., Kausitz, J., Zduriencikova, M., Repiska, V., and Altaner, C. (2019). Prodrug suicide gene therapy for cancer targeted intracellular by mesenchymal stem cell exosomes. Int. J. Cancer *144*, 897–908. https://doi.org/10.1002/ijc.31792.

12. Attar, F.A., Irani, S., Oloomi, M., Bolhassani, A., Geranpayeh, L., and Atyabi, F. (2025). Doxorubicin loaded exosomes inhibit cancer-associated fibroblasts growth: in vitro and in vivo study. Cancer Cell Int. *25*, 72. https://doi.org/10.1186/s12935-025-03689-y.

13. Bliss, S.A., Sinha, G., Sandiford, O.A., Williams, L.M., Engelberth, D.J., Guiro, K., Isenalumhe, L.L., Greco, S.J., Ayer, S., Bryan, M., et al. (2016). Mesenchymal Stem Cell-Derived Exosomes Stimulate Cycling Quiescence and Early Breast Cancer Dormancy in Bone Marrow. Cancer Res. *76*, 5832–5844. https://doi.org/10.1158/0008-5472.CAN-16-1092.

14. Casson, J., Davies, O.G., Smith, C.A., Dalby, M.J., and Berry, C.C. (2018). Mesenchymal stem cell-derived extracellular vesicles may promote breast cancer cell dormancy. J. Tissue Eng. *9*, 2041731418810093. https://doi.org/10.1177/2041731418810093.

15. Chang, Y.H., Vuong, C.K., Ngo, N.H., Yamashita, T., Ye, X., Futamura, Y., Fukushige, M., Obata-Yasuoka, M., Hamada, H., Osaka, M., et al. (2022). Extracellular vesicles derived from Wharton's Jelly mesenchymal stem cells inhibit the tumor environment via the miR-125b/HIF1alpha signaling pathway. Sci. Rep. *12*, 13550. https://doi.org/10.1038/s41598-022-17767-y.

16. Chulpanova, D.S., Gilazieva, Z.E., Akhmetzyanova, E.R., Kletukhina, S.K., Rizvanov, A.A., and Solovyeva, V.V. (2021). Cytochalasin B-induced membrane vesicles from human mesenchymal stem cells overexpressing TRAIL, PTEN and IFN-beta1 can kill carcinoma cancer cells. Tissue Cell *73*, 101664. https://doi.org/10.1016/j.tice.2021.101664.

17. Chulpanova, D.S., Gilazieva, Z.E., Kletukhina, S.K., Aimaletdinov, A.M., Garanina, E.E., James, V., Rizvanov, A.A., and Solovyeva, V.V. (2021). Cytochalasin B-Induced Membrane Vesicles from Human Mesenchymal Stem Cells Overexpressing IL2 Are Able to Stimulate CD8(+) T-Killers to Kill Human Triple Negative Breast Cancer Cells. Biology *10*, 141. https://doi.org/10.3390/biology10020141.

18. Chulpanova, D.S., Pukhalskaia, T.V., Gilazieva, Z.E., Filina, Y.V., Mansurova, M.N., Rizvanov, A.A., and Solovyeva, V.V. (2023). Cytochalasin B-Induced Membrane Vesicles from TRAIL-Overexpressing Mesenchymal Stem Cells Induce Extrinsic Pathway of Apoptosis in Breast Cancer Mouse Model. Curr. Issues Mol. Biol. *45*, 571–592. https://doi.org/10.3390/cimb45010038.

19. Ding, Y.N., Ding, H.Y., Li, H., Yang, R., Huang, J.Y., Chen, H., Wang, L.H., Wang, Y.J., Hu, C.M., An, Y.L., et al. (2023). Photosensitive small extracellular vesicles regulate the immune microenvironment of triple negative breast cancer. Acta Biomater. *167*, 534–550. https://doi.org/10.1016/j.actbio.2023.06.004.

20. Du, L., Tao, X., and Shen, X. (2021). Human umbilical cord mesenchymal stem cell-derived exosomes inhibit migration and invasion of breast cancer cells via miR-21-5p/ZNF367 pathway. Breast Cancer *28*, 829–837. https://doi.org/10.1007/s12282-021-01218-z.

21. Ebrahimian, M., Hashemi, M., Etemad, L., and Salmasi, Z. (2022). Thymoquinone-loaded mesenchymal stem cell-derived exosome as an efficient nano-system against breast cancer cells. Iran. J. Basic Med. Sci. *25*, 723–731. https://doi.org/10.22038/IJBMS.2022.64092.14116.

22. Egea, V., Kessenbrock, K., Lawson, D., Bartelt, A., Weber, C., and Ries, C. (2021). Let-7f miRNA regulates SDF-1alpha- and hypoxia-promoted migration of mesenchymal stem cells and attenuates mammary tumor growth upon exosomal release. Cell Death Dis. *12*, 516. https://doi.org/10.1038/s41419-021-03789-3.

23. Eiro, N., Fraile, M., Escudero-Cernuda, S., Sendon-Lago, J., Gonzalez, L.O., Fernandez-Sánchez, M.L., and Vizoso, F.J. (2024). Synergistic effect of human uterine cervical mesenchymal stem cell secretome and paclitaxel on triple negative breast cancer. Stem Cell Res. Ther. *15*, 121. https://doi.org/10.1186/s13287-024-03717-0.

24. Farhadi, S., Mohammadi-Yeganeh, S., Kiani, J., Hashemi, S.M., Koochaki, A., Sharifi, K., and Ghanbarian, H. (2023). Exosomal delivery of 7SK long non-coding RNA suppresses viability, proliferation, aggressiveness and tumorigenicity in triple negative breast cancer cells. Life Sci. *322*, 121646. https://doi.org/10.1016/j.lfs.2023.121646.

25. Farouk, A.H., Aref, A., Fathy, B.A., and Abdallah, A.N. (2024). Stem cells derived exosomes as biological nano carriers for VCR sulfate for treating breast cancer stem cells. Sci. Rep. *14*, 10964. https://doi.org/10.1038/s41598-024-59736-7.

26. Felthaus, O., Vedlin, S., Eigenberger, A., Klein, S.M., and Prantl, L. (2024). Exosomes from Adipose-Tissue-Derived Stem Cells Induce Proapoptotic Gene Expression in Breast Tumor Cell Line. Int. J. Mol. Sci. *25*, 2190. https://doi.org/10.3390/ijms25042190.

27. Gomari, H., Forouzandeh Moghadam, M., and Soleimani, M. (2018). Targeted cancer therapy using engineered exosome as a natural drug delivery vehicle. OncoTargets Ther. *11*, 5753–5762. https://doi.org/10.2147/OTT.S173110.

28. Gomari, H., Forouzandeh Moghadam, M., Soleimani, M., Ghavami, M., and Khodashenas, S. (2019). Targeted delivery of doxorubicin to HER2 positive tumor models. Int. J. Nanomedicine *14*, 5679–5690. https://doi.org/10.2147/IJN.S210731.

29. Hass, R., von der Ohe, J., and Luo, T. (2024). Human mesenchymal stroma/stem-like cell-derived taxol-loaded EVs/exosomes transfer anti-tumor microRNA signatures and express enhanced SDF-1-mediated tumor tropism. Cell Commun. Signal. *22*, 506. https://doi.org/10.1186/s12964-024-01886-2.

30. Hosseini, M., Ezzeddini, R., Hashemi, S.M., Soudi, S., and Salek Farrokhi, A. (2024). Enhanced anti-tumor efficacy of S3I-201 in breast cancer mouse model through Wharton jelly- exosome. Cancer Cell Int. *24*, 318. https://doi.org/10.1186/s12935-024-03501-3.

31. Hu, Y., Liu, H., Xiao, X., Yu, Q., Deng, R., Hua, L., Wang, J., and Wang, X. (2022). Bone Marrow Mesenchymal Stem Cell-Derived Exosomes Inhibit Triple-Negative Breast Cancer Cell Stemness and Metastasis via an ALKBH5-Dependent Mechanism. Cancers (Basel) *14*, 6059. https://doi.org/10.3390/cancers14246059.

32. Jafarpour, S., Ahmadi, S., Mokarian, F., Sharifi, M., Ghobakhloo, S., Yazdi, M., Nedaeinia, R., and Salehi, R. (2024). MSC-derived exosomes enhance the anticancer activity of drugs in 3D spheroid of breast cancer cells. J. Drug Deliv. Sci. Technol. *92*, 105375. https://doi.org/10.1016/j.jddst.2024.105375.

33. Jia, Z., Zhu, H., Sun, H., Hua, Y., Zhang, G., Jiang, J., and Wang, X. (2020). Adipose Mesenchymal Stem Cell-Derived Exosomal microRNA-1236 Reduces Resistance of Breast Cancer Cells to Cisplatin by Suppressing SLC9A1 and the Wnt/beta-Catenin Signaling. Cancer Manag. Res. *12*, 8733–8744. https://doi.org/10.2147/CMAR.S270200.

34. Kaan, D. (2023). The Improving anti-tumour activity with melatonin-stimulated mesenchymal stem cell-derived exosomes in metastatic triple-negative breast cancer. Indian J. Biochem. Biophys. *60*, 817–827. https://doi.org/10.56042/ijbb.v60i11.1291.

35. Kalimuthu, S., Gangadaran, P., Rajendran, R.L., Zhu, L., Oh, J.M., Lee, H.W., Gopal, A., Baek, S.H., Jeong, S.Y., Lee, S.W., et al. (2018). A New Approach for Loading Anticancer Drugs Into Mesenchymal Stem Cell-Derived Exosome Mimetics for Cancer Therapy. Front. Pharmacol. *9*, 1116. https://doi.org/10.3389/fphar.2018.01116.

36. Khazaei-Poul, Y., Shojaei, S., Koochaki, A., Ghanbarian, H., and Mohammadi-Yeganeh, S. (2021). Evaluating the influence of Human Umbilical Cord Mesenchymal Stem Cells-derived exosomes loaded with miR-3182 on metastatic performance of Triple Negative Breast Cancer cells. Life Sci. *286*, 120015. https://doi.org/10.1016/j.lfs.2021.120015.

37. Khazaei-Poul, Y., Mirmotalebisohi, S.A., Zali, H., Molavi, Z., and Mohammadi-Yeganeh, S. (2023). Identification of miR-3182 and miR-3143 target genes involved in the cell cycle as a novel approach in TNBC treatment: A systems biology approach. Chem. Biol. Drug Des. *101*, 662–677. https://doi.org/10.1111/cbdd.14167.

38. Lee, J.K., Park, S.R., Jung, B.K., Jeon, Y.K., Lee, Y.S., Kim, M.K., Kim, Y.G., Jang, J.Y., and Kim, C.W. (2013). Exosomes derived from mesenchymal stem cells suppress angiogenesis by down-regulating VEGF expression in breast cancer cells. PLoS One *8*, e84256. https://doi.org/10.1371/journal.pone.0084256.

39. Liu, M., Hu, Y., and Chen, G. (2020). The Antitumor Effect of Gene-Engineered Exosomes in the Treatment of Brain Metastasis of Breast Cancer. Front. Oncol. *10*, 1453. https://doi.org/10.3389/fonc.2020.01453.

40. Liu, Q., Zhang, J., Liu, Y., Peng, H., and Wu, Y. (2022). Extracellular vesicles extracted from bone marrow mesenchymal stem cells carrying MicroRNA-342-3p inhibit the INHBA/IL13Ralpha2 axis to suppress the growth and metastasis of breast cancer. Transl. Oncol. *18*, 101333. https://doi.org/10.1016/j.tranon.2021.101333.

41. Melzer, C., Rehn, V., Yang, Y., Bähre, H., von der Ohe, J., and Hass, R. (2019). Taxol-Loaded MSC-Derived Exosomes Provide a Therapeutic Vehicle to Target Metastatic Breast Cancer and Other Carcinoma Cells. Cancers (Basel) *11*, 798. https://doi.org/10.3390/cancers11060798.

42. Melzer, C., Ohe, J.v.d., and Hass, R. (2020). Anti-Tumor Effects of Exosomes Derived from Drug-Incubated Permanently Growing Human MSC. Int. J. Mol. Sci. *21*, 7311. https://doi.org/10.3390/ijms21197311.

43. Meng, W., Wang, L., Du, X., Xie, M., Yang, F., Li, F., Wu, Z.E., Gan, J., Wei, H., Cao, C., et al. (2023). Engineered mesenchymal stem cell-derived extracellular vesicles constitute a versatile platform for targeted drug delivery. J. Control. Release *363*, 235–252. https://doi.org/10.1016/j.jconrel.2023.09.037.

44. Mirabdollahi, M., Sadeghi-Aliabadi, H., and Haghjooy Javanmard, S. (2020). Human Wharton's jelly mesenchymal stem cells-derived secretome could inhibit breast cancer growth in vitro and in vivo. Iran. J. Basic Med. Sci. *23*, 945–953. https://doi.org/10.22038/ijbms.2020.42477.10020.

45. Mohd Ali, N., Yeap, S.K., Ho, W.Y., Boo, L., Ky, H., Satharasinghe, D.A., Tan, S.W., Cheong, S.K., Huang, H.D., Lan, K.C., et al. (2020). Adipose MSCs Suppress MCF7 and MDA-MB-231 Breast Cancer Metastasis and EMT Pathways Leading to Dormancy via Exosomal-miRNAs Following Co-Culture Interaction. Pharmaceuticals *14*, 8–29. https://doi.org/10.3390/ph14010008.

46. Naseri, Z., Oskuee, R.K., Jaafari, M.R., and Forouzandeh Moghadam, M. (2018). Exosome-mediated delivery of functionally active miRNA-142-3p inhibitor reduces tumorigenicity of breast cancer in vitro and in vivo. Int. J. Nanomedicine *13*, 7727–7747. https://doi.org/10.2147/IJN.S182384.

47. Naseri, Z., Oskuee, R.K., Forouzandeh-Moghadam, M., and Jaafari, M.R. (2020). Delivery of LNA-antimiR-142-3p by Mesenchymal Stem Cells-Derived Exosomes to Breast Cancer Stem Cells Reduces Tumorigenicity. Stem Cell Rev. Rep. *16*, 541–556. https://doi.org/10.1007/s12015-019-09944-w.

48. O'Brien, K.P., Khan, S., Gilligan, K.E., Zafar, H., Lalor, P., Glynn, C., O'Flatharta, C., Ingoldsby, H., Dockery, P., De Bhulbh, A., et al. (2018). Employing mesenchymal stem cells to support tumor-targeted delivery of extracellular vesicle (EV)-encapsulated microRNA-379. Oncogene *37*, 2137–2149. https://doi.org/10.1038/s41388-017-0116-9.

49. Pakravan, K., Babashah, S., Sadeghizadeh, M., Mowla, S.J., Mossahebi-Mohammadi, M., Ataei, F., Dana, N., and Javan, M. (2017). MicroRNA-100 shuttled by mesenchymal stem cell-derived exosomes suppresses in vitro angiogenesis through modulating the mTOR/HIF-1alpha/VEGF signaling axis in breast cancer cells. Cell. Oncol. *40*, 457–470. https://doi.org/10.1007/s13402-017-0335-7.

50. Patel, N., Kommineni, N., Surapaneni, S.K., Kalvala, A., Yaun, X., Gebeyehu, A., Arthur, P., Duke, L.C., York, S.B., Bagde, A., et al. (2021). Cannabidiol loaded extracellular vesicles sensitize triple-negative breast cancer to doxorubicin in both in-vitro and in vivo models. Int. J. Pharm. *607*, 120943. https://doi.org/10.1016/j.ijpharm.2021.120943.

51. Ramirez, J.A., Jiménez, M.C., Ospina, V., Rivera, B.S., Fiorentino, S., Barreto, A., and Restrepo, L.M. (2024). The secretome from human-derived mesenchymal stem cells augments the activity of antitumor plant extracts in vitro. Histochem. Cell Biol. *161*, 409–421. https://doi.org/10.1007/s00418-024-02265-1.

52. Rezaie, Z., Ardeshirylajimi, A., and Ashkezari, M.D. (2018). Improved anticancer properties of stem cells derived exosomes by prolonged release from PCL nanofibrous structure. Gene *665*, 105–110. https://doi.org/10.1016/j.gene.2018.05.003.

53. Sandiford, O.A., Donnelly, R.J., El-Far, M.H., Burgmeyer, L.M., Sinha, G., Pamarthi, S.H., Sherman, L.S., Ferrer, A.I., DeVore, D.E., Patel, S.A., et al. (2021). Mesenchymal Stem Cell-Secreted Extracellular Vesicles Instruct Stepwise Dedifferentiation of Breast Cancer Cells into Dormancy at the Bone Marrow Perivascular Region. Cancer Res. *81*, 1567–1582. https://doi.org/10.1158/0008-5472.CAN-20-2434.

54. Sheykhhasan, M., Kalhor, N., Sheikholeslami, A., Dolati, M., Amini, E., and Fazaeli, H. (2021). Exosomes of Mesenchymal Stem Cells as a Proper Vehicle for Transfecting miR-145 into the Breast Cancer Cell Line and Its Effect on Metastasis. BioMed Res. Int. *2021*, 5516078. https://doi.org/10.1155/2021/5516078.

55. Shojaei, S., Hashemi, S.M., Ghanbarian, H., Sharifi, K., Salehi, M., and Mohammadi-Yeganeh, S. (2021). Delivery of miR-381-3p Mimic by Mesenchymal Stem Cell-Derived Exosomes Inhibits Triple Negative Breast Cancer Aggressiveness; an In Vitro Study. Stem Cell Rev. Rep. *17*, 1027–1038. https://doi.org/10.1007/s12015-020-10089-4.

56. Shojaei, S., Moradi-Chaleshtori, M., Paryan, M., Koochaki, A., Sharifi, K., and Mohammadi-Yeganeh, S. (2023). Mesenchymal stem cell-derived exosomes enriched with miR-218 reduce the epithelial-mesenchymal transition and angiogenesis in triple-negative breast cancer cells. Eur. J. Med. Res. *28*, 516. https://doi.org/10.1186/s40001-023-01463-2.

57. Sun, H., Dai, J., Chen, M., Chen, Q., Xie, Q., Zhang, W., Li, G., and Yan, M. (2022). miR-139-5p Was Identified as Biomarker of Different Molecular Subtypes of Breast Carcinoma. Front. Oncol. *12*, 857714. https://doi.org/10.3389/fonc.2022.857714.

58. Ulpiano, C., Salvador, W., Franchi-Mendes, T., Huang, M.C., Lin, Y.H., Lin, H.T., Rodrigues, C.A.V., Fernandes-Platzgummer, A., Cabral, J.M.S., Monteiro, G.A., and da Silva, C.L. (2025). Continuous collection of human mesenchymal-stromal-cell-derived extracellular vesicles from a stirred tank reactor operated under xenogeneic-free conditions for therapeutic applications. Stem Cell Res. Ther. *16*, 210. https://doi.org/10.1186/s13287-025-04341-2.

59. Vakhshiteh, F., Rahmani, S., Ostad, S.N., Madjd, Z., Dinarvand, R., and Atyabi, F. (2021). Exosomes derived from miR-34a-overexpressing mesenchymal stem cells inhibit in vitro tumor growth: A new approach for drug delivery. Life Sci. *266*, 118871. https://doi.org/10.1016/j.lfs.2020.118871.

60. Wang, Y., Wang, P., Zhao, L., Chen, X., Lin, Z., Zhang, L., and Li, Z. (2021). miR-224-5p Carried by Human Umbilical Cord Mesenchymal Stem Cells-Derived Exosomes Regulates Autophagy in Breast Cancer Cells via HOXA5. Front. Cell Dev. Biol. *9*, 679185. https://doi.org/10.3389/fcell.2021.679185.

61. Xu, W., Wang, K., Wang, K., Zhao, Y., Yang, Z., and Li, X. (2024). Key Magnetized Exosomes for Effective Targeted Delivery of Doxorubicin Against Breast Cancer Cell Types in Mice Model. Int. J. Nanomedicine *19*, 10711–10724. https://doi.org/10.2147/IJN.S479306.

62. Yang, Z., Xu, B., Wu, S., Yang, W., Luo, R., Geng, S., Xin, Z., Jin, W., Shen, X., Gu, X., et al. (2022). Exosomal microRNA-551b-3p from bone marrow-derived mesenchymal stromal cells inhibits breast cancer progression via regulating TRIM31/Akt signaling. Hum. Cell *35*, 1797–1812. https://doi.org/10.1007/s13577-022-00753-x.

63. Zhang, Y., Lai, X., Yue, Q., Cao, F., Zhang, Y., Sun, Y., Tian, J., Lu, Y., He, L., Bai, J., and Wei, Y. (2022). Bone marrow mesenchymal stem cells-derived exosomal microRNA-16-5p restrains epithelial-mesenchymal transition in breast cancer cells via EPHA1/NF-kappaB signaling axis. Genomics *114*, 110341. https://doi.org/10.1016/j.ygeno.2022.110341.

64. Zhang, Z., Luo, X., Xue, X., Pang, M., Wang, X., Yu, L., Qian, J., Li, X., Tian, M., Lu, A., et al. (2024). Engineered Exosomes Carrying miR-588 for Treatment of Triple Negative Breast Cancer Through Remodeling the Immunosuppressive Microenvironment. Int. J. Nanomedicine *19*, 743–758. https://doi.org/10.2147/IJN.S440619.

65. Zhou, Y., Yamamoto, Y., Takeshita, F., Yamamoto, T., Xiao, Z., and Ochiya, T. (2021). Delivery of miR-424-5p via Extracellular Vesicles Promotes the Apoptosis of MDA-MB-231 TNBC Cells in the Tumor Microenvironment. Int. J. Mol. Sci. *22*, 844. https://doi.org/10.3390/ijms22020844.

66. Zhou, M., Li, H., Zhao, J., Zhang, Q., Han, Z., Han, Z.C., Zhu, L., Wang, H., and Li, Z. (2024). Extracellular vesicles derived from mesenchymal stem cells suppress breast cancer progression by inhibiting angiogenesis. Mol. Med. Rep. *30*, 192. https://doi.org/10.3892/mmr.2024.13316.

67. Almouh, M., Pakravan, K., Ghazimoradi, M.H., Motamed, R., Bakhshinejad, B., Hassan, Z.M., and Babashah, S. (2024). Exosomes released by oxidative stress-induced mesenchymal stem cells promote murine mammary tumor progression through activating the STAT3 signaling pathway. Mol. Cell. Biochem. *479*, 3375–3391. https://doi.org/10.1007/s11010-024-04934-0.

68. Chen, R., Liu, X., and Tan, N. (2023). Bone Marrow Mesenchymal Stem Cell (BMSC)-Derived Exosomes Regulates Growth of Breast Cancer Cells Mediated by Hedgehog Signaling Pathway. j. biomater. tissue eng. *13*, 157–161. https://doi.org/10.1166/jbt.2023.3224.

69. Lin, R., Wang, S., and Zhao, R.C. (2013). Exosomes from human adipose-derived mesenchymal stem cells promote migration through Wnt signaling pathway in a breast cancer cell model. Mol. Cell. Biochem. *383*, 13–20. https://doi.org/10.1007/s11010-013-1746-z.

70. Liu, X., Jiang, F., Wang, Z., Tang, L., Zou, B., Xu, P., and Yu, T. (2021). Hypoxic bone marrow mesenchymal cell-extracellular vesicles containing miR-328-3p promote lung cancer progression via the NF2-mediated Hippo axis. J. Cell Mol. Med. *25*, 96–109. https://doi.org/10.1111/jcmm.15865.

71. Movahed, Z.G., Mansouri, K., Mohsen, A.H., and Matin, M.M. (2025). Bone marrow mesenchymal stem cells enrich breast cancer stem cell population via targeting metabolic pathways. Med. Oncol. *42*, 90. https://doi.org/10.1007/s12032-025-02632-5.

72. Orso, F., Virga, F., Dettori, D., Dalmasso, A., Paradzik, M., Savino, A., Pomatto, M.A.C., Quirico, L., Cucinelli, S., Coco, M., et al. (2023). Stroma-derived miR-214 coordinates tumor dissemination. J. Exp. Clin. Cancer Res. *42*, 20. https://doi.org/10.1186/s13046-022-02553-5.

73. Vallabhaneni, K.C., Penfornis, P., Dhule, S., Guillonneau, F., Adams, K.V., Mo, Y.Y., Xu, R., Liu, Y., Watabe, K., Vemuri, M.C., and Pochampally, R. (2015). Extracellular vesicles from bone marrow mesenchymal stem/stromal cells transport tumor regulatory microRNA, proteins, and metabolites. Oncotarget *6*, 4953–4967. https://doi.org/10.18632/oncotarget.3211.

74. Vallabhaneni, K.C., Penfornis, P., Xing, F., Hassler, Y., Adams, K.V., Mo, Y.Y., Watabe, K., and Pochampally, R. (2017). Stromal cell extracellular vesicular cargo mediated regulation of breast cancer cell metastasis via ubiquitin conjugating enzyme E2 N pathway. Oncotarget *8*, 109861–109876. https://doi.org/10.18632/oncotarget.22371.

75. Wang, M., Zheng, Y., Hao, Q., Mao, G., Dai, Z., Zhai, Z., Lin, S., Liang, B., Kang, H., and Ma, X. (2025). Hypoxic BMSC-derived exosomal miR-210-3p promotes progression of triple-negative breast cancer cells via NFIX-Wnt/beta-catenin signaling axis. J. Transl. Med. *23*, 39. https://doi.org/10.1186/s12967-024-05947-5.

76. Wang, S., Su, X., Xu, M., Xiao, X., Li, X., Li, H., Keating, A., and Zhao, R.C. (2019). Exosomes secreted by mesenchymal stromal/stem cell-derived adipocytes promote breast cancer cell growth via activation of Hippo signaling pathway. Stem Cell Res. Ther. *10*, 117. https://doi.org/10.1186/s13287-019-1220-2.

77. Worner, P.M., Schachtele, D.J., Barabadi, Z., Srivastav, S., Chandrasekar, B., Izadpanah, R., and Alt, E.U. (2019). Breast Tumor Microenvironment Can Transform Naive Mesenchymal Stem Cells into Tumor-Forming Cells in Nude Mice. Stem Cells Dev *28*, 341–352. https://doi.org/10.1089/scd.2018.0110.

78. Wu, W., Huang, R., Ou, L., and Lei, R. (2022). Exosomes derived from bone marrow mesenchymal stem cells promote proliferation and migration via upregulation yes-associated protein/transcriptional coactivator with PDZ binding motif expression in breast cancer cells. Chin. J. Physiol. *65*, 233–240. https://doi.org/10.4103/0304-4920.359800.

79. Wu, X., Gong, L., Xiao, P., and Wan, M. (2022). Bone Marrow Mesenchymal Stem Cells (BMSCs)-Derived miR-206 Promotes Breast Cancer Development by Activating Hedgehog Gene Signaling. J. biomater. tissue eng. *12*, 1602–1608. https://doi.org/10.1166/jbt.2022.3097.

80. Xing, L., Tang, X., Wu, K., Huang, X., Yi, Y., and Huan, J. (2020). LncRNA HAND2-AS1 suppressed the growth of triple negative breast cancer via reducing secretion of MSCs derived exosomal miR-106a-5p. Aging *13*, 424–436. https://doi.org/10.18632/aging.202148.

81. Yang, Y., Bucan, V., Baehre, H., von der Ohe, J., Otte, A., and Hass, R. (2015). Acquisition of new tumor cell properties by MSC-derived exosomes. Int. J. Oncol. *47*, 244–252. https://doi.org/10.3892/ijo.2015.3001.

82. Zakiyah, N., Wanandi, S.I., Antarianto, R.D., Syahrani, R.A., and Arumsari, S. (2023). Mesenchymal Stem Cell-Derived Extracellular Vesicles Increase Human MCF7 Breast Cancer Cell Proliferation associated with OCT4 Expression and ALDH Activity. Asian Pac. J. Cancer Prev. *24*, 2781–2789. https://doi.org/10.31557/APJCP.2023.24.8.2781.

83. Zhou, X., Li, T., Chen, Y., Zhang, N., Wang, P., Liang, Y., Long, M., Liu, H., Mao, J., Liu, Q., et al. (2019). Mesenchymal stem cell-derived extracellular vesicles promote the in vitro proliferation and migration of breast cancer cells through the activation of the ERK pathway. Int. J. Oncol. *54*, 1843–1852. https://doi.org/10.3892/ijo.2019.4747.

84. Zhu, Q., Zhang, K., Cao, Y., and Hu, Y. (2024). Adipose stem cell exosomes, stimulated by pro-inflammatory factors, enhance immune evasion in triple-negative breast cancer by modulating the HDAC6/STAT3/PD-L1 pathway through the transporter UCHL1. Cancer Cell Int. *24*, 385. https://doi.org/10.1186/s12935-024-03557-1.

85. Ono, M., Kosaka, N., Tominaga, N., Yoshioka, Y., Takeshita, F., Takahashi, R.U., Yoshida, M., Tsuda, H., Tamura, K., and Ochiya, T. (2014). Exosomes from bone marrow mesenchymal stem cells contain a microRNA that promotes dormancy in metastatic breast cancer cells. Sci. Signal. *7*, ra63. https://doi.org/10.1126/scisignal.2005231.

86. Tufail, M., Jiang, C.H., and Li, N. (2025). Tumor dormancy and relapse: understanding the molecular mechanisms of cancer recurrence. Mil. Med. Res. *12*, 7. https://doi.org/10.1186/s40779-025-00595-2.

87. Wang, L., Wang, B., Wen, H., Mao, J., Ren, Y., and Yang, H. (2020). Exosomes: A rising star in breast cancer. Oncol. Rep. *44*, 407–423. https://doi.org/10.3892/or.2020.7638.

88. Yang, E., Jing, S., Wang, Y., Wang, H., Rodriguez, R., and Wang, Z. (2023). The Role of Mesenchymal Stem Cells and Exosomes in Tumor Development and Targeted Antitumor Therapies. Stem Cells Int. *2023*, 7059289. https://doi.org/10.1155/2023/7059289.

89. Wang, L., Yu, X., Zhou, J., and Su, C. (2023). Extracellular Vesicles for Drug Delivery in Cancer Treatment. Biol. Proced. Online *25*, 28. https://doi.org/10.1186/s12575-023-00220-3.

90. Murphy, D.E., de Jong, O.G., Brouwer, M., Wood, M.J., Lavieu, G., Schiffelers, R.M., and Vader, P. (2019). Extracellular vesicle-based therapeutics: natural versus engineered targeting and trafficking. Exp. Mol. Med. *51*, 1–12. https://doi.org/10.1038/s12276-019-0223-5.

91. Shan, C., Liang, Y., Wang, K., and Li, P. (2024). Mesenchymal Stem Cell-Derived Extracellular Vesicles in Cancer Therapy Resistance: from Biology to Clinical Opportunity. Int. J. Biol. Sci. *20*, 347–366. https://doi.org/10.7150/ijbs.88500.

92. McLaughlin, C., Datta, P., Singh, Y.P., Lo, A., Horchler, S., Elcheva, I.A., Ozbolat, I.T., Ravnic, D.J., and Koduru, S.V. (2022). Mesenchymal Stem Cell-Derived Extracellular Vesicles for Therapeutic Use and in Bioengineering Applications. Cells *11*, 3366. https://doi.org/10.3390/cells11213366.

93. Gangadaran, P., Hong, C.M., and Ahn, B.C. (2018). An Update on in Vivo Imaging of Extracellular Vesicles as Drug Delivery Vehicles. Front. Pharmacol. *9*, 169. https://doi.org/10.3389/fphar.2018.00169.

94. Lui, P.P.Y., and Leung, Y.T. (2022). Practical Considerations for Translating Mesenchymal Stromal Cell-Derived Extracellular Vesicles from Bench to Bed. Pharmaceutics *14*, 1684. https://doi.org/10.3390/pharmaceutics14081684.

95. Page, M.J., McKenzie, J.E., Bossuyt, P.M., Boutron, I., Hoffmann, T.C., Mulrow, C.D., Shamseer, L., Tetzlaff, J.M., Akl, E.A., Brennan, S.E., et al. (2021). The PRISMA 2020 statement: an updated guideline for reporting systematic reviews. BMJ *372*, n71. https://doi.org/10.1136/bmj.n71.

96. Hooijmans, C.R., Rovers, M.M., de Vries, R.B.M., Leenaars, M., Ritskes-Hoitinga, M., and Langendam, M.W. (2014). SYRCLE's risk of bias tool for animal studies. BMC Med. Res. Methodol. *14*, 43. https://doi.org/10.1186/1471-2288-14-43.

OMTON, Volume *34*

Supplemental information

Mesenchymal stem cell-derived extracellular vesicle therapy in breast cancer: A systematic review and meta-analysis of *in vitro* studies

Nadiar M. Mussin, Kulyash R. Zhilisbayeva, Akmaral Baspakova, Lunara A. Ishimova, Madina A. Kurmanalina, and Amin Tamadon

Table S1. PRISMA 2020 checklist

Section and Topic	Item #	Checklist item	Location in Manuscript
Title	1	Identify the report as a systematic review and/or meta-analysis.	Title page
Abstract	2	Provide a structured summary including background, objectives, data sources, eligibility criteria, participants/interventions, study appraisal, synthesis methods, results, limitations, conclusions, and registration information.	Abstract
Introduction	3	Describe the rationale for the review in the context of existing knowledge.	Introduction, paras 1–2
	4	Provide an explicit statement of the objectives or questions addressed.	Introduction, final paragraph
Methods	5	Specify inclusion and exclusion criteria and how studies were grouped for syntheses.	Materials and Methods → Inclusion/Exclusion Criteria
	6	Specify all information sources (databases, registers, websites, organizations) searched and the date last searched.	Methods → Search Strategy
	7	Present full search strategies for all databases, including any filters and limits used.	Supplemental Table S1
	8	Specify the process for selecting studies (screening, eligibility, inclusion).	Methods → Study Selection / PRISMA Flow Diagram (Fig. 1)
	9	Describe the methods of data collection/extraction from included studies.	Methods → Data Extraction
	10	List and define all variables sought (e.g., outcomes, exposures, predictors).	Methods → Data Extraction Template
	11	Specify methods used to assess risk of bias in included studies.	Methods → Quality Assessment
	12	Specify effect measures used for each outcome (e.g., risk ratio, SMD).	Methods → Statistical Analysis
	13	Describe methods of synthesis and rationale for the choice(s). State how effect estimates were combined and how heterogeneity was assessed.	Methods → Statistical Analysis
	14	Describe any methods used to assess potential reporting bias (e.g., funnel plot, Egger's test).	Results → Meta-analysis / Publication Bias (Fig. S7)
	15	Describe any methods used to assess certainty (confidence) in the body of evidence.	Discussion → Limitations
Results	16	Describe results of search and selection process, ideally using a flow diagram.	Results → Study Selection / Fig. 1

	17	Present characteristics for each study (e.g., study size, MSC source, EV isolation method).	Results → Study Characteristics / Table 1
	18	Present risk-of-bias assessments for each study.	Results → Quality Assessment / Fig. 2
	19	Present results of individual studies and syntheses, including summary statistics and forest plots.	Results → Meta-analysis / Figs 3–5
	20	Describe results of sensitivity analyses, subgroup analyses, and heterogeneity.	Results → Meta-analysis Results / Subgroup and Sensitivity Analyses
	21	Present results of any assessment of publication bias.	Results → Meta-analysis / Fig. S7
Discussion	22	Provide a general interpretation of the results in the context of other evidence.	Discussion → Dualistic Effects and Applied Engineering
	23	Discuss limitations of the evidence and of the review process.	Discussion → Limitations
	24	Discuss implications of the results for practice, policy, and future research.	Discussion → Future Directions
Other information	25	Provide registration information for the review (e.g., PROSPERO ID) or state if not registered.	Not registered – stated in Methods
	26	Indicate sources of financial or non-financial support for the review.	Acknowledgments
	27	Declare any competing interests of the review authors.	Declaration of Interest Statement
	28	Describe availability of data, code, and other materials used in the review.	Data Availability / Supplemental Information

Table S2. Detailed Boolean search strategy for three conceptual domains

Code	Query
#1 (Extracellular Vesicles)	(“Extracellular Vesicles” OR “Extracellular Vesicle” OR “Vesicle, Extracellular” OR “Vesicles, Extracellular” OR “Exovesicles” OR “Exovesicle” OR “Apoptotic Bodies” OR “Apoptotic Body” OR “Bodies, Apoptotic” OR “Body, Apoptotic” OR “Exosomes”)
#2 (Mesenchymal Stem/Stromal Cells)	(“Mesenchymal Stem Cells” OR “Mesenchymal Stem Cell” OR “Stem Cells, Mesenchymal” OR “Stem Cell, Mesenchymal” OR “Mesenchymal Stromal Cells” OR “Mesenchymal Stromal Cell” OR “Stromal Cells, Mesenchymal” OR “Stromal Cell, Mesenchymal” OR “Wharton’s Jelly Cells” OR “Wharton Jelly Cells” OR “Bone Marrow Stromal Cells” OR “Bone Marrow Stromal Cell” OR “Multipotent Bone Marrow Stromal Cells” OR “Mesenchymal Progenitor Cells” OR “Bone Marrow Mesenchymal Stem Cells” OR “Adipose-Derived Mesenchymal Stem Cells” OR “Adipose-Derived Mesenchymal Stromal Cells” OR “Adipose Tissue-Derived Mesenchymal Stem Cells” OR “Adipose Tissue-Derived Mesenchymal Stromal Cells”)
#3 (Breast Cancer/Neoplasms)	(“Breast Neoplasms” OR “Breast Neoplasm” OR “Breast Tumor” OR “Breast Tumors” OR “Breast Cancer” OR “Cancer of the Breast” OR “Breast Carcinoma” OR “Breast Carcinomas” OR “Mammary Cancer” OR “Mammary Cancers” OR “Human Mammary Neoplasms” OR “Human Mammary Carcinoma” OR “Carcinoma, Breast” OR “Carcinomas, Human Mammary”)
#4 (Combined Search)	#1 AND #2 AND #3

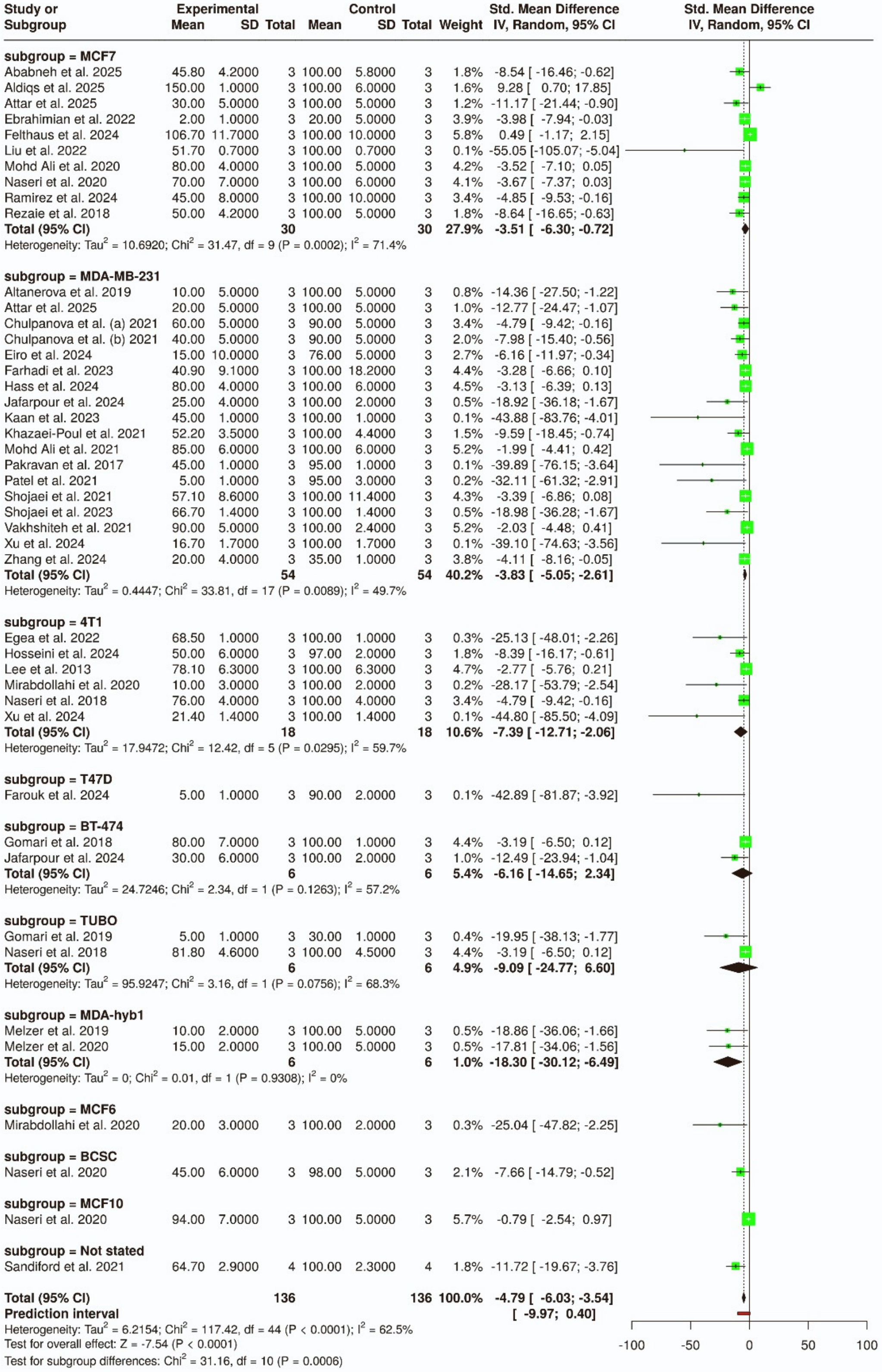

Figure S1. Forest plot of the effect of MSC-derived EVs on breast cancer cell viability, stratified by breast cancer cell line.

Study or Subgroup	Experimental Mean	Experimental SD	Experimental Total	Control Mean	Control SD	Control Total	Weight	Std. Mean Difference IV, Random, 95% CI
**subgroup = MCF6**								
Ababneh et al. 2025	10.00	1.0000	3	10.00	1.0000	3	5.7%	0.00 [-1.60; 1.60]
**subgroup = MCF7**								
Aldiqs et al. 2025	40.00	2.5000	3	30.00	2.5000	3	4.6%	3.19 [-0.12; 6.50]
Attar et al. 2025	90.00	5.0000	3	10.00	2.0000	3	0.7%	16.76 [1.46; 32.07]
Jia et al. 2020	38.00	3.0000	3	22.00	4.0000	3	4.4%	3.61 [-0.04; 7.26]
Liu et al. 2022	25.00	3.0000	3	5.00	1.2000	3	2.6%	6.98 [0.44; 13.52]
Naseri et al. 2020	3.00	1.5000	3	2.00	1.5000	3	5.7%	0.53 [-1.14; 2.20]
Wang et al. 2021	18.00	1.0000	3	6.00	1.0000	3	1.8%	9.57 [0.74; 18.41]
Yang et al. 2022	20.00	1.5000	3	3.00	0.5000	3	1.3%	12.13 [1.00; 23.26]
**Total (95% CI)**			**21**			**21**	**21.0%**	**4.61 [1.53; 7.69]**
Heterogeneity: $Tau^2 = 8.4794$; $Chi^2 = 15.99$, df = 6 (P = 0.0138); $I^2 = 62.5\%$								
**subgroup = MDA-MB-231**								
Altanerova et al. 2019	50.00	8.0000	3	10.00	3.0000	3	3.4%	5.28 [0.23; 10.34]
Attar et al. 2025	85.00	5.0000	3	5.00	1.0000	3	0.7%	17.70 [1.55; 33.86]
Chulpanova et al. 2023	60.00	5.0000	3	20.00	5.0000	3	2.9%	6.38 [0.37; 12.40]
Chulpanova et al. NA	50.00	5.0000	4	20.00	5.0000	4	4.3%	5.21 [1.46; 8.96]
Chulpanova et al. NA	20.00	5.0000	4	10.00	5.0000	4	5.6%	1.74 [-0.07; 3.55]
Eiro et al. 2024	33.00	3.0000	3	2.00	1.0000	3	1.5%	11.06 [0.89; 21.23]
Farhadi et al. 2023	18.00	7.0000	3	5.00	2.0000	3	5.2%	2.01 [-0.42; 4.45]
Jafarpour et al. 2024	78.00	4.0000	3	10.00	2.0000	3	0.7%	17.16 [1.50; 32.82]
Kaan et al. 2023	4.50	0.0000	3	1.00	0.0000	3	0.0%	
Khazaei-Poul et al. 2021	18.00	0.8000	3	13.00	0.8000	3	3.6%	4.99 [0.18; 9.79]
Sandiford et al. 2021	4.00	1.0000	4	13.00	2.0000	4	4.4%	-4.94 [-8.53; -1.36]
Shojaei et al. 2021	16.00	0.8000	3	13.00	0.8000	3	4.7%	2.99 [-0.16; 6.15]
Shojaei et al. 2023	15.00	1.5000	3	9.00	1.0000	3	4.3%	3.76 [-0.01; 7.52]
Vakhshiteh et al. 2021	24.00	3.0000	3	1.00	0.5000	3	2.1%	8.53 [0.62; 16.44]
Wang et al. 2021	18.00	1.0000	3	6.00	1.0000	3	1.8%	9.57 [0.74; 18.41]
Yang et al. 2022	25.00	2.0000	3	3.00	0.5000	3	1.3%	12.04 [0.99; 23.09]
Zhang et al. 2022	8.00	0.6000	3	3.80	0.4000	3	2.8%	6.57 [0.39; 12.75]
**Total (95% CI)**			**54**			**54**	**49.1%**	**4.57 [2.38; 6.76]**
Heterogeneity: $Tau^2 = 10.9704$; $Chi^2 = 42.01$, df = 15 (P = 0.0002); $I^2 = 64.3\%$								
**subgroup = TNBC**								
Ding et al. 2023	40.00	5.0000	3	5.00	2.0000	3	2.5%	7.33 [0.49; 14.18]
**subgroup = 4T1**								
Hosseini et al. 2024	67.00	3.0000	3	4.00	1.0000	3	0.4%	22.48 [2.01; 42.95]
Naseri et al. 2018	6.00	0.1000	3	0.00	0.0000	3	0.0%	
**subgroup = BT-474**								
Jafarpour et al. 2024	82.00	4.0000	3	20.00	3.0000	3	1.0%	13.99 [1.19; 26.80]
**subgroup = MDA-MB-232**								
Jia et al. 2020	36.00	4.0000	3	18.00	4.0000	3	4.4%	3.59 [-0.04; 7.22]
**subgroup = MDA-MB-468**								
Meng et al. 2023	40.00	4.0000	3	1.00	0.5000	3	1.5%	10.92 [0.88; 20.96]
**subgroup = TUBO**								
Naseri et al. 2018	6.00	0.1000	3	0.00	0.0000	3	0.0%	
**subgroup = BCSC**								
Naseri et al. 2020	7.00	1.0000	3	1.00	1.0000	3	3.7%	4.79 [0.16; 9.42]
**subgroup = MCF10**								
Naseri et al. 2020	1.00	1.0000	3	1.00	1.0000	3	5.7%	0.00 [-1.60; 1.60]
**subgroup = SK-BR-3**								
Zhang et al. 2022	6.30	0.4000	3	5.00	0.4000	3	4.9%	2.59 [-0.25; 5.44]
**Total (95% CI)**			**108**			**108**	**100.0%**	**4.16 [2.75; 5.56]**
**Prediction interval**								**[-1.91; 10.23]**

Heterogeneity: $Tau^2 = 8.3396$; $Chi^2 = 87.55$, df = 31 (P < 0.0001); $I^2 = 64.6\%$
Test for overall effect: Z = 5.80 (P < 0.0001)
Test for subgroup differences: $Chi^2 = 34.90$, df = 10 (P = 0.0001)

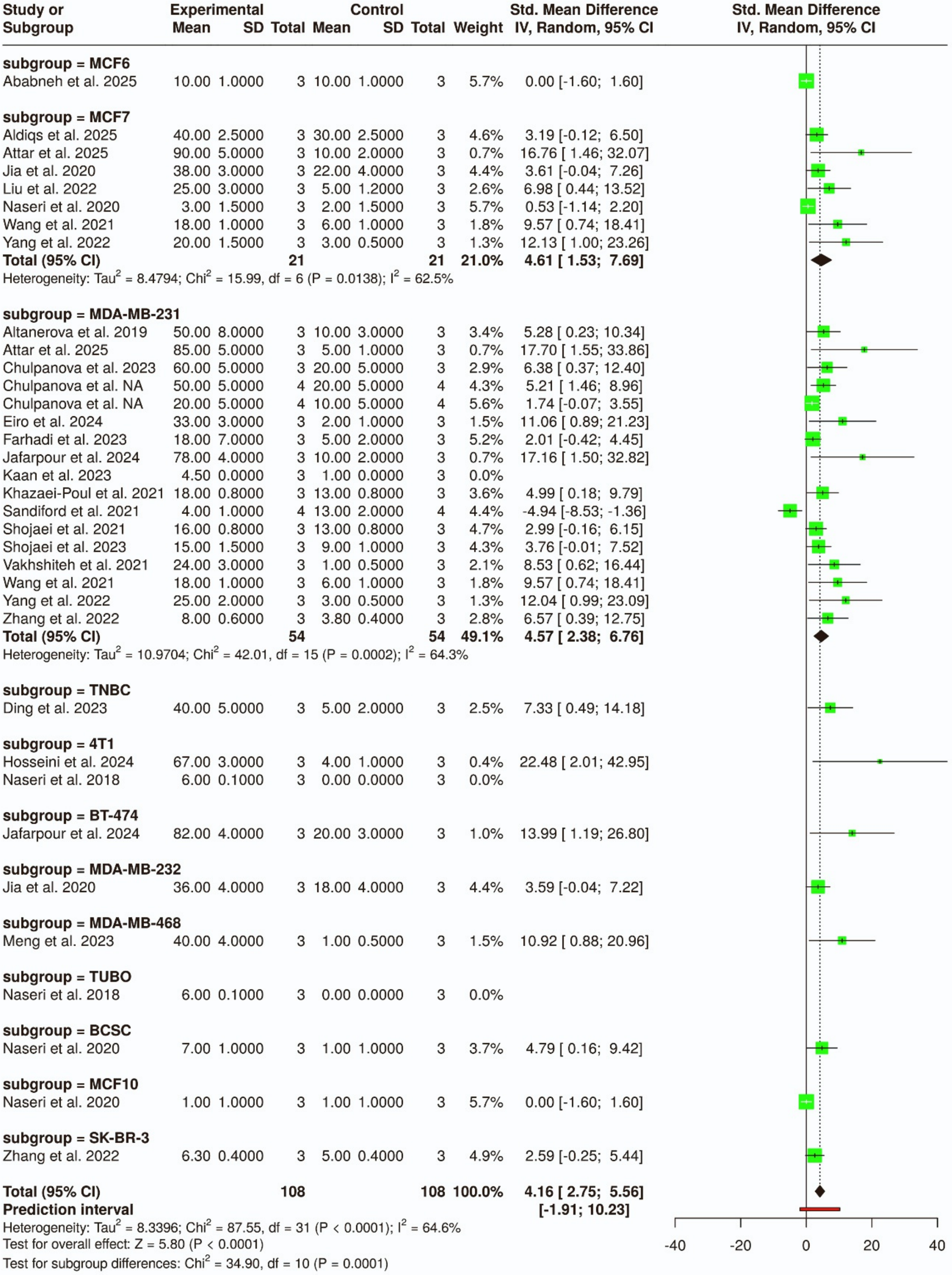

Figure S2. Forest plot of standardized mean differences in breast cancer cell apoptosis following MSC-EV treatment, stratified by breast cancer cell line.

Study or Subgroup	Experimental Mean	Experimental SD	Experimental Total	Control Mean	Control SD	Control Total	Weight	Std. Mean Difference IV, Random, 95% CI
**subgroup = MCF7**								
Ababneh et al. 2025	80.00	10.0000	3	100.00	10.0000	3	7.5%	-1.60 [-3.75; 0.56]
Aldiqs et al. 2025	125.00	13.0000	3	100.00	13.0000	3	7.5%	1.53 [-0.59; 3.66]
Du et al. 2021	40.00	5.0000	3	80.00	5.0000	3	4.4%	-6.38 [-12.40; -0.37]
Liu et al. 2022	20.00	2.0000	3	60.00	5.0000	3	3.4%	-8.38 [-16.16; -0.61]
Mohd Ali et al. 2020	40.00	4.0000	3	80.00	5.0000	3	4.0%	-7.05 [-13.65; -0.45]
Ramirez et al. 2024	5.00	1.0000	3	30.00	1.0000	3	0.9%	-19.95 [-38.13; -1.77]
Yang et al. 2022	48.00	3.0000	3	75.00	4.0000	3	4.6%	-6.09 [-11.85; -0.33]
**Total (95% CI)**			**21**			**21**	**32.4%**	**-4.32 [-7.88; -0.76]**
Heterogeneity: $Tau^2 = 13.8955$; $Chi^2 = 22.47$, df = 6 (P = 0.0010); $I^2 = 73.3\%$								
**subgroup = MDA-MB-231**								
Chang et al. 2022	80.00	5.0000	3	70.00	5.0000	3	7.5%	1.60 [-0.56; 3.75]
Du et al. 2021	50.00	5.0000	3	200.00	10.0000	3	1.5%	-15.14 [-28.98; -1.30]
Eiro et al. 2024	28.13	15.6300	3	100.00	18.7500	3	6.5%	-3.32 [-6.74; 0.09]
Farhadi et al. 2023	34.60	5.8000	3	100.00	5.8000	3	3.1%	-9.00 [-17.32; -0.67]
Khazaei-Poul et al. 2021	49.30	0.0000	3	100.00	0.0000	3	0.0%	
Mohd Ali et al. 2021	45.00	4.0000	3	70.00	5.0000	3	5.8%	-4.41 [-8.71; -0.10]
Pakravan et al. 2017	30.40	5.2000	3	100.00	8.7000	3	3.7%	-7.75 [-14.96; -0.53]
Shojaei et al. 2021	60.00	1.0000	3	85.00	1.0000	3	0.9%	-19.95 [-38.13; -1.77]
Shojaei et al. 2023	70.00	3.0000	3	88.00	2.0000	3	4.9%	-5.63 [-10.99; -0.27]
Vakhshiteh et al. 2021	70.00	3.1000	3	98.00	2.4000	3	3.5%	-8.06 [-15.55; -0.57]
Yang et al. 2022	50.00	5.0000	3	70.00	5.0000	3	6.6%	-3.19 [-6.50; 0.12]
Zhang et al. 2022	27.30	4.5000	3	100.00	4.5000	3	1.9%	-12.89 [-24.70; -1.08]
**Total (95% CI)**			**36**			**36**	**45.9%**	**-5.17 [-7.97; -2.37]**
Heterogeneity: $Tau^2 = 11.5457$; $Chi^2 = 32.47$, df = 10 (P = 0.0003); $I^2 = 69.2\%$								
**subgroup = 4T1**								
Egea et al. 2021	25.00	5.0000	3	100.00	10.0000	3	3.8%	-7.57 [-14.63; -0.51]
Hosseini et al. 2024	37.50	4.0000	3	100.00	1.0000	3	1.2%	-17.10 [-32.72; -1.49]
Lee et al. 2013	35.00	5.0000	3	100.00	5.0000	3	2.6%	-10.37 [-19.93; -0.82]
Zhou et al. 2024	2.00	1.0000	3	9.00	2.0000	3	6.4%	-3.53 [-7.12; 0.05]
**Total (95% CI)**			**12**			**12**	**13.9%**	**-6.82 [-11.41; -2.23]**
Heterogeneity: $Tau^2 = 7.9198$; $Chi^2 = 4.61$, df = 3 (P = 0.2030); $I^2 = 34.9\%$								
**subgroup = SK-BR-3**								
Zhang et al. 2022	95.00	5.0000	3	100.00	5.0000	3	7.8%	-0.80 [-2.55; 0.96]
**Total (95% CI)**			**72**			**72**	**100.0%**	**-4.70 [-6.54; -2.85]**
**Prediction interval**								**[-11.71; 2.32]**
Heterogeneity: $Tau^2 = 10.5687$; $Chi^2 = 67.59$, df = 22 (P < 0.0001); $I^2 = 67.4\%$								
Test for overall effect: Z = -4.99 (P < 0.0001)								
Test for subgroup differences: $Chi^2 = 11.38$, df = 3 (P = 0.0098)								

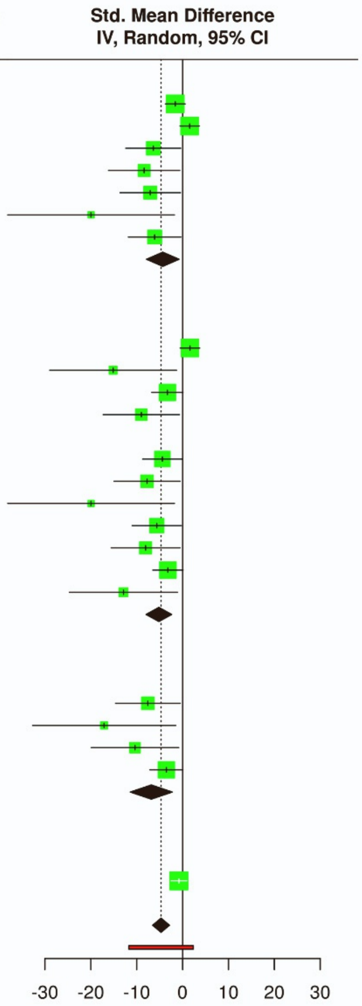

Figure S3. Forest plot of standardized mean differences in breast cancer cell migration following MSC-EV treatment, stratified by breast cancer cell line.

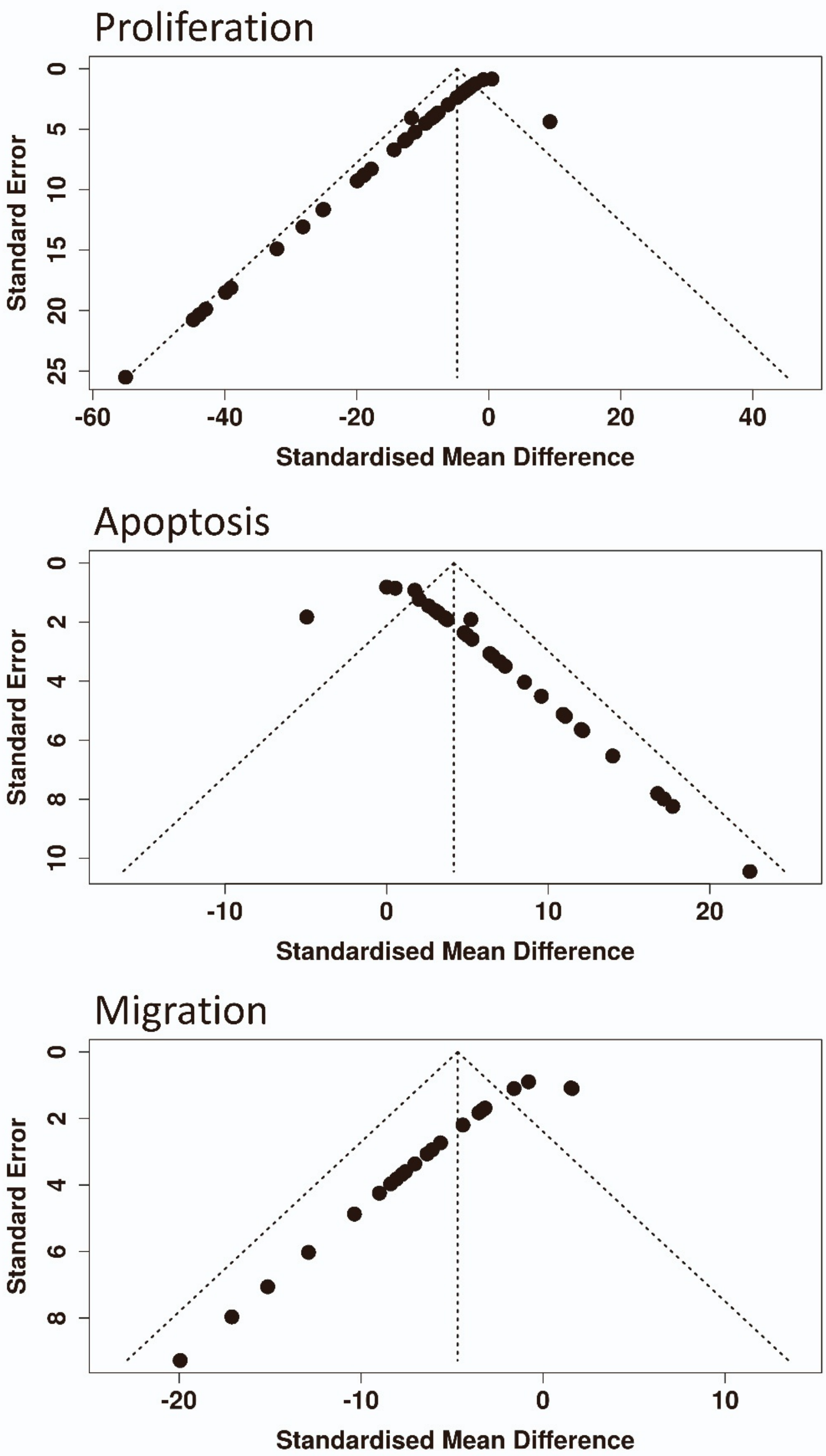

Figure S4. Funnel plots for assessment of publication bias in MSC-EV meta-analyses. Funnel plots and Egger's tests revealed no significant asymmetry for any outcome ($p > 0.05$), suggesting limited publication bias across the analyzed studies. Plots are shown for (A) proliferation/viability, (B) apoptosis, and (C) migration outcomes.

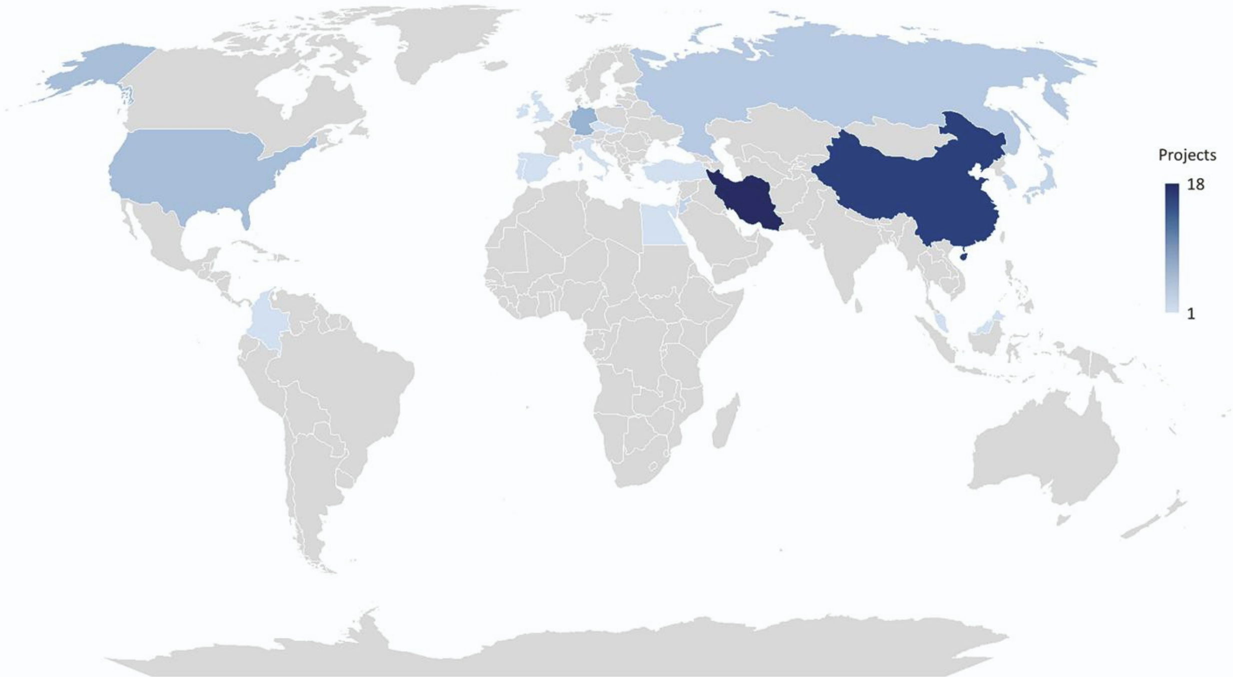

Figure S5. Geographic distribution of MSC-EV studies in breast cancer by country (number of studies per country).
